# Catalyst-controlled regiodivergent ring-opening C(sp^3^)–Si bond-forming reactions of 2-arylaziridines with silylborane enabled by synergistic palladium/copper dual catalysis†
†Electronic supplementary information (ESI) available: Experimental procedures for synthesis and screening, spectroscopic data of new compounds, X-ray crystallographic data, EPR spectra copies of ^1^H and ^13^C NMR charts, and HPLC analysis data. CCDC 1911669. For ESI and crystallographic data in CIF or other electronic format see DOI: 10.1039/c9sc02507c


**DOI:** 10.1039/c9sc02507c

**Published:** 2019-07-31

**Authors:** Youhei Takeda, Kaoru Shibuta, Shohei Aoki, Norimitsu Tohnai, Satoshi Minakata

**Affiliations:** a Department of Applied Chemistry , Graduate School of Engineering , Osaka University , Yamadaoka 2-1, Suita , Osaka 565-0871 , Japan . Email: takeda@chem.eng.osaka-u.ac.jp ; Email: minakata@chem.eng.osaka-u.ac.jp; b Department of Material and Life Science , Graduate School of Engineering , Osaka University , Yamadaoka 2-1, Suita , Osaka 565-0871 , Japan

## Abstract

A catalyst-controlled regiodivergent and stereospecific ring-opening C(sp^3^)–Si cross-coupling of 2-arylaziridines with silylborane enabled by synergistic Pd/Cu dual catalysis has been developed. Just by selecting a suitable combination of catalysts, the regioselectivity of the coupling is completely switched to efficiently provide two regioisomers of β-silylamines (*i.e.*, β-silyl-α-phenethylamines and β-silyl-β-phenethylamines) in good to high yields. Furthermore, a slight modification of the reaction conditions caused a drastic change in reaction pathways, leading to a tandem reaction to produce another regioisomer of silylamine (*i.e.*, α-silyl-β-phenethylamines) in an efficient and selective manner.

## Abstract 2

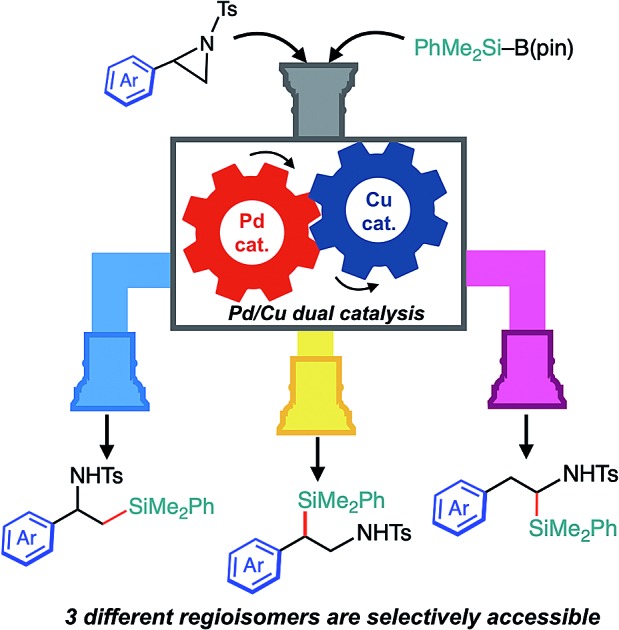
A Pd/Cu catalyst-controlled regiodivergent and stereospecific ring-opening C(sp^3^)–Si cross-coupling of 2-arylaziridines with silylborane has been developed and a new tandem reaction to give another regioisomer of silylamine has been discovered.

## Introduction

Alkylsilanes are ubiquitous motifs in industrial products, and they serve as useful synthetic building blocks.^1^ A wide variety of synthetic methods for alkylsilanes including silyl-S_N_2 reaction,^2^ silyl-Negishi and silyl-Kumada-Tamao reactions of silyl halides,^3^ hydrosilylation of alkenes,^4^ C–H silylation of alkanes,^5^ the addition of Si–E (*e.g.*, E = Si, B) bonds across C=C double bonds,^6^ Cu-catalyzed nucleophilic substitution of alkyltriflates,^7^ and radical-mediated C(sp^3^)–Si coupling of unactivated alkylhalides8a,c and carboxylate derivatives8b,c with silylboranes have been developed. Also, more recently, transition-metal (Ni and Pd)-catalyzed C(sp^3^)–Si cross-coupling of an alkyl electrophile with a silyl (pro)nucleophile (Si–M, M = Zn, B) has been emerging as a powerful synthetic method for alkylsilanes because of high chemoselectivity and functional group tolerance.^9^–^12^ Nevertheless, to date, alkyl electrophiles that are applicable to coupling are limited to branch-type alkyl halides,^9^ benzyl halides,^10^,^11^ and benzyl ethers.^12^ Therefore, the development of C(sp^3^)–Si cross-coupling using hitherto unexplored alkyl electrophiles with a silyl (pro)nucleophile holds great promise for widening the diversity of available alkylsilanes.

**Scheme 1:**
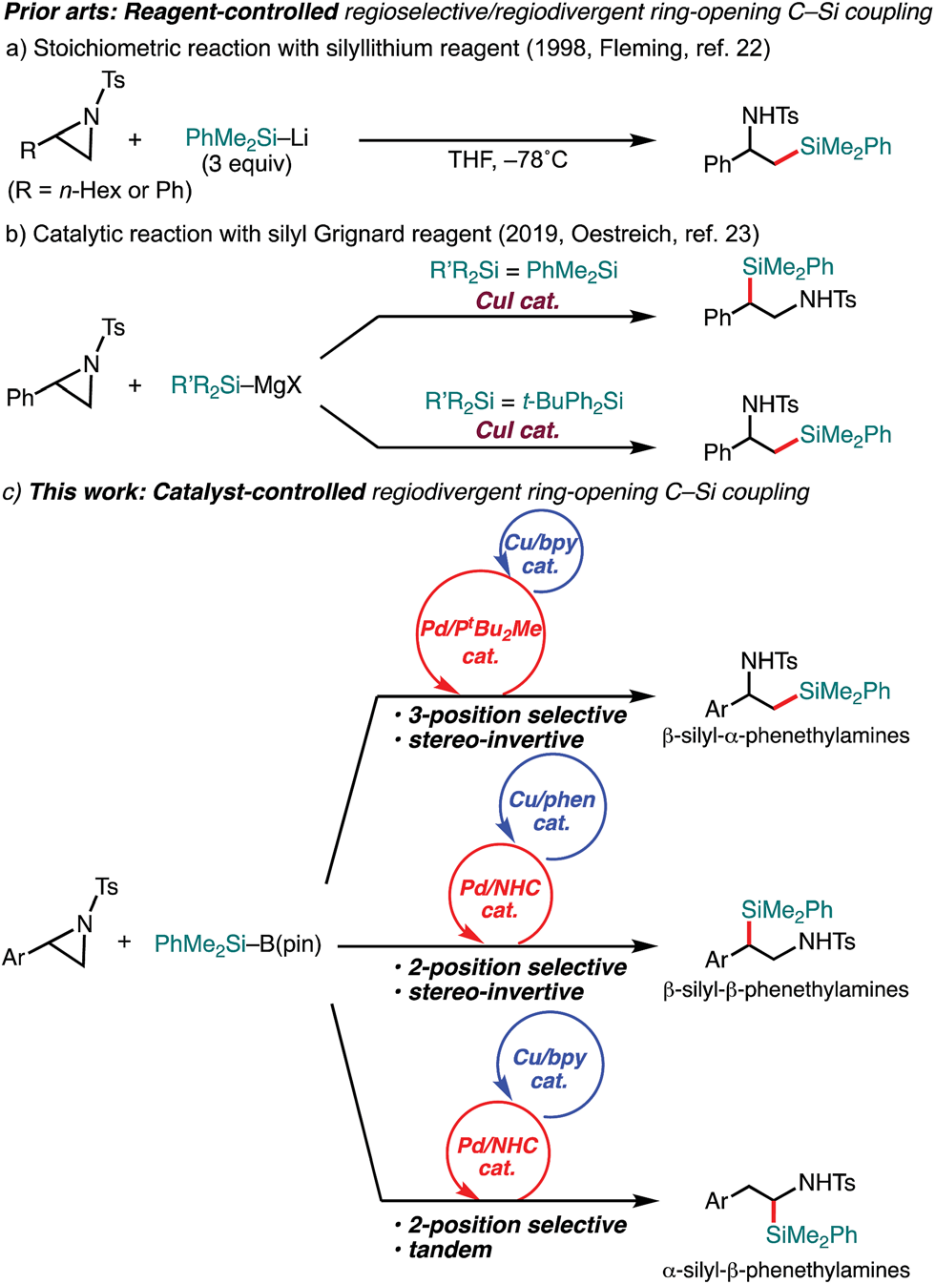
Regioselective C(sp^3^)–Si bond-forming reactions of 2-arylaziridines with silylboranes.

Aziridines have emerged as relatively new alkyl electrophiles in transition-metal-catalyzed regioselective ring-opening C–C^13^–^18^ and C–B^19^,^20^ cross-couplings with organoboron and organozinc nucleophiles to give β-organo- and borylated alkylamines, respectively. In conjunction with the fact that alkylamines bearing a C(sp^3^)–Si bond have been recognized as unique bioisosteres of pharmacological agents in medicinal chemistry,^21^ regiodivergent ring-opening C(sp^3^)–Si cross-coupling of aziridines with a silyl (pro)nucleophile would open up a new avenue to the preparation of a set of regioisomeric β-silyl-alkylamines. A seminal work on regioselective ring opening of aziridines with a silyl nucleophile was reported by Fleming, where silyllithium was applied as a nucleophile (Scheme 1a).^22^ The reaction exclusively gives one regioisomer of silylamine (β-silyl-α-substituted ethylamines); however, the regiochemistry (opening at the 3-position) seems to be governed by the combination of reagents, and it requires excess amounts of silyllithium (3 equivalents) that can deteriorate electron-withdrawing functionalities.^22^ During the preparation of this manuscript, as a related study to our present work, a Cu-catalyzed *reagent-controlled* regiodivergent nucleophilic ring opening of 2-arylaziridines with silyl Grignard reagents has been reported by the Oestreich group (Scheme 1b).^23^ Although an example using a silylborane (Me_2_PhSi–Bpin) as a pronucleophile was also shown in the same paper to give 3-position selective silylative ring opening coupling,^23^ as is generally the case, the reagent-controlled approach quite limits the diversity of products. To the best of our knowledge, *catalyst-controlled* regiodivergent ring-opening C(sp^3^)–Si cross-coupling of aziridines has not been achieved yet. Herein, we disclose a *catalyst-controlled* regiodivergent and stereospecific ring-opening C(sp^3^)–Si cross-coupling of 2-arylaziridines with a silylborane to selectively give two different regioisomers of silylphenethylamines enabled by Pd/Cu synergistic dual catalysis (the upper and middle equations, Scheme 1c).^24^ Notably, a catalytic tandem reaction to give another regioisomer of silylamines was also discovered (the bottom equation, Scheme 1c).

## Results and discussion

We previously reported that NHC/Pd and P*t*Bu_2_Me/Pd catalysts allow for regioselective and stereospecific (stereo-invertive) ring-opening Suzuki–Miyaura arylation and borylation of 2-arylaziridines13*a* at the 2- and 3-positions, respectively.^19^ Theoretical calculations on the mechanisms suggested that the regioselectivity and stereospecificity are determined in an S_N_2-type oxidative addition step through the interaction between the catalyst and aziridine.13b,^19^ Based on the borylation conditions,^19^ as an initial attempt, racemic aziridine **1a** was treated with silylborane (Me_2_PhSi–Bpin, **2**) (1.2 equiv.) in the presence of Cp(allyl)Pd/P^*t*^Bu_2_Me and bipyridine (bpy) catalysts in a CPME/H_2_O co-solvent. Although the expected coupled product **3a** was formed, the chemical yield was very low even at an elevated temperature (Table 1, entry 2), suggesting that the transmetalation between Si–B and Pd–OMe species would be energetically much higher than that of the C–B coupling. At a temperature higher than 60 °C, β-hydride elimination from the oxidative adduct was accelerated,^19^ indicating the difficulty of identifying suitable reaction conditions for the coupling with a single metallic catalytic system (for details, see Tables S1–S8, ESI†). We envisaged that the addition of a Cu salt could allow for stepwise transmetalation (*i.e.*, Si–B → Si–Cu → Si–Pd) to promote the coupling, and the effect of Cu additives was investigated (Table S4, ESI†). Importantly, the addition of CuSO_4_ drastically improved the chemical yield of **3a** up to 89% (Table 1, entry 1 *vs.* entry 2). Other copper salts such as CuF_2_ and Cu(OH)_2_ gave **3a** in comparable yields (Table 1, entries 3 and 4), while the addition of CuCl resulted in a lower yield (Table 1, entry 5), possibly due to the poorer solubility of the salt in the solvents. Without the Pd complex and phosphine ligand, no reaction occurred (Table 1, entries 6 and 7), excluding the possibility of direct nucleophilic ring-opening substitution with Cu–Si species.^23^ Furthermore, bpy should play an important role in the coupling, because coupled product **3a** was not produced at all in the absence of bpy (Table 1, entry 8). In addition, β-methoxy-β-phenethylamine **7** was produced in a substantial yield, which should be produced through the nucleophilic ring opening of **1a** with MeOH under the electrophilic activation of **1** by the Cu Lewis acid,^25^ implying that bpy adjusts the Lewis acidity of the Cu additive to suppress the background side reaction. Another possible role is to enhance the solubility of the Cu-species by coordination to increase the concentration of the active Cu species. A proton source was required for the reaction to smoothly proceed (Table 1, entries 9 and 10), indicating that the M–OMe species (M = Pd, Cu) generated *in situ* would promote stepwise transmetalation (Si–B → Si–Cu → Si–Pd) through Lewis acid–base interactions. The C(sp^3^)–Si coupling proceeds smoothly under neutral conditions without adding any explicit strong Lewis bases, which are usually required to activate silylboranes to transfer the silyl unit.^7^,8a,b,^10^,^11^ Other silylboranes such as Ph_2_MeSi–Bpin, Ph_2_*t*BuSi–Bpin, and Et_3_Si–Bpin did not give any silylated products.

**Table 1:** Effect of reaction parameters on the 3-position-selective C(sp^3^)–Si cross-coupling of **1a** with **2**^*a*^

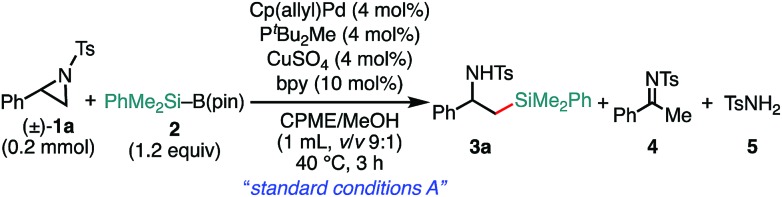					
Entry	Variations from the “*standard conditions A*”	Yield (%)			Recovery of **1a** (%)
		**3a**	**4**	**5**	
1	None	89	6	0	0
2^*b*^	w/o CuSO_4_	13	Trace	39	46
3	CuF_2_ in place of CuSO_4_	83	0	5	0
4	Cu(OH)_2_ in place of CuSO_4_	83	0	7	0
5	CuCl	35	0	4	55
6	w/o Cp(ally)Pd and P*t*Bu_2_Me	0	0	0	99
7	w/o Cp(allyl)Pd	0	0	0	99
8^*c*^	w/o bpy	0	23	0	13
9	H_2_O in place of MeOH	75	4	0	8
10	w/o MeOH	Trace	0	0	93
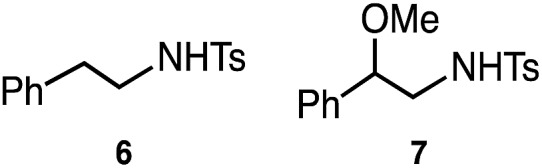					

^*a*^The “*standard conditions A*”: **1a** (0.2 mmol), **2** (0.24 mmol), Cp(allyl)Pd (8 μmol), P*t*Bu_2_Me (8 μmol), CuSO_4_ (8 μmol), and bpy (20 μmol) were stirred in CPME/MeOH (1 mL, v/v 9 : 1) at 40 °C for 3 h.

^*b*^The reaction was conducted at 60 °C.

^*c*^Phenethylamines **6** and **7** were obtained in 12% and 51%, respectively.

With the optimized reaction conditions in hand, the substrate scope of aziridines was investigated (Table 2). A variety of 2-arylaziridines **1** having a functional group on the aromatic ring were applicable, giving β-silyl-α-phenethylamines **3** in a regioselective manner in good to high yields. In particular, it is noted that chlorine and ester functionalities tolerated the reaction conditions to give the corresponding coupling products (**3d**, **3f**, **3g**, and **3h**). On the other hand, the reactions with 2,3-disubstituted (**1j**), 2-alkylated (**1k**), 2-(*o*-Br–C_6_H_4_)–substituted (**1l**), and cyclic (*cis*-**1m**) aziridines were not successful (Table 2). Since *N*-tosyl-2-(*p*-Br–C_6_H_4_)–aziridine was also not applicable to the reaction conditions (recovery of aziridine 87%), the reason why the coupling reaction using **1l** did not proceed would be the electronic effect rather than the steric effect of the *o*-Br substituent. The preference of the oxidative addition of the C–Br bond to the Pd(0) complex might be the side reaction which inhibits the desired reaction.

**Table 2:** Scope of aziridines in the 3-position-selective C(sp^3^)–Si cross-coupling^*a*^

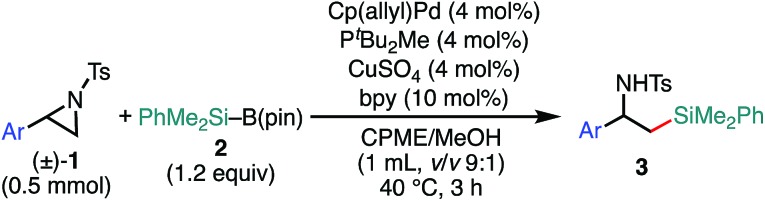
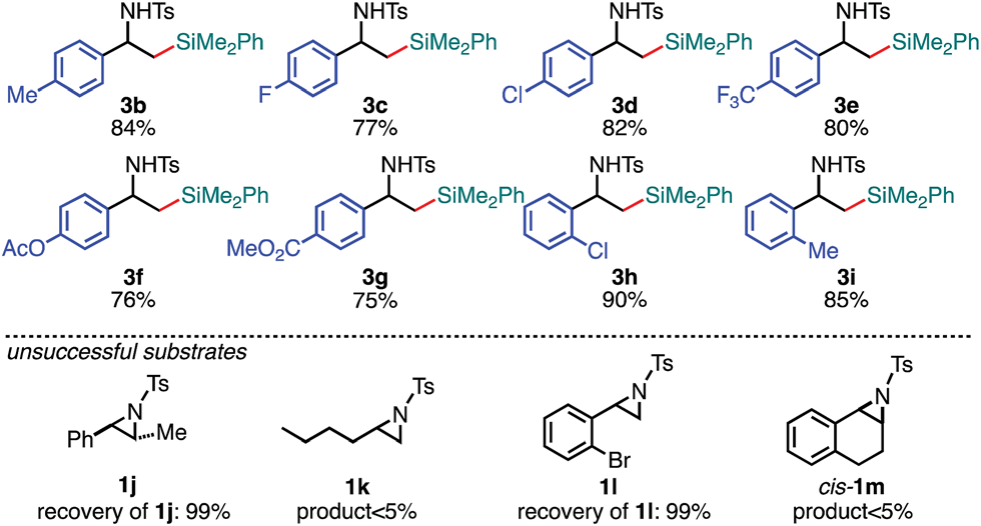

^*a*^The reaction was conducted at 0.50 mmol (**1**) scale under “*standard conditions A*”.

To gain stereochemical information about the reaction, a deuterated aziridine (*cis*-**1a**-*d*_1_) was coupled with silylborane under the *standard conditions A* [eqn (1)]. Derivatization of the coupled product **3a**-*d*_1_ (for detailed procedures to determine the relative stereochemistry, see Scheme S1, ESI†) revealed that **3a**-*d*_1_ has the *trans*-configuration, indicating that the C(sp^3^)–Si cross-coupling proceeds in a stereo-invertive manner. This indicates that the regio- and stereospecificity-determining step would be an S_N_2-type oxidative addition even in the dual catalytic system.
1

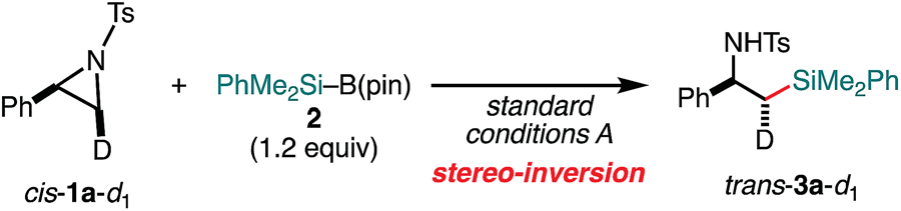




Delighted with the validity of the Pd/Cu dual catalysis in the 3-position-selective C(sp^3^)–Si coupling, we turned our attention to switching the regioselectivity of the ring-opening silylative coupling. Although NHC-ligated Pd(ii) complexes are suitable pre-catalysts for Suzuki–Miyaura arylation of **1a**,13*a* the attempts with those Pd(ii) complexes failed only to recover the aziridine, indicating the weaker nucleophilic ability of the silylborane to reduce Pd(ii) to Pd(0) than arylboronic acid.13*b* To circumvent the reduction process, we applied NHC-ligated Pd(0) complexes^26^ as Pd pre-catalysts and found them to be successful: a coupling reaction proceeded exclusively at the 2-position to give the regioisomeric coupled product (β-silyl-β-phenethylamine) (for detailed results, see Tables S9–S16, ESI†). Among the tested, a catalytic system comprising SIPr–Pd(0)–PPh_3_, CuF_2_, and 1,10-phenanthroline (phen) was found to be suitable for the 2-position-selective ring-opening C(sp^3^)–Si coupling to give **8a** in a high yield (*standard conditions B*) [eqn (2)]. Intriguingly, along with **8a**, 11% of α-silyl-β-phenethylamine **9a**, which is another regioisomer of **8a**, was produced [eqn (2)]. Although the detailed mechanism leading to **9a** should await further study, a putative pathway involves tandem processes comprising (i) a Pd-catalyzed isomerization of **1a** into aldimines through the oxidative addition of **1a** into the Pd(0) complex at the 2-position followed by β-hydride elimination/tautomerization^27^ and (ii) a subsequent nucleophilic attack of the resulting aldimines by the Cu–Si species generated *in situ* (*vide infra*).^28^ Delighted with the discovery of this hitherto unknown reaction, we surveyed the effect of reaction parameters on the product distribution (for details, see the ESI†). It turned out that the tandem reaction was drastically promoted by simply adding extra PPh_3_ and changing the additive from phen to bpy (Tables S10 and S16, ESI†), which led to exclusive and quantitative formation of **9a** (*standard conditions C*) [eqn (3)].
2

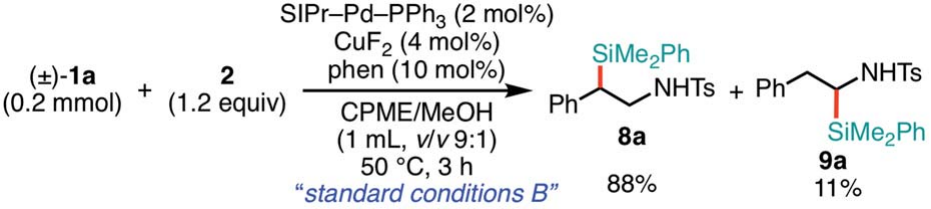



3

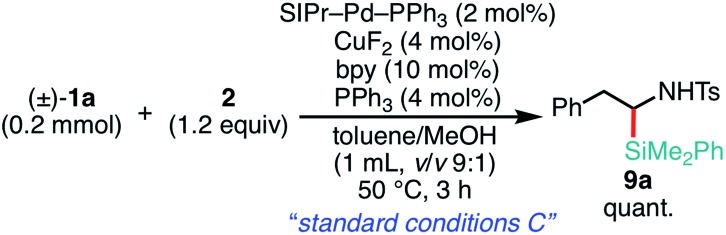




The scope of the 2-position-selective ring-opening C(sp^3^)–Si cross-coupling was investigated (Table 3). The cross-coupling of enantiopure aziridine (*R*)-**1a** (>99% ee) under the *standard conditions B* proceeded regioselectively and enantiospecifically to give enantiopure product **8a** in 89% yield (99% ee). The absolute configuration of **8a** was unambiguously determined to be *S* by the single crystal X-ray diffraction analysis (Table 3, the inset figure; for detailed crystallographic data, see Table S17, ESI†). This indicates that the coupling proceeds with stereo-inversion, which is fully consistent with an S_N_2-type oxidative addition of aziridine.13*a* The coupling conditions were applicable to 2-arylaziridines bearing a variety of functional groups, giving rise to the corresponding coupled products in good to high yields in a regioselective and stereospecific manner (Table 3). Again, the reactions using aziridines **1j–1m** were not successful.

**Table 3:** Scope of aziridines in the 2-position-selective C(sp^3^)–Si cross-coupling^*a*^

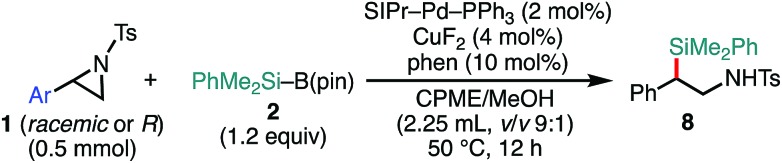
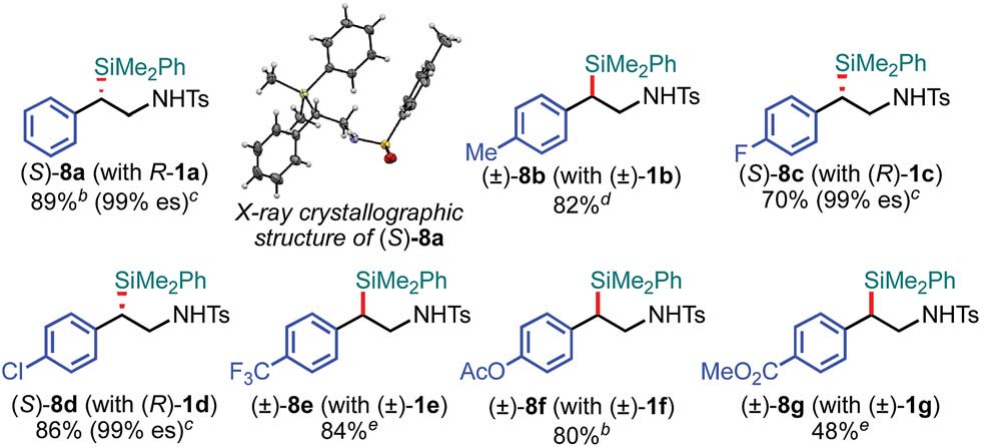

^*a*^The reaction was conducted at 0.50 mmol (**1**) scale under the “*standard conditions B*”.

^*b*^The reaction was run for 3 h.

^*c*^es (enantiospecificity) = ee (**8**)/ee (**1**) × 100; ee (enantiomeric excess) was determined by chiral HPLC analysis.

^*d*^The reaction was conducted with the ^Me^IPr–Pd–PPh_3_ catalyst at 60 °C.

^*e*^The reaction was conducted at room temperature.

The substrate scope of the tandem C(sp^3^)–Si bond-forming reaction was also investigated (Table 4). A variety of 2-arylaziridines with a functional group were efficiently converted into the corresponding α-silyl-β-phenethylamine products, which are often found in bioisosteres of protease.^21^

**Table 4:** Scope of aziridines in the C(sp^3^)–Si bond-forming tandem reaction^*a*^

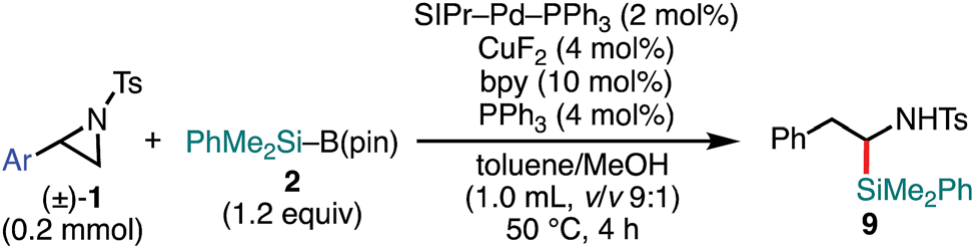
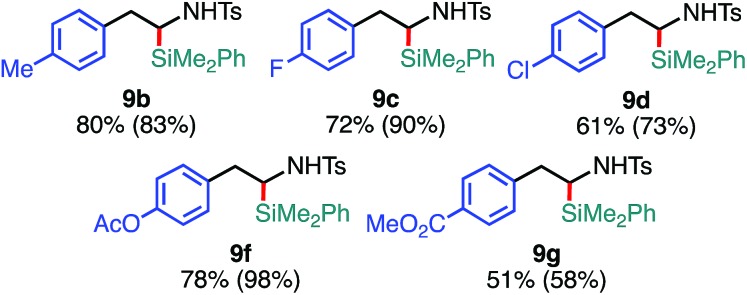

^*a*^The reaction was conducted at 0.20 mmol (**1**) scale under the “*standard conditions C*”.

**Scheme 2:**
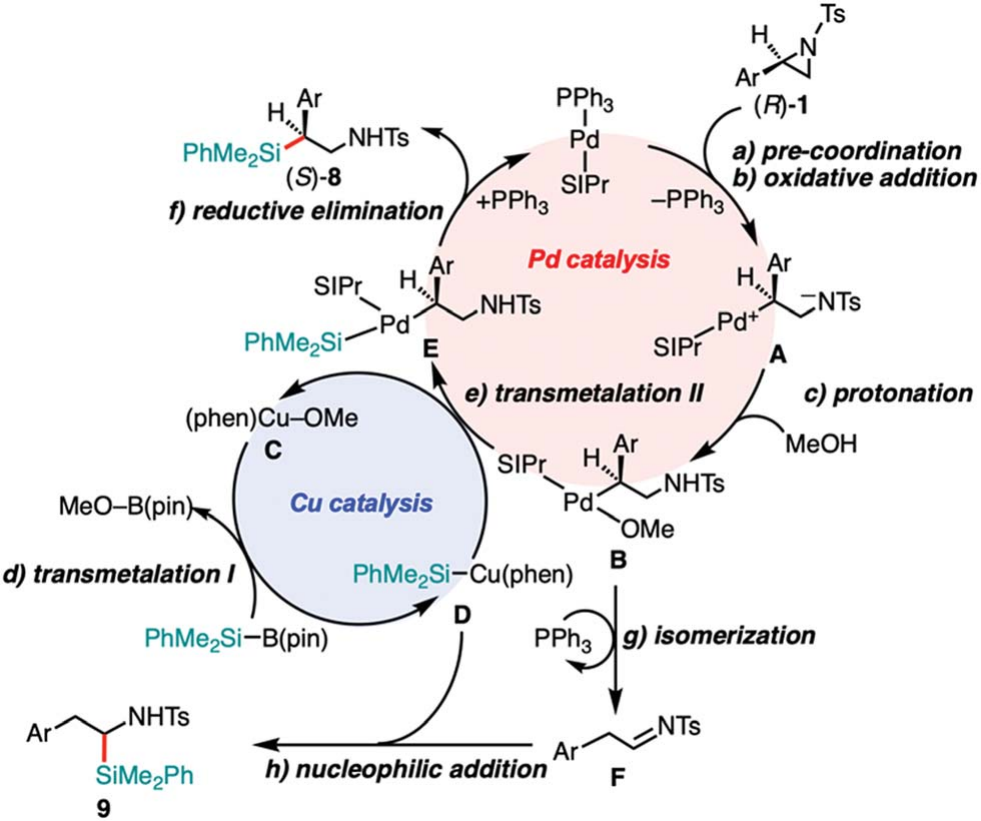
A proposal of dual catalysis in the 2-position-selective C(sp^3^)–Si cross-coupling and tandem reaction.

A proposed reaction mechanism of the 2-position-selective C(sp^3^)–Si coupling and the tandem reaction are illustrated in Scheme 2. The catalytic cycle would start from the pre-coordination of SIPr–Pd(0) to the aryl moiety of **1** to stabilize the complex (Scheme 2a),13*b* which then undergoes oxidative addition from the backside of the C(2)–N(1) bond with stereo-inversion (Scheme 2b). The regioselectivity for the ring opening would be determined by the pre-coordination of the Ar moiety to the Pd center to gain large interaction energy (INT), which was reasonably suggested by the energy decomposition analysis (EDA) of the computed oxidative addition process of the aziridines into a Pd(0)–NHC complex.13*b* In sharp contrast, in the case of the phosphine-Pd catalyst system, the oxidative addition takes place on the opposite side (*i.e.*, C(3)–N(1)) in a regioselective fashion. This would be caused by the larger deformation energy (DEF) than INT gained when the V-shaped bisphosphine–Pd complex [(*t*Bu)_2_MeP–Pd(0)–PMe(*t*Bu)_2_] approaches from the backside of the C(2)–N(1) bond.^19^ Therefore, we can conclude that the regioselectivity of the ring opening would be mainly governed by the balance between (i) how large interactions between the Pd catalyst and aziridine operate and (ii) how large deformations the Pd catalyst and aziridine experience when they approach each other. The resulting zwitterionic oxidative adduct **A** can be protonated with MeOH to form alkoxide complex **B** (Scheme 2c).^19^ This intermediate would then undergo transmetalation with the PhMe_2_Si–Cu(phen) complex **D**, giving an alkyl(silyl)Pd complex **E** (Scheme 2e), where silylcopper **D** could be generated from the transmetalation between Cu–OMe species **C** with silylborane (Scheme 2d).^29^ The reactions of **1a** with the silylborane in the presence of Cu catalysts in different oxidation states [(phen)Cu^I^F30*a* and (phen)Cu^II^F_2_ 30*b*] gave **8a** in almost the same yields (see Table S15, ESI†), implying that the oxidation level of the actual Cu species under the optimized conditions would be either Cu^I^ or Cu^II^. To probe the oxidation level of the actual Cu active species, the reaction mixtures starting with a Cu^I^ and Cu^II^ additive were monitored with an electron paramagnetic resonance (EPR) technique. From the results, the concentration of Cu^II^ species was found to be significantly decreased under the optimized conditions (see Fig. S1 in the ESI†), indicating that the reduction of Cu^II^ to Cu^I^ occurs *in situ*. Taken together, we assume that the putative Cu active species in Cu catalysis is (phen)Cu^I^–L (L = F or OMe). The reductive elimination from **E** should produce **8** and regenerate the Pd(0) catalyst (Scheme 2f). Almost the same dual catalysis would be involved in the C–Si cross-coupling at the 3-position, except for the 3-position-selective oxidative addition.^19^ A possible pathway to **9** would involve (i) a PPh_3_/Pd-catalyzed isomerization of **1** into aldimine **F***via* β-hydride elimination and tautomerization (Scheme 2g)^27^ and (ii) the following nucleophilic addition of Si–Cu species **D** to the imine (Scheme 2h).^28^ Since the treatment of separately prepared aldimine **F** with silylborane **2** under similar reaction conditions (“*standard conditions C*”) gave **9** in a moderate yield (when Ar = Ph, 31% of **9a** was obtained with no recovery of the aldimine substrate; for details, see Scheme S2, ESI†), this scenario is likely to occur.

## Conclusions

In conclusion, we have succeeded in developing catalyst-controlled highly regiodivergent ring-opening C–Si bond formation reactions to selectively provide three regioisomers of silylamines *via* synergistic Pd/Cu dual catalysis. It is noted that the balance between the efficiency of Pd and Cu catalysis should be the origin of the discovery of the tandem reaction, and this knowledge would provide us with insights for designing more intricate and sophisticated transformations in the future. Detailed catalytic reaction mechanisms are investigated experimentally and theoretically in our laboratory.

## Conflicts of interest

There are no conflicts to declare.

## Supplementary Material

Supplementary information

Crystal structure data
