# Supplementary material for: Catalyst-controlled regiodivergent ring-opening C(sp3)–Si bond-forming reactions of 2-arylaziridines with silylborane enabled by synergistic palladium/copper dual catalysis
Source: Chem Sci. 2019 Jul 31;10(37):8642–7. doi: 10.1039/c9sc02507c (PMC6844297; doi:10.1039/c9sc02507c)
Supplement: Supplementary file 1 [file SC-010-C9SC02507C-s001.pdf]

Electronic Supplementary Material (ESI) for Chemical Science.
This journal is © The Royal Society of Chemistry 2019

Electronic Supplementary Information for

Catalyst-Controlled Regiodivergent Ring-Opening C(sp^3)–Si Bond-Forming Reactions of 2-Arylaziridines with Silylborane Enabled by Synergistic Palladium/Copper Dual Catalysis

Youhei Takeda,*,† Kaoru Shibuta,† Shohei Aoki,† Norimitsu Tohnai,‡ and Satoshi Minakata*,†

†*Department of Applied Chemistry, Graduate School of Engineering, Osaka University, Yamadaoka 2-1, Suita, Osaka 565-0871, Japan.*

‡*Department of Material and Life Science, Graduate School of Engineering, Osaka University, Yamadaoka 2-1, Suita, Osaka 565-0871, Japan*

e-mail: takeda@chem.eng.osaka-u.ac.jp; minakata@chem.eng.osaka-u.ac.jp

Table of Contents

General remarks	S2
Materials	S2
Preparation of racemic 2-arylaziridines	S2–S4
Preparation of enantiopure 2-arylaziridines	S4–S5
Preparation of deuterated aziridine	S5
The effect of reaction parameters on ring-opening C(sp^3)–Si bond-forming reactions	S5–S17
EPR experiments	S18
Spectroscopic data of coupling products	S18–S28
Control Experiment	S29
Single crystal X-ray crystallographic data of (*S*)-**8a**	S29–S30
Copies of ^{1}H and ^{13}C NMR charts	S31–S54
HPLC charts	S55–S60
References	S61

General remarks. All reactions were carried out under an atmosphere of nitrogen using standard Schlenk technique or glove box. Melting points were determined on a Stanford Research Systems MPA100 OptiMelt Automated Melting Point System. Infrared spectra were acquired on a SHIMADZU IRAffinity-1 FT-IR Spectrometer. All ^{1}H, ^{13}C and ^{29}Si NMR spectra were recorded on a JEOL JMTC-400/54/ss NMR Spectrometer (^{1}H NMR, 400 MHz; ^{13}C NMR, 100 MHz; ^{29}Si NMR, 79 MHz), and chemical shifts were referenced to the signal of an internal standard (tetramethylsilane, δ = 0 ppm for ^{1}H,^{13}C and ^{29}Si NMR measurements) or an external standard. Chiral-phase high-performance liquid chromatography (HPLC) was performed on a SHIMADZU prominence series instruments equipped with chiral columns. Low- and high-resolution mass spectra were acquired on a JEOL JMS-DX303HF mass spectrometer. Analytical thin-layer chromatography (TLC) was performed on pre-coated silica gel glass plates (Merck silica gel 60 F_{254}, 0.25 mm thickness), and compounds were visualized with UV lamp or treatment with an ethanolic solution of phosphomolybdic acid followed by heating. Products were purified by flash column chromatography on a silica gel BW-300 (Fuji Silysia Chemical Ltd).

Materials. All solvents were purchased from commercial sources and used after dry and distillation. Styrene derivatives, (pin)B–$SiMe_2Ph$, P(*t*-Bu)$_2$Me and other commercial reagents were purchased from Sigma Aldrich or TCI and used as received. Cp(allyl)Pd[S1], Cp(cinnamyl)Pd[S2]and NHC–Pd(0)–PPh_3 (NHC = SIPr, IPr and ^{Me}IPr)[S3] were prepared according to the procedures reported in literatures.

Preparation of racemic 2-arylaziridines. All aziridines **1a–1m** were prepared according to the procedures reported in literature, and their spectroscopic data were in good agreement with those previously reported as follows:

2-Phenyl-1-tosylaziridine (1a) [CAS No. 24395-14-0]

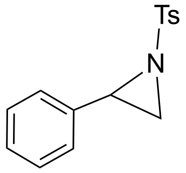

Prepared through aziridination of styrene according to the procedure described in literature;[S4] Spectroscopic data were in good agreement with those previously reported;[S5] Purified by recrystallization from MeOH; 74% yield.

2-(*p*-Tolyl)-1-tosylaziridine (1b) [CAS No.97401-87-1]

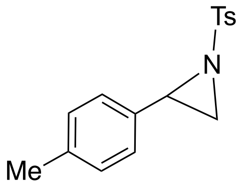

Prepared through aziridination of 1-methyl-4-vinylbenzene according to the procedure described in literature;[S6] Spectroscopic data were in good agreement with those previously reported;[S7] Purified by silica gel column chromatography (*n*-hexane/EtOAc 99:1 to 7:3) and recrystallization from EtOAc; 61% yield.

2-(4-Fluorophenyl)-1-tosylaziridine (1c) [CAS No.25026-25-4]

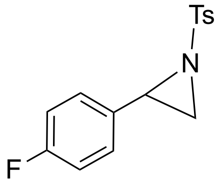

Prepared through aziridination of 1-fluoro-4-vinylbenzene according to the procedure described in literature;[S6] Spectroscopic data were in good agreement with those previously reported;[S7] Purified by silica gel column chromatography (*n*-hexane/EtOAc 99:1 to 7:3) and recrystallization from EtOAc; 61% yield.

2-(4-Chlorophenyl)-1-tosylaziridine (1d) [CAS No.97401-93-9]

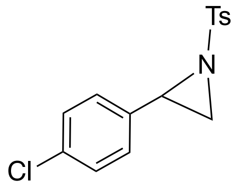

Prepared through aziridination of 1-chloro-4-vinylbenzene according to the procedure described in literature;[S6] Spectroscopic data were in good agreement with those previously reported;[S5] Purified by silica gel column chromatography (*n*-hexane/EtOAc 99:1 to 7:3) and recrystallization from EtOAc; 53% yield.

1-Tosyl-2-(4-(trifluoromethyl)phenyl)aziridine (1e) [CAS No.250260-27-6]

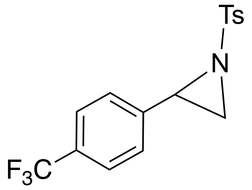

Prepared through aziridination of 1-(trifluoromethyl)-4-vinylbenzene according to the procedure described in literature;[S6] Spectroscopic data were in good agreement with those previously reported;[S7] Purified by silica gel column chromatography (*n*-hexane/EtOAc 99:1 to 7:3) and recrystallization from EtOAc; 65% yield.

4-(1-Tosylaziridin-2-yl)phenyl acetate (1f) [CAS No.250260-26-5]

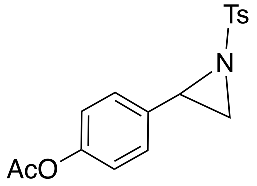

Prepared through aziridination of 4-vinylphenyl acetate according to the procedure described in literature;[S6] Spectroscopic data were in good agreement with those previously reported;[S7] Purified by silica gel column chromatography (*n*-hexane/EtOAc 99:1 to 7:3) and recrystallization from EtOAc; 48% yield.

Methyl 4-(1-tosylaziridin-2-yl)benzoate (1g) [CAS No.1365842-12-1]

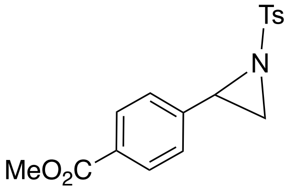

Prepared through aziridination of methyl 4-vinylbenzoate according to the procedure described in literature;[S6] Spectroscopic data were in good agreement with those previously reported;[S5] Purified by silica gel column chromatography (*n*-hexane/EtOAc 99:1 to 7:3) and recrystallization from EtOAc; 72% yield.

2-(2-Chlorophenyl)-1-tosylaziridine (1h) [CAS No.1227184-73-7]

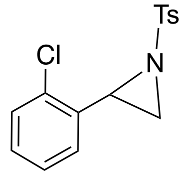

Prepared through aziridination of 1-chloro-2-vinylbenzene according to the procedure described in literature;[S6] Spectroscopic data were in good agreement with those previously reported;[S8] Purified by silica gel column chromatography (*n*-hexane/EtOAc 99:1 to 7:3) and recrystallization from EtOAc; 66% yield.

2-(*o*-Tolyl)-1-tosylaziridine (1i) [CAS No.1111321-35-7]

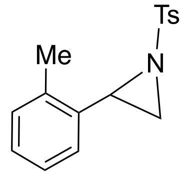

Prepared through aziridination of 1-methyl-2-vinylbenzene according to the procedure described in literature;[S6] Spectroscopic data were in good agreement with those previously reported;[S7] Purified by silica gel clumn chromatography (*n*-hexane/EtOAc 99:1 to 7:3) and recrystallization from EtOAc; 36% yield.

***trans*-2-Methyl-3-phenyl-1-tosylaziridine (1j)** [CAS No. 137595-21-2]

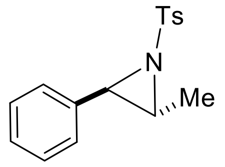

Prepared through aziridination of (*E*)-propenylbenzene according to the procedure described in literature;[S10] Spectroscopic data were in good agreement with those previously reported;[S9] Purified by silica gel column chromatography (*n*-hexane/EtOAc 99:1 to 7:3) and recrystallization from EtOAc; 51% yield.

2-Butyl-1-tosylaziridine (1k) [CAS No.116905-61-4]

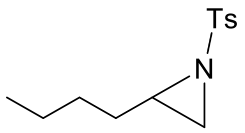

Prepared through aziridination of 1-hexene according to the procedure described in literature;[S10] Spectroscopic data were in good agreement with those previously reported;[S11] Purified by silica gel clumn chromatography (hexane/EtOAc 99:1 to 7:3); 42% yield.

2-(2-Bromophenyl)-1-tosylaziridine (1l) [CAS No.200803-17-4]

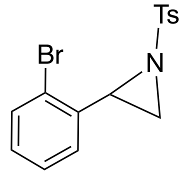

Prepared through aziridination of 1-bromo-2-vinylbenzene according to the procedure described in literature;[S6] Spectroscopic data were in good agreement with those previously reported;[S8] Purified by silica gel column chromatography (*n*-hexane/EtOAc 99:1 to 7:3) and recrystallization from EtOAc; 60% yield.

***cis*-1-Tosyl-1a,2,3,7b-tetrahydro-1*H*-naphtho[1,2-*b*]azirine (*cis*-1m)** [CAS No.200803-17-4]

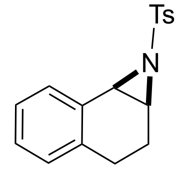

Prepared through aziridination of 1,2-dihydronaphthalene according to the procedure described in literature;[S6] Spectroscopic data were in good agreement with those previously reported;[S8] Purified by silica gel column chromatography (*n*-hexane/EtOAc 99:1 to 7:3); 53% yield.

Preparation of enantiopure 2-arylaziridines. All enantiopure aziridines were prepared according to the procedures reported in literature, and their spectroscopic data were in good agreement with those previously reported as follows:

(*R*)-2-Phenyl-1-tosylaziridin [(*R*)-1a] [CAS No. 62596-62-7]

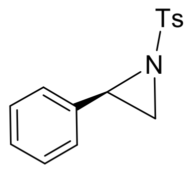

Prepared from (*R*)-phenylglycinol through cyclization according to the procedure described in literature;[S12] Spectroscopic data were in good agreement with those previously reported;[S5] Purified by silica gel column chromatography (*n*-hexane/EtOAc, 99:1 to 7:3); 90% yield, 99% ee (HPLC); HPLC (Chiralcel OJ; 1.0 mL/min; *i*-PrOH/*n*-hexane 30:70; λ = 254 nm): t_R 19.1 min.

(*R*)-2-(4-Fluorophenyl)-1-tosylaziridine [(*R*)-1c] [CAS No.676464-83-8]

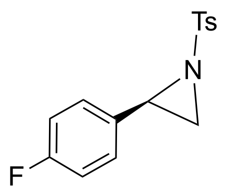

Prepared through asymmetric aziridination of 1-fluoro-4-vinylbenzene according to the procedure described in literature;[S13] Spectroscopic data were in good agreement with those previously reported;[S5] Purified by silica gel column chromatography (*n*-

hexane/EtOAc 99:1 to 7:3) and recrystallization from EtOAc; 29% yield, 99% ee (HPLC); HPLC (Chiralcel OJ; 1.0 mL/min; *i*-PrOH/*n*-hexane 30:70; l = 254 nm): t_R 10.9 min.

(*R*)-2-(4-Chlorophenyl)-1-tosylaziridine [(*R*)-1d] [CAS No.676464-84-9]

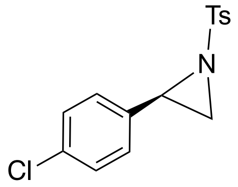

Prepared through asymmetric aziridination of 1-chloro-4-vinylbenzene according to the procedure described in literature;[S13] Spectroscopic data were in agreement with those previously reported;[S5] Purified by silica gel column chromatography (hexane/EtOAc 99:1 to 7:3) and recrystallization from EtOAc; 23% yield, 99% ee (HPLC); HPLC (Chiralcel OJ; 1.0 mL/min; *i*-PrOH/*n*-hexane 30:70; l = 254 nm): t_R 10.6 min.

Preparation of deuterated aziridine.

***cis*-2-Deuterium-2-phenyl-1-tosylaziridine (*cis*-1a-*d*$_1$)** [CAS No.320750-88-7]

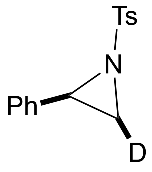

Prepared according to the procedure described in literature;[S14] Spectroscopic data were in good agreement with those previously reported;[S14] Purified by silica gel column chromatography (*n*-hexane/EtOAc, 99:1 to 8:2) and recrystallization from EtOAc; 21% yield.

The Effect of Reaction Parameters on Ring-Opening C(sp^3)–Si Bond-Forming Reactions

1. Ring-opening C(sp^3)–Si cross-coupling at the 3-position.

1-1. Effect of Pd catalyst and ligand

A typical procedure. In a glove box, to a 3 mL vial (vial **A**) with a magnetic stir (10 mm) bar, were added Pd catalyst (2 mol% of Pd), Ligand (4 mol%), and CPME (100 µL). On an aluminum heating block, the resulting solution was stirred at 60 °C (the temperature of heating plate) for 10 min. In another vial (3 mL) (vial **B**), aziridine **1a** (54.6 mg, 0.20 mmol), (pin)B–SiMe$_2$Ph **2** (62.8 mg, 0.24 mmol, 1.2 equiv), 2,2'-bipyridine (6.2 mg, 40 µmol, 20 mol%), and CPME (500 µL) were added. After allowing the vial **A** cool to room temperature, the content of vial **A** was transferred to vial **B**, and the vial **B** was capped with a hole cap and a Teflon®/rubber septum and then transferred from the glove box. Deionized H_2O (100 µL) was added through the septum under a stream of N_2 gas, and the resulting mixture was stirred at 80 °C on an aluminum heating block for 3 h. The reaction mixture was filtrated through the Celite pad (2.0 cm height), and the residue was washed with CH_2Cl_2 (10 mL × 3). The filtrate was dried by evaporating the solvents under vacuum. Yields of products were estimated based on ^{1}H NMR integration of the crude product using 1,1,2,2-tetrachloroethane as an internal standard.

Table S1. Effect of Pd catalyst and ligand

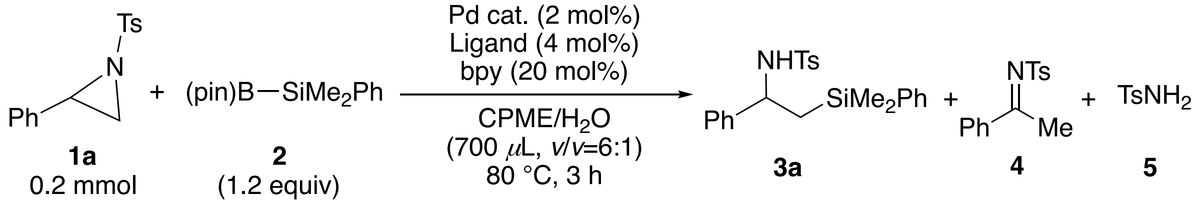

entry	Pd cat.	Ligand	yield (%)[a]			recovery (%)[a]
			**3a**	**4**	**5**	
1	$Pd(OAc)_2$	P^tBu_2Me	0	61	37	0
2	$Pd(dba)_2$	P^tBu_2Me	8	32	49	11
3	$Pd_2(dba)_3$	P^tBu_2Me	trace	0	32	66
4	Cp(cinnamyl)Pd	P^tBu_2Me	18	trace	9	62
5	[Pd(1-^{t}Bu-indenyl)Cl]$_2$	P^tBu_2Me	6	trace	13	70
6	$PdCl_2$	P^tBu_2Me	0	0	0	94
7	[Cl(cinnamyl)Pd]$_2$	P^tBu_2Me	0	0	0	83
8	Cp(allyl)Pd	None	0	0	0	99
9[b]	Cp(allyl)Pd	P^tBu_2Me	29	6	60	0
10[b]	Cp(allyl)Pd	P^nBu_3	trace	2	76	0
11[b]	Cp(allyl)Pd	P^tBu_3	0	0	trace	98
12[b]	Cp(allyl)Pd	PCy_3	0	0	96	0
13[b]	Cp(allyl)Pd	$PCyp_3$	0	0	93	0
14[b]	Cp(allyl)Pd	PMe_2Ph	0	0	65	0
15[b]	Cp(allyl)Pd	PMe_3	0	0	66	0
16[b]	Cp(allyl)Pd	$P(NMe_2)_3$	0	0	trace	91
17[b]	Cp(allyl)Pd	D^{t}BPF	trace	16	43	0
18[b]	Cp(allyl)Pd	DPEphos	trace	0	44	0

[a] ^{1}H NMR yields. [b] The reaction was conducted at 70 °C.

structure of Pd catalysts and ligands

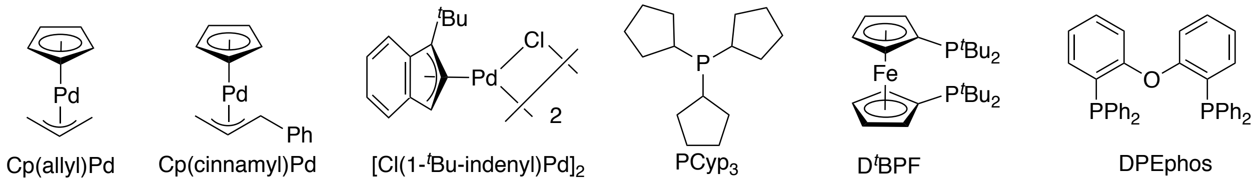

1-2. Effect of solvent

A typical procedure. In a glove box, to a 3 mL vial (vial **A**) with a magnetic stir (10 mm) bar, were added Cp(allyl)Pd (0.85 mg, 4 μmol, 2 mol%), P^tBu_2Me (1.3 mg, 8 μmol, 4 mol%) and solvent (100 μL). The resulting solution was stirred at 60 °C for 10 min on an aluminum heating block. In another vial (3 mL) (vial **B**), aziridine **1a** (54.6 mg, 0.20 mmol), (pin)B–$SiMe_2Ph$ **2** (62.8 mg, 0.24 mmol, 1.2 equiv), 2,2'-bipyridine (6.2 mg, 40 μmol, 20 mol%), and solvent (500 μL) were added. After allowing the vial **A** cool to room temperature, the content of vial **A** was transferred to vial **B**, and the vial **B** was capped with a hole cap and a Teflon®/rubber septum and removed from the glove box. Deionized H_2O (100 μL) was

added through the septum under a stream of N_2 gas, and the resulting mixture was stirred at 80 °C on an aluminum heating block for 3 h. The reaction mixture was filtrated through the Celite pad (2.0 cm height), and the residue was washed with CH_2Cl_2 (10 mL × 3). The filtrate was dried by evaporating the solvents under vacuum. Yields of products were estimated based on ^{1}H NMR integration of the crude product using 1,1,2,2-tetrachloroethane as an internal standard.

Table S2. Effect of Solvent

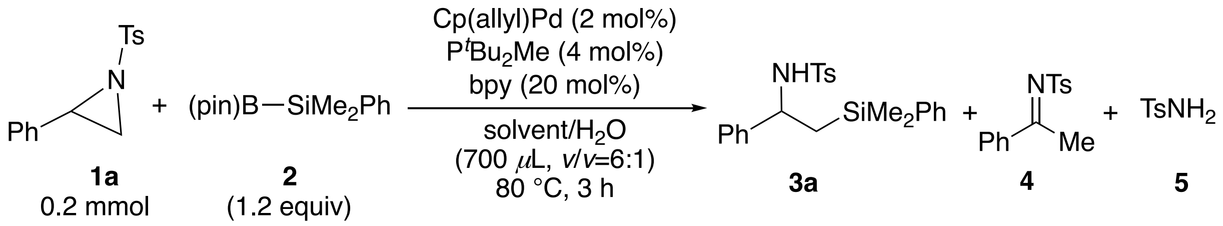

entry	solvent	yield (%)[a] 3a	4	5	recovery (%)[a]
1	MTBE	12	1	29	49
2	toluene	trace	3	50	32
3[b]	1,4-dioxane	0	0	29	0
4	MeCN	0	0	99	0
5	THF	0	0	99	0
6	DMF	0	11	54	0
7	DMA	0	0	50	0
8	CPME	29	6	60	0

[a] ^{1}H NMR yields. [b] Ph⁀NHTs : 63%

1-3. Effect of additive

A typical procedure. In a glove box, to a 3 mL vial (vial **A**) with a magnetic stir (10 mm) bar, were added Cp(allyl)Pd (0.85 mg, 4 μmol, 2 mol%), P^tBu_2Me (1.3 mg, 8 μmol, 4 mol%), and CPME (100 μL). The resulting solution was stirred at 60 °C for 10 min on an aluminum heating block. In another vial (vial **B**), aziridine **1a** (54.6 mg, 0.20 mmol), (pin)B–$SiMe_2Ph$ **2** (62.8 mg, 0.24 mmol, 1.2 equiv), additive (20 mol%), and CPME (500 μL) were added. After allowing the vial **A** cool to room temperature, the content of vial **A** was transferred to vial **B**, and the vial **B** was capped with a hole cap and a Teflon®/rubber septum and removed from the glove box. Deionized H_2O (100 μL) was added through the septum under a stream of N_2 gas, and the resulting mixture was stirred at 80 °C on an aluminum heating block for 3 h. The reaction mixture was filtrated through the Celite pad (2.0 cm height), and the residue was washed with CH_2Cl_2 (10 mL × 3). The filtrate was dried by evaporating the solvents under vacuum. Yields of products were estimated based on ^{1}H NMR integration of the crude product using 1,1,2,2-tetrachloroethane as an internal standard.

Table S3. Effect of additive

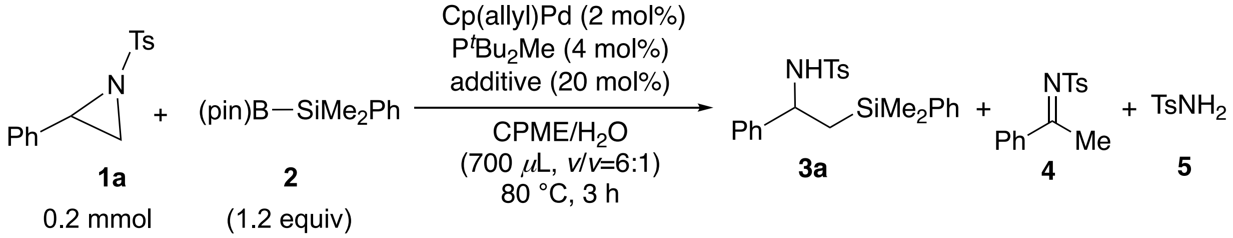

entry	additive	yield (%)[a]			recovery (%)[a]
		**3a**	**4**	**5**	
1[b]	None	5	39	43	0
2	bipyridine	26	15	58	0
3	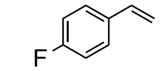	7	32	40	0
4	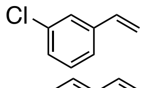	4	37	37	0
5	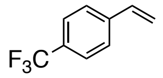	5	35	45	0
6	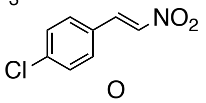	trace	0	15	71
7	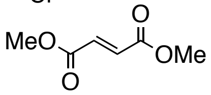	0	0	0	100
8	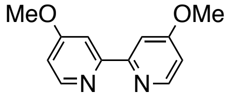	18	0	44	37
9	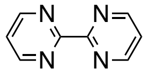	16	35	41	trace
10	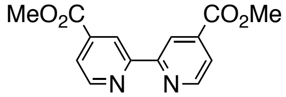	3	5	82	0
11	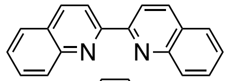	4	22	38	20
12	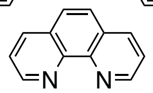	2	34	42	0
13	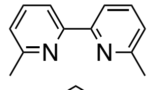	trace	0	11	75
14	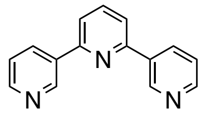	trace	23	23	40
15	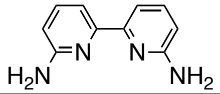	0	0	trace	84

[a] ^1H NMR yields. [b] 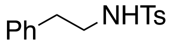 : 4%

1-4. Effect of Cu catalyst

A typical procedure. In a glove box, to a 3 mL vial (vial **A**) with a magnetic stir (10 mm) bar were added Cp(allyl)Pd (1.7 mg, 8 μmol, 4 mol%), P^tBu_2Me (1.3 mg, 8 μmol, 4 mol%), and CPME (100 μL). The resulting solution was stirred at 60 °C for 10 min on an aluminum heating block. In another vial (vial **B**),

aziridine **1a** (54.6 mg, 0.20 mmol), (pin)B–$SiMe_2Ph$ **2** (62.8 mg, 0.24 mmol, 1.2 equiv), 2,2'-bipyridine (3.1 mg, 20 μmol, 10 mol%), copper salt (4 mol%), ^{i}PrOH (100 μL), and CPME (800 μL) were added. After allowing the vial **A** cool to room temperature, the content of vial **A** was transferred to vial **B**, and the vial **B** was capped with a hole cap and a Teflon®/rubber septum and removed from the glove box. The resulting mixture was stirred at 40 °C on an aluminum heating block for 8 h. The reaction mixture was filtrated through the Celite pad (2.0 cm height), and the residue was washed with CH_2Cl_2 (10 mL × 3). The filtrate was dried by evaporating the solvents under vacuum. Yields of products were estimated based on ^{1}H NMR integration of the crude product using 1,1,2,2-tetrachloroethane as an internal standard.

Table S4. Effect of copper catalyst

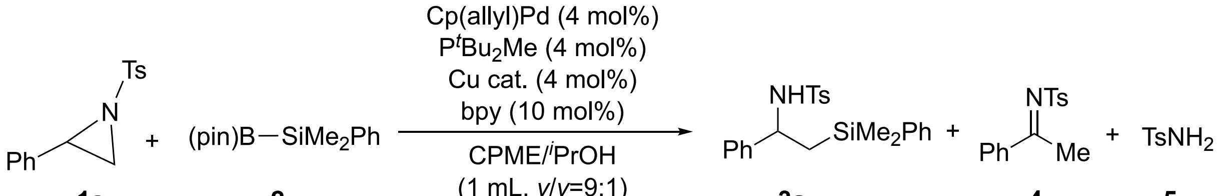

1a 0.2 mmol; **2** (1.2 equiv); **3a**; **4**; **5**

entry	Cu cat.	yield (%)^a 3a	4	5	recovery (%)^a
1	CuCl	35	0	4	55
2	CuSCN	21	trace	7	63
3	$CuCl_2$	4	0	2	82
4	CuF_2	83	0	5	0
5	$Cu(OH)_2$	83	0	7	0
6	$CuSO_4\cdot5H_2O$	81	trace	14	0
7	$CuSO_4$	74	0	11	0
8	$Cu(acac)_2$	19	9	2	60
9	$Cu(CF_3acac)_2$	0	0	0	97
10	$Cu(OAc)_2$	13	trace	20	45

^{a} ^{1}H NMR yields.

1-5. Effect of temperature

A typical procedure. In a glove box, to a 3 mL vial (vial **A**) with a magnetic stir (10 mm) bar, were added Cp(allyl)Pd (1.7 mg, 8 μmol, 4 mol%), P^tBu_2Me (1.3 mg, 8 μmol, 4 mol%), and CPME (100 μL). The resulting solution was stirred at 60 °C for 10 min on an aluminum heating block. In another vial (vial **B**), aziridine **1a** (54.6 mg, 0.20 mmol), (pin)B–$SiMe_2Ph$ **2** (62.8 mg, 0.24 mmol, 1.2 equiv), 2,2'-bipyridine (3.1 mg, 20 μmol, 10 mol%), CuF_2 (0.8 mg, 8 μmol, 4 mol%), ^{i}PrOH (100 μL), and CPME (800 μL) were added. After allowing the vial **A** cool to room temperature, the content of vial **A** was transferred to vial **B**, and the vial **B** was capped with a hole cap and a Teflon®/rubber septum and removed from the glove box. The resulting mixture was stirred at a temperature indicated in the table on an aluminum heating block for 8 h. The reaction mixture was filtrated through the Celite pad (2.0 cm height), and the residue was washed with CH_2Cl_2 (10 mL × 3). The filtrate was dried by evaporating the solvents under vacuum. Yields of products were estimated based on ^{1}H NMR integration of the crude product using 1,1,2,2-tetrachloroethane as an internal standard.

Table S5. Effect of temperature

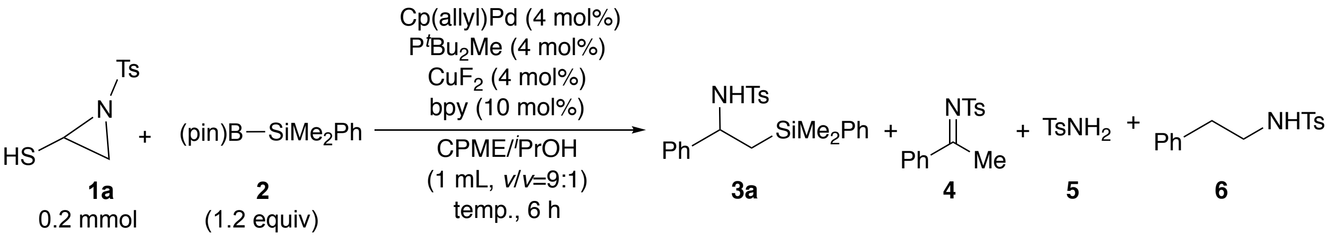

entry	temp. (°C)	yield (%)[a]				recovery (%)[a]
		**3a**	**4**	**5**	**6**	
1	rt. (23)	66	0	4	0	24
2	35	90	0	4	0	0
3	40	83	0	4	0	0
4	60	85	0	5	trace	0
5	80	68	15	4	5	0

[a] ^{1}H NMR yields.

1-6. Effect of proton source

A typical procedure. In a glove box, to a 3 mL vial (vial **A**) with a magnetic stir (10 mm) bar were added Cp(allyl)Pd (1.7 mg, 8 μmol, 4 mol%), P^{t}Bu$_2$Me (1.3 mg, 8 μmol, 4 mol%) and CPME (100 μL), and the resulting solution was stirred at 60 °C for 10 min on an aluminum heating block. In another vial (vial **B**), aziridine **1a** (54.6 mg, 0.20 mmol), (pin)B–SiMe$_2$Ph **2** (62.8 mg, 0.24 mmol, 1.2 equiv), 2,2'-bipyridine (3.1 mg, 20 μmol, 10 mol%), $CuSO_4$ (1.3 mg, 8 μmol, 4 mol%), proton source (100 μL) and CPME (800 μL) were added. After allowing the vial **A** cool to room temperature, the content of vial **A** was transferred to vial **B**, and the vial **B** was capped with a hole cap and a Teflon®/rubber septum and removed from the glove box. The resulting mixture was stirred at 40 °C on an aluminum heating block for 8 h. The reaction mixture was filtrated through the Celite pad (2.0 cm), and the residue was washed with CH_2Cl_2 (10 mL × 3). The filtrate was dried by evaporating the solvents under vacuum. Yields of products were estimated based on ^{1}H NMR integration of the crude product using 1,1,2,2,-tetrachloroethane as an internal standard.

Table S6. Effect of proton source

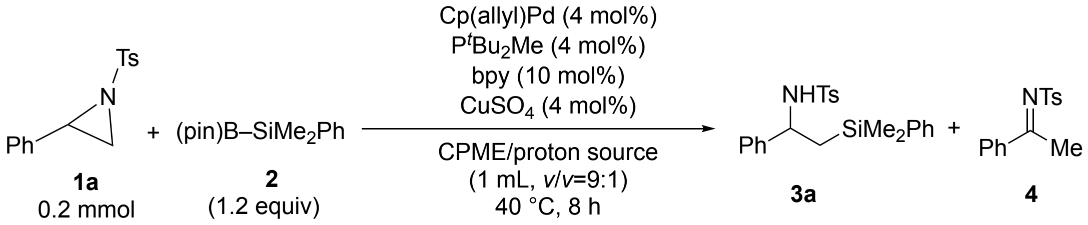

entry	proton source	yield (%)[a]		recovery (%)[a]
		**3a**	**4**	
1	H_2O	75	4	8
2	MeOH	89	6	0
3	^{i}PrOH	74	11	0
4	^{t}BuOH	trace	28	66

[a] ^{1}H NMR yields.

1-7. Effect of amount of MeOH

A typical procedure. In a glove box, to a 3 mL vial (vial **A**) with a magnetic stir (10 mm) bar, were added Cp(allyl)Pd (1.7 mg, 8 μmol, 4 mol%), $P^{t}Bu_2Me$ (1.3 mg, 8 μmol, 4 mol%), and CPME (100 μL). The resulting solution was stirred at 60 °C for 10 min on an aluminum heating block. In another vial (vial **B**), aziridine **1a** (54.6 mg, 0.20 mmol), (pin)B–$SiMe_2Ph$ **2** (62.8 mg, 0.24 mmol, 1.2 equiv), 2,2'-bipyridine (3.1 mg, 20 μmol, 10 mol%), $CuSO_4$ (1.3 mg, 8 μmol, 4 mol%), MeOH (0–100 equiv), and CPME (800 μL) were added. After allowing the vial **A** cool to room temperature, the content of vial **A** was transferred to vial **B**, and the vial **B** was capped with a hole cap and a Teflon®/rubber septum and removed from the glove box. The resulting mixture was stirred at 35 °C on an aluminum heating block for 8 h. The reaction mixture was filtrated through the Celite pad (2.0 cm), and the residue was washed with CH_2Cl_2 (10 mL × 3). The filtrate was dried by evaporating the solvents under vacuum. Yields of products were estimated based on ^{1}H NMR integration of the crude product using 1,1,2,2-tetrachloroethane as an internal standard.

Table S7. Effect of amount of MeOH

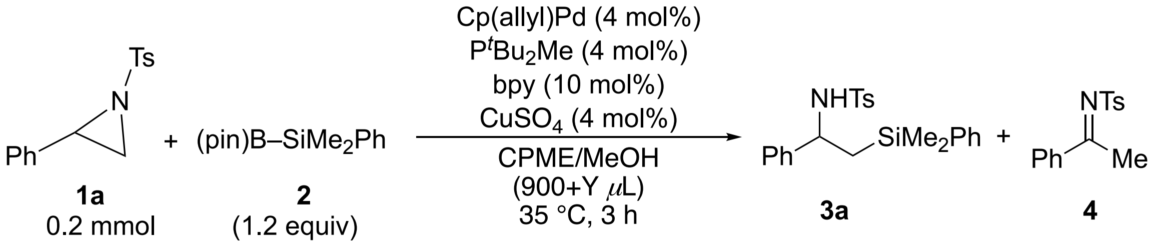

entry	MeOH (X eq., Y μL)	yield (%)[a] 3a	yield (%)[a] 4	recovery (%)[a]
1	0 eq., 0 μL	trace	0	93
2	1 eq., 8 μL	36	5	43
3	2 eq., 16 μL	62	9	20
4	5 eq., 40 μL	85	8	trace
5	100 eq., 800 μL	15	15	39

[a] ^{1}H NMR yields.

1-8. Effect of amount of bpy

A typical procedure. In a glove box, to a 3 mL vial (vial **A**) with a magnetic stir (10 mm) bar, were added Cp(allyl)Pd (1.7 mg, 8 μmol, 4 mol%), $P^{t}Bu_2Me$ (1.3 mg, 8 μmol, 4 mol%), and CPME (100 μL). The resulting solution was stirred at 60 °C for 10 min on an aluminum heating block. In another vial (vial **B**), aziridine **1a** (54.6 mg, 0.20 mmol), (pin)B–$SiMe_2Ph$ **2** (62.8 mg, 0.24 mmol, 1.2 equiv), 2,2'-bipyridine (0–100 mol%), $CuSO_4$ (1.3 mg, 8 μmol, 4 mol%), MeOH (100 μL), and CPME (800 μL) were added. After allowing the vial **A** cool to room temperature, the content of vial **A** was transferred to vial **B**, and the vial **B** was capped with a hole cap and a Teflon®/rubber septum and removed from the glove box. The resulting mixture was stirred at room temperature for 4 h. The reaction mixture was filtrated through the Celite pad (2.0 cm height), and the residue was washed with CH_2Cl_2 (10 mL × 3). The filtrate was dried by evaporating the solvents under vacuum. Yields of products were estimated based on ^{1}H NMR integration of the crude product using 1,1,2,2-tetrachloroethane as an internal standard.

Table S8. Effect of amount of bpy

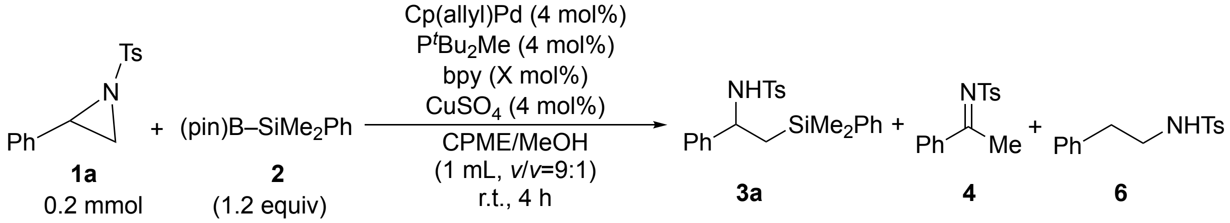

entry	bpy (X mol%)	yield (%)[a]			recovery (%)[a]
		**3a**	**4**	**6**	
1[b]	0	0	24	13	14
2	10	81	5	0	trace
3	20	73	9	0	trace
4	50	60	13	0	13
5	100	50	16	0	24

[a] ^{1}H NMR yields. [b] 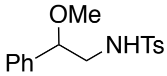

: 53%

2. Ring-opening C(sp^3)–Si cross-coupling and tandem reaction at the 2-position.

2-1. Effect of Pd catalyst

A typical procedure. In a glove box, to a 3 mL vial with a magnetic stir (10 mm) bar, were added aziridine **1a** (54.6 mg, 0.20 mmol), 2,2'-bipyridine (3.1 mg, 20 μmol, 10 mol%), $CuSO_4$ (1.3 mg, 8 μmol, 4 mol%), Pd catalyst (4 mol%), IPr (1.5 mg, 4 μmol, 2 mol%), CPME (900 μL), (pin)B–$SiMe_2Ph$ (62.8 mg, 0.24 mmol, 1.2 equiv) and MeOH (100 μL) were added. The vial was capped with a hole cap and a Teflon®/rubber septum and removed from the glove box. The resulting mixture was stirred at 60 °C on an aluminum heating block for 3 h. The reaction mixture was filtrated through the Celite pad (2.0 cm height), and the residue was washed with CH_2Cl_2 (10 mL × 3). The filtrate was dried by evaporating the solvents under vacuum. Yields of products were estimated based on ^{1}H NMR integration of the crude product using 1,1,2,2-tetrachloroethane as an internal standard.

Table S9. Effect of Pd catalyst

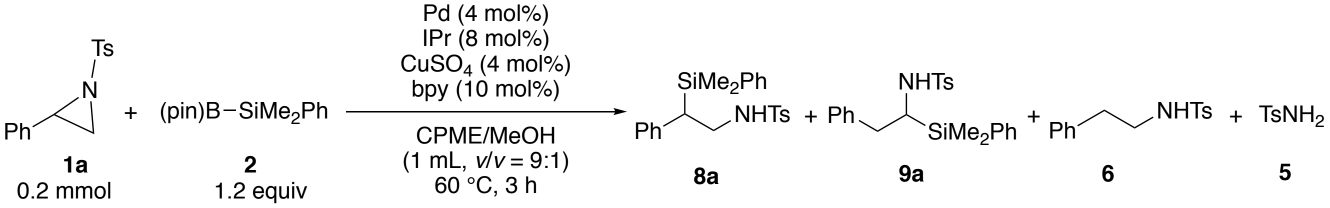

entry	Pd	yield (%)[a]				recovery (%)[a]
		**8a**	**9a**	**6**	**5**	
1	Cp(allyl)Pd	0	5	40	0	10
2	Pd_2dba_3	0	0	46	0	17
3	$Pd(PPh_3)_4$	0	41	0	10	49
4	Pd(P(*o*-tolyl)$_3$)$_2$	0	31	35	0	4
5[b]	IPr–Pd–PPh_3	3	9	0	0	0

[a] ^{1}H NMR yields. [b] without of IPr, 50 °C.

2-2. Effect of additive

A typical procedure. In a glove box, to a 3 mL vial with a magnetic stir (10 mm) bar, were added aziridine **1a** (54.6 mg, 0.20 mmol), additive (10 mol%), CuF_2 (0.8 mg, 8 µmol, 4 mol%), $Pd(PPh_3)_4$ (4.6 mg, 4 µmol, 2 mol%), IPr (1.5 mg, 4 µmol, 2 mol%), solvent (900 µL), (pin)B–$SiMe_2Ph$ **2** (62.8 mg, 0.24 mmol, 1.2 equiv), and MeOH (100 µL) were added. The vial was capped with a hole cap and a Teflon®/rubber septum and removed from the glove box. The resulting mixture was stirred at temperature on an aluminum heating block for 3 h. The reaction mixture was filtrated through the Celite pad (2.0 cm height), and the residue was washed with CH_2Cl_2 (10 mL × 3). The filtrate was dried by evaporating the solvents under vacuum. Yields of products were estimated based on ^1H NMR integration of the crude product using 1,1,2,2-tetrachloroethane as an internal standard.

Table S10. Effect of additive

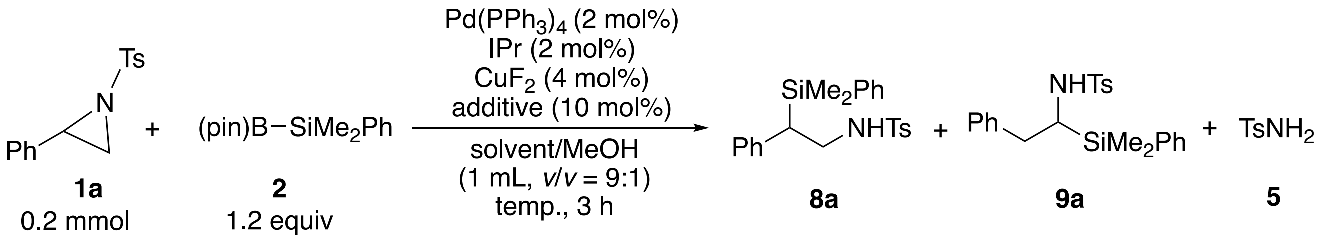

entry	additive	temp.	solvent	yield (%)[a] 8a	9a	5	recovery (%)[a]
1	bpy	r.t.	CPME	0	24	66	2
2	bpy	40 °C	CPME	0	38	48	7
3	bpy	50 °C	CPME	0	74	5	15
4	bpy	50 °C	toluene	0	78	6	0
5	phen	50 °C	toluene	30	57	5	0

[a] ^1H NMR yields.

2-3. Effect of Cu catalyst

A typical procedure. In a glove box, to a 3 mL vial with a magnetic stir (10 mm) bar, were added aziridine **1a** (54.6 mg, 0.20 mmol), 1,10'-phenanthroline (3.6 mg, 20 µmol, 10 mol%), Cu catalysts (4 mol%), IPr–Pd–PPh_3 (6.0 mg, 8 µmol, 4 mol%), CPME (900 µL), (pin)B–$SiMe_2Ph$ **2** (62.8 mg, 0.24 mmol, 1.2 equiv) and MeOH (100 µL) were added. The vial was capped with a hole cap and a Teflon®/rubber septum and removed from the glove box. The resulting mixture was stirred at 50 °C on an aluminum heating block for 12 h. The reaction mixture was filtrated through the Celite pad (2.0 cm height), and the residue was washed with CH_2Cl_2 (10 mL × 3). The filtrate was dried by evaporating the solvents under vacuum. Yields of products were estimated based on ^1H NMR integration of the crude product using 1,1,2,2-tetrachloroethane as an internal standard.

Table S11. Effect of Cu catalysts

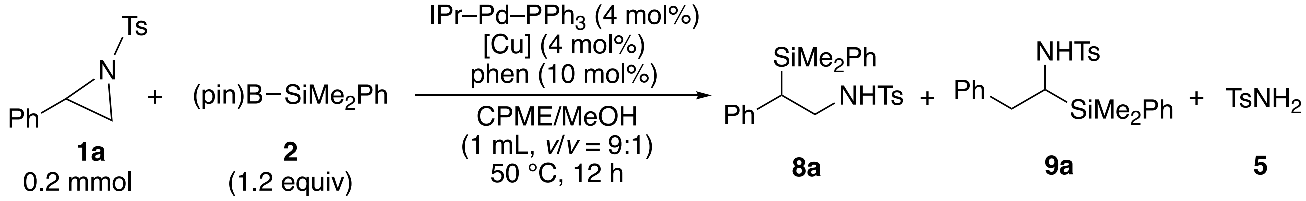

entry	[Cu]	yield (%)[a]			recovery (%)[a]
		**8a**	**9a**	**5**	
1	CuCl	55	14	16	0
2	$CuCl_2$	31	30	22	0
3	$Cu(OH)_2$	20	13	70	0
4[b, c]	CuF_2	55	18	0	10
5[b]	$CuSO_4$	3	9	0	0
6[c, d]	IPr–Cu–Cl	0	20	13	20

[a] ^{1}H NMR yields. [b] bpy instead of phen. [c] 3 h. [d] without of phen

2-4. Effect of NHC ligand

A typical procedure. In a glove box, to a 3 mL vial with a magnetic stir (10 mm) bar, were added aziridine **1a** (54.6 mg, 0.20 mmol), 1,10'-phenanthroline (3.6 mg, 20 μmol, 10 mol%), CuF_2 (0.8 mg, 8 μmol, 4 mol%), NHC–Pd–PPh_3 (2 mol%), CPME (900 μL), (pin)B–$SiMe_2Ph$ **2** (62.8 mg, 0.24 mmol, 1.2 equiv), and MeOH (100 μL) were added. The vial was capped with a hole cap and a Teflon®/rubber septum and removed from the glove box. The resulting mixture was stirred at 50 °C on an aluminum heating block for 3 h. The reaction mixture was filtrated through the Celite pad (2.0 cm height), and the residue was washed with CH_2Cl_2 (10 mL × 3). The filtrate was dried by evaporating the solvents under vacuum. Yields of products were estimated based on ^{1}H NMR integration of the crude product using 1,1,2,2-tetrachloroethane as an internal standard.

Table S12. Effect of NHC

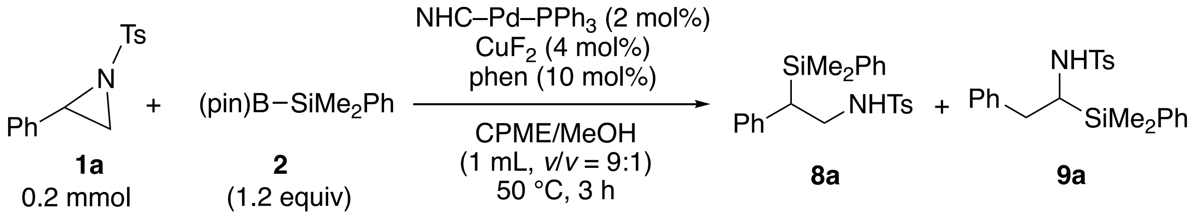

entry	NHC	yield (%)[a]		recovery (%)[a]
		**8a**	**9a**	
1	SIPr	88	11	0
2	IPr	85	12	0
3	^{Me}IPr	79	18	0

[a] ^{1}H NMR yields.

2-5. Effect of solvent

A typical procedure. In a glove box, to a 3 mL vial with a magnetic stir (10 mm) bar were added aziridine **1a** (54.6 mg, 0.20 mmol), 1,10'-phenanthroline (3.6 mg, 20 μmol, 10 mol%), CuF_2 (0.8 mg, 8 μmol, 4 mol%), SIPr–Pd–PPh_3 (3.0 mg, 4 μmol, 2 mol%), solvent (900 μL), (pin)B–$SiMe_2Ph$ **2** (62.8 mg, 0.24 mmol, 1.2 equiv), and MeOH (100 μL) were added. The vial was capped with a hole cap and a Teflon®/rubber septum and removed from the glove box. The resulting mixture was stirred at 50 °C on an aluminum heating block for 8 h. The reaction mixture was filtrated through the Celite pad (2.0 cm height), and the residue was washed with CH_2Cl_2 (10 mL × 3). The filtrate was dried by evaporating the solvents under vacuum. Yields of products were estimated based on ^{1}H NMR integration of the crude product using 1,1,2,2-tetrachloroethane as an internal standard.

Table S13. Effect of solvent

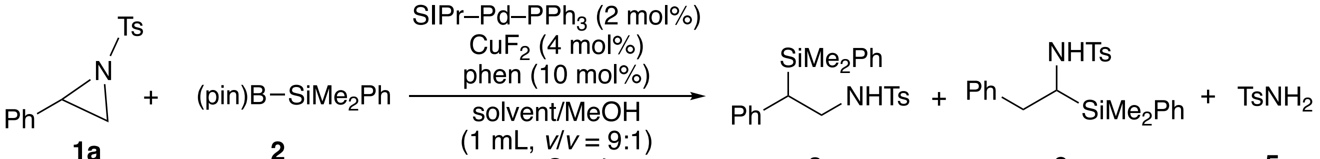

1a 0.2 mmol; **2** 1.2 equiv; **8a**; **9a**; **5**

entry	solvent	yield (%)^a 8a	9a	5	recovery (%)^a
cf.	CPME	88	12	0	0
1	toluene	77	26	0	0
2	acetone	64	22	6	6
3	1,4-dioxane	88	5	0	0
4	MTHP	86	10	0	0
5	MTBE	65	17	4	0
6	MeOH	0	44	21	0
7	THF	80	12	3	0
8	DMF	29	16	24	10
9	DMA	58	16	9	0

^{a} ^{1}H NMR yields.

2-6. Effect of amount of MeOH

A typical procedure. In a glove box, to a 3 mL vial with a magnetic stir (10 mm) bar were added aziridine **1a** (54.6 mg, 0.20 mmol), 1,10'-phenanthroline (3.6 mg, 20 μmol, 10 mol%), CuF_2 (0.8 mg, 8 μmol, 4 mol%), SIPr–Pd–PPh_3 (3.0 mg, 4 μmol, 2 mol%), CPME (900 μL), (pin)B–$SiMe_2Ph$ **2** (62.8 mg, 0.24 mmol, 1.2 equiv), and MeOH (3–25 equiv) were added. The vial was capped with a hole cap and a Teflon®/rubber septum and removed from the glove box. The resulting mixture was stirred at 50 °C on an aluminum heating block for 3 h. The reaction mixture was filtrated through the Celite pad (2.0 cm height), and the residue was washed with CH_2Cl_2 (10 mL × 3). The filtrate was dried by evaporating the

solvents under vacuum. Yields of products were estimated based on ^{1}H NMR integration of the crude product using 1,1,2,2-tetrachloroethane as an internal standard.

Table S14. Effect of amount of MeOH

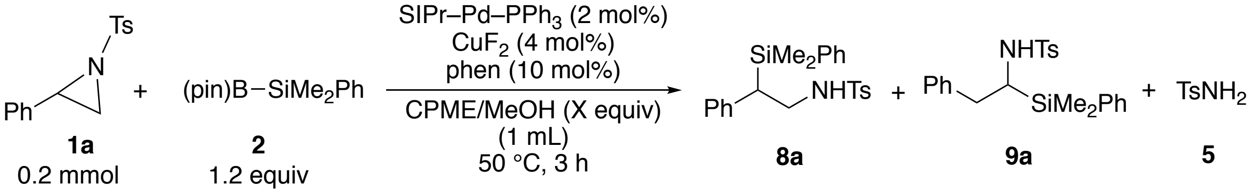

entry	X equiv (v μL)	yield (%)[a] 8a	9a	5	recovery (%)[a]
1	3.0 (24 μL)	89	2	0	6
2	6.0 (50 μL)	86	7	6	0
3	12.0 (100 μL)	88	12	0	0
4	25.0 (200 μL)	65	28	0	0

[a] ^{1}H NMR yields.

2-7. Effect of the oxidation state of Cu catalyst

A typical procedure. In a glove box, to a 3 mL vial with a magnetic stir (10 mm) bar were added aziridine **1a** (54.6 mg, 0.20 mmol), 1,10'-phenanthroline (2.2 mg, 12 μmol, 6 mol%), copper complex (8 μmol, 4 mol%), SIPr–Pd–PPh$_3$ (3.0 mg, 4 μmol, 2 mol%), CPME (900 μL), (pin)B–SiMe$_2$Ph **2** (62.8 mg, 0.24 mmol, 1.2 equiv), and MeOH (100 μL) were added. The vial was capped with a hole cap and a Teflon®/rubber septum and removed from the glove box. The resulting mixture was stirred at 50 °C on an aluminum heating block for 3 h. The reaction mixture was filtrated through the Celite pad (2.0 cm height), and the residue was washed with CH_2Cl_2 (10 mL × 3). The filtrate was dried by evaporating the solvents under vacuum. Yields of products were estimated based on ^{1}H NMR integration of the crude product using 1,1,2,2-tetrachloroethane as an internal standard.

Table S15. Effect of the oxidation state of Cu catalyst

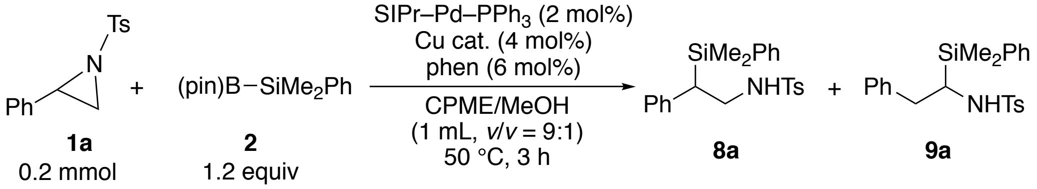

entry	Cu cat.	phen	yield (%)[a] 8a	9a	recovery (%)[a]
1	(phen)CuF	–	31	23	44
2	(phen)CuF	O	35	16	44
3	(phen)CuF_2	–	42	17	34
4	(phen)CuF_2	O	58	9	30

[a] ^{1}H NMR yields.

2-8. Effect of amount of PPh_3

A typical procedure. In a glove box, to a 3 mL vial with a magnetic stir (10 mm) bar were added aziridine **1a** (54.6 mg, 0.20 mmol), 2,2'-bipyridine (3.1 mg, 20 μmol, 10 mol%), CuF_2 (0.8 mg, 8 μmol, 4 mol%), SIPr–Pd–PPh_3 (3.0 mg, 4 μmol, 2 mol%), PPh_3 (0, 2, and 4 mol%), toluene (900 μL), (pin)B–$SiMe_2Ph$ **2** (62.8 mg, 0.24 mmol, 1.2 equiv), and MeOH (100 μL) were added. The vial was capped with a hole cap and a Teflon®/rubber septum and removed from the glove box. The resulting mixture was stirred at 50 °C on an aluminum heating block for 8 h. The reaction mixture was filtrated through the Celite pad (2.0 cm height), and the residue was washed with CH_2Cl_2 (10 mL × 3). The filtrate was dried by evaporating the solvents under vacuum. Yields of products were estimated based on ^1H NMR integration of the crude product using 1,1,2,2-tetrachloroethane as an internal standard.

Table S16. Effect of amount of PPh_3

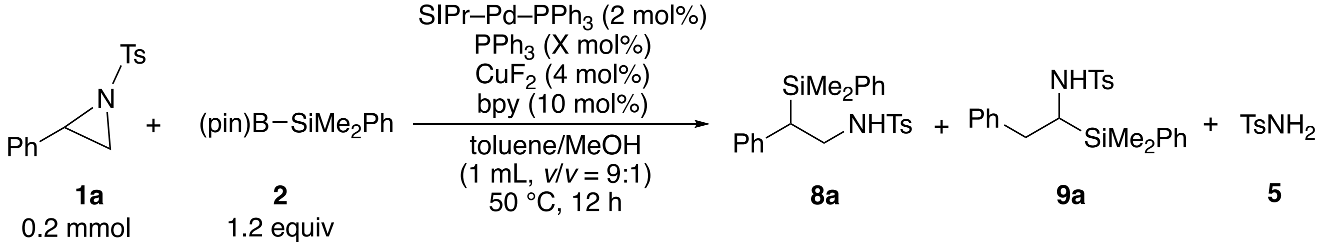

**1a**	**2**		**8a**	**9a**	**5**
0.2 mmol	1.2 equiv				

entry	PPh_3 (X mol%)	yield (%)[a] **8a**	**9a**	**5**	recovery (%)[a]
1[b]	0 mol%	50	16	0	21
2[c]	2 mol%	0	88	0	0
3[c]	4 mol%	0	100	0	0

[a] ^1H NMR yields. [b] 3 h. [c] 0.5 mmol scale.

EPR experiments

EPR measurements were carried out on a BRUKER EMX-micro. In a typical experiment, a solution of a Cu complex (i.e., Cu^{II}(phen)F_2, Cu^{I}(phen)F of 4 mol%) in CPME/MeOH (1 mL, 1.0 mM) with suitable reagents/catalysts (silylaboran **2**, SIPr-Pd-PPh_3, aziridine **1a**) was introduced into a vial and the resulting solution was heated at 50 °C for 20 min. A portion of the solution was injected into an EPR tube inside a glove box and sealed with a silicon rubber cap. The EPR tube was placed in an acetone/liquid nitrogen bath at ca. –90 °C. The outside of the EPR tube was quickly wiped with a tissue paper, tube was dipped in a liquid-N2 bath, and EPR spectrum was measured at 116 K with X-band microwave (9.385 GHz, 1.0 mW).

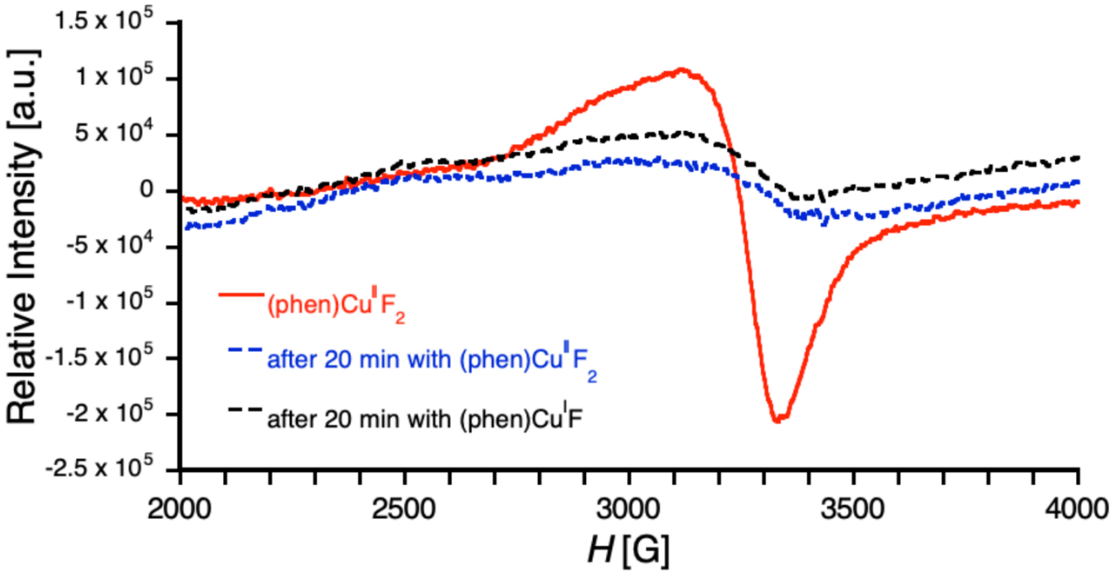

Fig. S1 EPR spectra of the reaction mixtures. Solid red line indicates the signal of the solution containing **1a**, **2**, (phen)$Cu^{II}F_2$ (cat.), SIPr-Pd-PPh_3 (cat.) in CMPE/MeOH without heating; dashed blue line indicates the signal of the solution containing **1a**, **2**, (phen)Cu^{II}F (cat.), SIPr-Pd-PPh_3 (cat.) in CMPE/MeOH after heating at 50 °C for 20 min; dashed black line indicates the signal of the solution containing **1a**, **2**, (phen)Cu^{I}F (cat.), SIPr-Pd-PPh_3 (cat.) in CMPE/MeOH after heating at 50 °C for 20 min.

Spectroscopic data of products

A typical procedure for the C3-selective ring-opening C(sp^3)–Si cross-coupling. In a glove box, to a 3 mL vial (vial **A**) with a magnetic stir (10 mm) bar, were added Cp(allyl)Pd (4.3 mg, 20 µmol, 4 mol%), P^tBu_2Me (3.2 mg, 20 µmol, 4 mol%), and CPME (200 µL). The resulting solution was stirred at 60 °C for 10 min on an aluminum heating block. In another vial (vial **B**), aziridine **1** (0.50 mmol), (pin)B–$SiMe_2Ph$ **2** (157.2 mg, 0.60 mmol, 1.2 equiv), 2,2'-bipyridine (7.8 mg, 50 µmol, 10 mol%), $CuSO_4$ (3.2 mg, 20 µmol, 4 mol%), and CPME (2.05 mL) were added. After allowing the vial **A** cool to room temperature, the content of vial **A** was transferred to vial **B**, and MeOH (200 µL) was added to the vial. The vial **B** was capped with a hole cap and a Teflon®/rubber septum and removed from the glove box. The resulting mixture was stirred at a 40 °C on an aluminum heating block for 3 h. The reaction mixture was filtrated through the Celite pad (2.0 cm height), and the residue was washed with CH_2Cl_2 (10 mL ×

3). The filtrate was dried by evaporating the solvents under vacuum. Yields of products were estimated based on ^{1}H NMR integration of the crude product using 1,1,2,2-tetrachloroethane as an internal standard. And the product was isolated by flash silica gel chromatography.

_N_-[(2-(Dimethyl(phenyl)silyl)-1-phenylethyl)]-4-methylbenzenesulfonamide (3a)

[CAS No.205642-05-3]

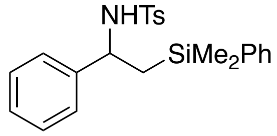

Purified by silica gel column chromatography (SiO_2 silica, *n*-hexane/EtOAc, 10:0 to 8:2). 83% yield; colorless solid; R_f 0.38 (*n*-hexane/EtOAc = 8:2); mp 93.0 °C; ^{1}H NMR (400 MHz, $CDCl_3$): δ 7.40 (2H, d, *J* = 8.4 Hz), 7.36–7.32 (5H, m), 7.11–7.04 (5H, m), 6.90 (2H, d, *J* = 8.4 Hz), 4.60 (1H, d, *J* = 6.4 Hz), 4.35 (1H, ddd, *J* = 9.6, 6.4, 5.6 Hz), 2.34 (3H, s), 1.45 (1H, dd, *J* = 14.4, 5.6 Hz), 1.34 (1H, dd, *J* = 14.4, 9.6 Hz), 0.03 (3H, s), 0.01 (3H, s); ^{13}C NMR (100 MHz, $CDCl_3$): δ 142.7, 141.8, 137.8, 137.5, 133.5, 129.1, 128.3, 127.8, 127.4, 127.0, 126.5, 56.1, 26.4, 21.4, –2.5, –3.4, 1C is missing in the aromatic region, probably due to the overlap of signals in the aromatic region.; ^{29}Si NMR (79 MHz, $CDCl_3$): δ –5.06; IR (ATR, cm^{-1}): 3232, 3066, 3024, 2951, 2885, 1597, 1442, 1323, 1149, 1111, 1087, 1029, 921, 856, 840, 813, 736; MS (EI^+) *m/z* (relative intensity, %): 408 ($[M–H]^+$, 2), 394 ($[M–Me]^+$, 32), 290 ($[M–119]^+$, 100), 228 (46), 135 ($[SiMe_2Ph]^+$, 65); HRMS ($DART^–$): calcd for $C_{23}H_{26}NO_2SSi$ ($[M–H]^+$), 408.1459, found 408.1454.

_N_-(2-(Dimethyl(phenyl)silyl)-1-(_p_-tolyl)ethyl)-4-methylbenzenesulfonamide (3b)

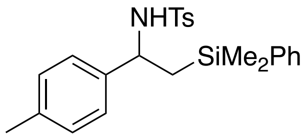

Purified by silica gel column chromatography (SiO_2 silica, *n*-hexane/EtOAc, 10:0 to 8:2). 84% yield; colorless liquid; R_f 0.4 (hexane/EtOAc = 8:2); ^{1}H NMR (400 MHz, $CDCl_3$) δ 7.43 (2H, d, *J* = 7.2 Hz), 7.34–7.29 (5H, m), 7.06 (2H, d, *J* = 8.0 Hz), 6.67 (2H, d, *J* = 7.2 Hz), 7.77 (2H, d, *J* = 8.0 Hz), 4.80 (1H, br), 4.30 (1H, ddd, *J* = 10.4, 6.0, 6.0 Hz) 2.35 (3H, s) 2.24 (3H, s), 1.45 (1H, dd, *J* = 10.4, 6.0 Hz), 1.34 (1H, dd, *J* = 14.4, 10.4 Hz), 0.016 (3H, s), 0.014 (3H, s); ^{13}C NMR (100 MHz, $CDCl_3$): δ 143.1, 136.5, 135.8, 135.1, 135.0, 133.8, 129.4, 129.3, 129.2, 127.6, 127.5, 126.9, 43.3, 36.2, 21.4, 20.8, –4.1, –5.5; ^{29}Si NMR (79 MHz, $CDCl_3$): δ –5.05; IR (ATR, cm^{-1}): 3259, 2953, 1600, 1514, 1427, 1321, 1247, 1153, 1112, 1093, 1022, 943, 918, 812, 761, 731, 700; MS (EI^+) *m/z* (relative intensity, %): 422 ($[M–H]^+$, 2), 408 ($[M–Me]^+$, 8), 290 ($[M–133]^+$, 60), 274 (100), 228 (28), 135 ($[SiMe_2Ph]^+$, 59); HRMS ($DART^–$): calcd for $C_{25}H_{28}NO_2SSi$ ($[M–H]^+$), 422.1615, found 422.1617.

_N_-(2-(Dimethyl(phenyl)silyl)-1-(4-fluorophenyl)ethyl)-4-methylbenzenesulfonamide (3c)

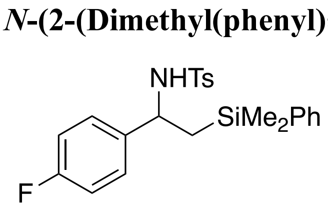

Purified by silica gel column chromatography (SiO_2 silica, *n*-hexane/EtOAc, 10:0 to 8:2). 77% yield; colorless solid; R_f 0.28 (hexane/EtOAc = 8:2); mp 78.0 °C; ^{1}H NMR (400 MHz, $CDCl_3$) δ 7.39–7.30 (7H, m), 7.07 (2H, d, *J* = 7.6 Hz), 6.85 (2H, dd, J_{HH} = 8.8 Hz, J_{HF} = 5.2 Hz), 6.73 (2H, dd, J_{HH} = 8.8 Hz, J_{HF} = 8.8 Hz), 4.81 (1H, br), 4.35 (1H, ddd, *J* = 10.0, 7.2, 6.0 Hz), 2.35 (3H, s), 1.41 (1H, dd, *J* = 14.4, 6.0 Hz), 1.29 (1H, dd, *J* = 14.4, 10.0 Hz), 0.03 (3H, s), 0.02 (3H, s); ^{13}C NMR (100 MHz, $CDCl_3$): δ 161.9 (d, $J_{C–F}$ = 244.5 Hz), 142.8, 137.5, 133.4, 129.6, 129.1, 128.2 (d, $J_{C–F}$ = 8.3 Hz), 127.8, 127.6, 126.9, 126.4, 115.0 (d, $J_{C–F}$ = 21.4 Hz), 55.4, 26.3, 21.3, –2.6, –3.3; ^{29}Si NMR (79 MHz, $CDCl_3$): δ –

5.12; IR (ATR, cm^{-1}): 3242, 3068, 1649, 1604, 1508, 1440, 1325, 1224, 1151, 1111, 1087, 1024, 916, 862, 839, 815, 769, 736, 702; MS (EI^+) *m/z* (relative intensity, %): 426 ($[M–H]^+$, 2), 412 ($[M–Me]^+$, 34), 290 ($[M–137]^+$, 100), 228 (45), 135 ($[SiMe_2Ph]^+$, 68); HRMS ($DART^–$): calcd for $C_{23}H_{25}NO_2FSSi$ ($[M–H]^+$), 426.1364, found 426.1366.

***N*-[(1-(4-Chlorophenyl)-2-(dimethyl(phenyl)silyl)ethyl)]-4-methylbenzenesulfonamide (3d)**

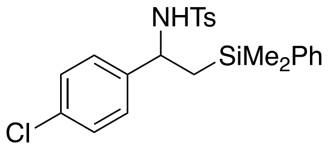

Purified by silica gel column chromatography (SiO_2 silica, *n*-hexane/EtOAc, 10:0 to 8:2). 82% yield; colorless solid; R_f 0.35 (*n*-hexane/EtOAc = 8:2); mp 74.2 °C; ^{1}H NMR (400 MHz, $CDCl_3$): δ 7.38 (2H, d, *J* = 8.0 Hz), 7.36–7.28 (5H, m), 7.04 (2H, d, *J* = 8 Hz), 6.96 (2H, d, *J* = 8.4 Hz), 6.79 (2H, d, *J* = 8.4 Hz), 5.31 (1H, d, *J* = 6.8 Hz), 4.31 (1H, ddd, *J* = 10.0, 6.8, 6.0 Hz), 2.35 (3H, s), 1.38 (1H, dd, *J* = 14.4, 6.0 Hz), 1.34 (1H, dd, *J* = 14.4, 10.0 Hz), 0.03 (3H, s), 0.02 (3H, s); ^{13}C NMR (100 MHz, $CDCl_3$): δ 143.0, 140.3, 137.43, 137.41, 133.4, 133.1, 129.2, 129.1, 128.3, 127.96, 127.93, 126.9, 55.4, 26.3, 21.4, –2.5, –3.2; ^{29}Si NMR (79 MHz, $CDCl_3$): δ –5.10; IR (ATR, cm^{-1}): 3232, 3068, 3043, 2954, 1598, 1490, 1440, 1321, 1246, 1149, 1112, 1087, 1012, 918, 860, 842, 812, 723, 704; MS (EI^+) *m/z* (relative intensity, %): 442 ($[M–H]^+$, 1), 328 ($[M–Me]^+$, 20), 290 ($[M–153]^+$, 100), 228 (44), 135 ($[SiMe_2Ph]^+$, 61); HRMS ($DART^–$): calcd for $C_{23}H_{25}NO_2SSiCl$ ($[M–H]^+$), 442.1069, found 442.1061.

***N*-[(2-(Dimethyl(phenyl)silyl)-1-(4-(trifluoromethyl)phenyl)ethyl)-4-methylbenzenesulfonamide (3e)**

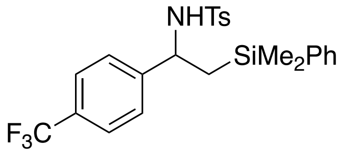

Purified by silica gel column chromatography (SiO_2 silica, *n*-hexane/EtOAc, 10:0 to 8:2). 81% yield; colorless solid; R_f 0.35 (hexane/EtOAc = 8:2); mp 95.1 °C; ^{1}H NMR (400 MHz, $CDCl_3$) δ 7.37–7.21 (9H, m), 6.96–6.94 (4H, m), 5.11 (1H, br), 4.45 (1H, ddd, *J* = 9.2, 6.8, 6.8 Hz), 2.29 (3H, s), 1.39 (1H, dd, *J* = 14.4, 6.8 Hz), 1.28 (1H, dd, *J* = 14.4, 9.2 Hz), 0.10 (3H, s), 0.09 (3H, s); ^{13}C NMR (100 MHz, $CDCl_3$): δ 145.6, 143.0, 137.3, 137.1, 133.3, 129.2 (q, $J_{C–F}$ = 32.1 Hz), 129.1, 129.0, 127.8, 126.96, 126.91, 125.0 (q, $J_{C–F}$ = 3.3 Hz), 123.8 (q, $J_{C–F}$ = 270.9 Hz), 55.6, 26.0, 21.1, –2.8, –3.1; ^{29}Si NMR (79 MHz, $CDCl_3$): δ –5.01; IR (ATR, cm^{-1}): 3272, 3246, 3066, 2964, 2887, 1618, 1597, 1492, 1442, 1325, 1253, 1157, 1124, 1066, 918, 848, 813, 727, 702; MS (EI^+) *m/z* (relative intensity, %): 476 ($[M–H]^+$, 1), 462 ($[M–Me]^+$, 40), 290 ($[M–187]^+$, 100), 228 (46), 135 ($[SiMe_2Ph]^+$, 50); HRMS ($DART^–$): calcd for $C_{24}H_{25}NO_2F_3SSi$ ($[M–H]^+$), 476.1332, found 476.1339.

4-(2-(Dimethyl(phenyl)silyl)-1-((4-methylphenyl)sulfonamido)ethyl)phenyl acetate (3f)

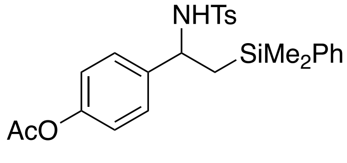

Purified by silica gel column chromatography (SiO_2 silica, *n*-hexane/EtOAc, 10:0 to 8:2). 76% yield; colorless oil; R_f 0.18 (hexane/EtOAc = 8:2); ^{1}H NMR (400 MHz, $CDCl_3$) δ 7.40 (2H, d, *J* = 8.4 Hz), 7.32–7.30 (5H, m), 7.05 (2H, d, *J* = 8.4 Hz), 6.88 (2H, d, *J* = 8.4 Hz), 6.76 (2H, d, *J* = 8.4 Hz), 5.23 (1H, br), 4.36 (1H, ddd, *J* = 9.6, 6.8, 6.4 Hz), 2.32 (3H, s), 2.25 (3H, s), 1.41 (1H, dd, *J* = 14.8, 6.4 Hz), 1.30 (1H, dd, *J* = 14.8, 9.6 Hz), 0.038 (3H, s), 0.033 (3H, s); ^{13}C NMR (100 MHz, $CDCl_3$): δ 168.9, 147.9, 142.8, 139.4, 137.7, 137.3, 133.4, 129.1, 129.0, 127.7, 127.4, 126.8, 121.2, 55.4, 26.2, 21.2, 21.0, –2.6, –3.3; ^{29}Si NMR (79 MHz, $CDCl_3$): δ –5.06; IR (ATR, cm^{-1}): 3275,

3066, 2954, 1762, 1598, 1427, 1323, 1197, 1153, 1112, 1093, 1016, 912, 835, 812, 732, 700; MS (EI^+) *m/z* (relative intensity, %): 466 ($[M–H]^+$, 1), 452 ($[M–Me]^+$, 13), 290 ($[M–177]^+$, 100), 228 (48), 135 ($[SiMe_2Ph]^+$, 93); HRMS ($DART^–$): calcd for $C_{25}H_{28}NO_4SSi$ ($[M–H]^+$), 466.1513, found 466.1510.

Methyl 4-(2-(dimethyl(phenyl)silyl)-1-((4-methylphenyl)sulfonamido)ethyl)benzoate (3g)

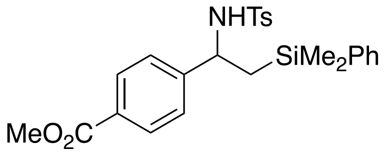

Purified by silica gel column chromatography (SiO_2 silica, *n*-hexane/EtOAc, 10:0 to 8:2). 75% yield; colorless solid; R_f 0.18 (hexane/EtOAc = 8:2); mp 133.6 °C; ^1H NMR (400 MHz, $CDCl_3$) δ 7.71 (2H, d, *J* = 8.8 Hz), 7.38–7.32 (7H, m), 7.02 (2H, d, *J* = 8.0 Hz), 6.95 (2H, d, *J* = 8.0 Hz), 4.97 (1H, br), 4.41 (1H, ddd, *J* = 9.2, 6.4, 6.4 Hz), 3.89 (3H, s), 2.31 (3H, s), 1.40 (1H, dd, *J* = 14.4, 6.4 Hz), 1.30 (1H, dd, *J* = 14.4, 9.2 Hz), 0.04 (3H, s), 0.03 (3H, s); ^{13}C NMR (100 MHz, $CDCl_3$): δ 166.6, 147.0, 143.0, 137.4, 137.3, 133.4, 129.5, 129.2, 129.1, 128.9, 127.8, 126.9, 126.5, 55.7, 52.0, 26.2, 21.3, –2.5, –3.3; ^{29}Si NMR (79 MHz, $CDCl_3$): δ –5.05; IR (ATR, cm^{-1}): 3271, 3066, 2953, 1716, 1610, 1600, 1492, 1433, 1408, 1327, 1278, 1151, 1111, 1014, 920, 813, 738, 702; MS (EI^+) *m/z* (relative intensity, %): 466 ($[M–H]^+$, 1), 452 ($[M–Me]^+$, 33), 290 ($[M–177]^+$, 100), 228 (47), 135 ($[SiMe_2Ph]^+$, 50); HRMS ($DART^–$): calcd for $C_{25}H_{28}NO_4SSi$ ($[M–H]^+$), 466.1513, found 466.1508.

***N*-(1-(2-Chlorophenyl)-2-(dimethyl(phenyl)silyl)ethyl)-4-methylbenzenesulfonamide (3h)**

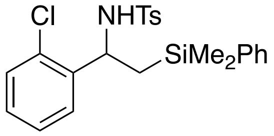

Purified by silica gel column chromatography (SiO_2 silica, *n*-hexane/EtOAc, 10:0 to 8:2). 90% yield; colorless solid; R_f 0.33 (hexane/EtOAc = 8:2); mp 121.0 °C; ^1H NMR (400 MHz, $CDCl_3$) δ 7.43–7.32 (7H, m), 7.06–6.95 (6H, m), 4.97 (1H, br), 4.84–4.78 (1H, m), 2.30 (3H, s), 1.48–1.37 (2H, m), 0.18 (3H, s), 0.13 (3H, s); ^{13}C NMR (100 MHz, $CDCl_3$): δ 142.8, 139.4, 137.8, 137.0, 133.5, 131.7, 129.8, 129.2, 129.0, 128.6, 128.2, 127.9, 127.0, 126.8, 53.8, 24.9, 21.3, –2.8, –3.2; ^{29}Si NMR (79 MHz, $CDCl_3$): δ –4.77; IR (ATR, cm^{-1}): 3282, 3068, 2964, 1597, 1438, 1321, 1305, 1249, 1155, 1116, 1091, 1039, 1020, 910, 835, 813, 723, 702; MS (EI^+) *m/z* (relative intensity, %): 442 ($[M–H]^+$, 1), 428 ($[M–Me]^+$, 33), 290 ($[M–153]^+$, 100), 228 (42), 135 ($[SiMe_2Ph]^+$, 39); HRMS ($DART^–$): calcd for $C_{23}H_{25}NO_2FSSi$ ($[M–H]^+$), 442.1069, found 442.1073.

***N*-(2-(Dimethyl(phenyl)silyl)-1-(*o*-tolyl)ethyl)-4-methylbenzenesulfonamide (3i)**

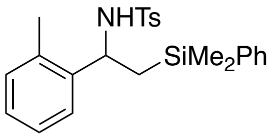

Purified by silica gel column chromatography (SiO_2 silica, *n*-hexane/EtOAc, 10:0 to 8:2). 77% yield; colorless solid; R_f 0.4 (hexane/EtOAc = 8:2); mp 113.0 °C; ^1H NMR (400 MHz, $CDCl_3$) δ 7.43–7.32 (7H, m), 7.06–6.87 (6H, m), 4.71–4.61 (2H, m), 2.32 (3H, s), 1.87 (3H, s), 1.47 (1H, dd, *J* = 14.4, 6.8 Hz), 1.35 (1H, dd, *J* = 14.4, 8.0 Hz), 0.10 (3H, s), 0.04 (3H, s); ^{13}C NMR (100 MHz, $CDCl_3$): δ 142.8, 140.2, 137.9, 137.4, 134.4, 133.5, 130.4, 129.1, 127.8, 127.1, 126.9, 126.2, 125.8, 51.5, 25.7, 21.3, 18.7, –2.3, –3.3; ^{29}Si NMR (79 MHz, $CDCl_3$): δ –5.03; IR (ATR, cm^{-1}): 3284, 3070, 2956, 1598, 1492, 1427, 1317, 1305, 1251, 1151, 1116, 1091, 1016, 914, 812, 771, 723, 702; MS (EI^+) *m/z* (relative intensity, %): 422 ($[M–H]^+$, 1), 408 ($[M–Me]^+$, 26), 290 ($[M–133]^+$, 100), 228 (43), 135 ($[SiMe_2Ph]^+$, 82); HRMS ($DART^–$): calcd for $C_{25}H_{28}NO_2SSi$ ($[M–H]^+$), 422.1615, found 422.1615.

Determination of Stereochemistry in the C3-Selective C(sp^3)–Si Cross-Coupling. To obtain the stereochemical information about the Pd/Cu-cocatalyzed C3-selective C(sp^3)–Si cross-coupling of aziridines, stereo-defined deuterated aziridine *cis*-**1a**-d_1[S14] was cross-coupled with silylborane, which resulted in the cross-coupled product in a regioselective and stereospecific manner to give **1a**-d_1 as the single stereoisomer (Scheme S1). Nevertheless, the moderate coupling constant value (^3J (H^a-H^b) = 5.6 Hz) does not allow us to conclude its stereochemistry. Therefore, we transformed the product into aziridine through stereochemically-reliable sequential two reactions: the Fleming oxidation (stereo-retention process) and intramolecular S_N2-type cyclization (stereo-inversion process) (Scheme 1). In both reactions, the products were obtained as the single stereoisomer, and it turned that the final aziridine product has the *cis*-configuration (i.e., *cis*-**1a**-d_1). From these results, we concluded that the first step (cross-coupling) should proceed with stereo-inversion.

Scheme S1. Cross-coupling of *cis*-1a-d_1 and its derivatization for the determination of stereochemistry of the C(sp^3)–Si coupling.

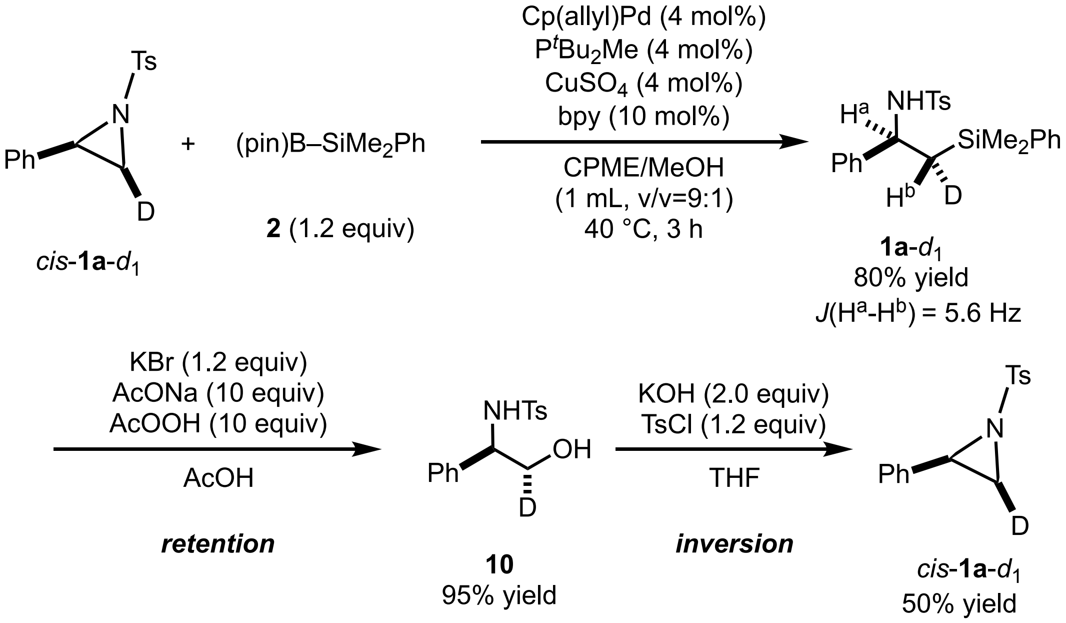

Procedure for the C(sp^3)–Si coupling of cis-1a-d_1. The coupling was conducted according to the typical procedure *(A typical procedure for the C3-selective ring-opening C(sp^3)–Si cross-coupling).*

***N*-(2-(dimethyl(phenyl)silyl)-1-phenylethyl-2-deuterium)-4-methylbenzenesulfonamide (1a-*d_1*)**

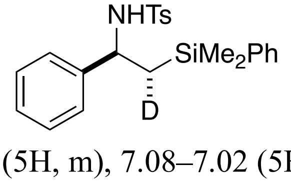

Purified by silica gel column chromatography (SiO_2 silica, *n*-hexane/EtOAc, 10:0 to 8:2). 80% yield; colorless solid; R_f 0.38 (*n*-hexane/EtOAc = 8:2); mp 115.1 °C; ^{1}H NMR (400 MHz, $CDCl_3$): δ 7.45 (2H, d, J = 8.4 Hz), 7.36–7.32 (5H, m), 7.08–7.02 (5H, m), 6.92 (2H, d, J = 8.4 Hz), 4.59 (1H, d, J = 6.4 Hz), 4.36–4.33 (1H, m), 2.34 (3H, s), 1.43 (1H, d, J = 5.2 Hz), 0.028 (3H, s), 0.005 (3H, s); ^{13}C NMR (100 MHz, $CDCl_3$): δ 142.5, 141.8, 137.8, 137.4, 133.4, 129.0, 128.9, 128.1, 127.6, 127.2, 126.9 , 126.4, 56.0, 25.7 (t, $J_{C\text{-}D}$ = 18.1 Hz), 21.3, –2.6, –3.4; IR (ATR, cm^{-1}): 3228, 3066, 2947, 2881, 1600, 1494, 1440, 1323, 1149, 1112,

1085, 1029, 916, 885, 812, 736; MS (EI$^+$) *m/z* (relative intensity, %): 409 ([M–H]$^+$, 2), 395 ([M–Me]$^+$, 33), 290 ([M–120]$^+$, 100), 228 (49), 135 ([SiMe$_2$Ph]$^+$, 82); HRMS (DART$^-$): calcd for $C_{23}H_{25}NO_2SSiD$ ([M–H]$^+$), 409.1528, found 409.1542.

Procedure for the Fleming oxidation of 1a-d$_1$. To a 200 mL flask, was added **1a**-*d*$_1$ (100 mg, 0.24 mmol), AcOH (2.5 mL), and the resulting solution was cooled with ice bath to 0 °C. To the solution, KBr (33.3 mg, 0.28 mmol, 1.2 equiv) and AcONa (19.7 mg, 0.24 mmol, 1 equiv) were added, and the resulting solution was stirred for 5 min. To the flask, AcOOH (10 % in AcOH, 1 mL) was added, and the resulting mixture was stirred at 0 °C for 1 h. After the ice bath was removed, AcONa (177 mg, 2.2 mmol, 9 equiv) and AcOOH (10% in AcOH, 10 mL) were added to the solution, and the resulting mixture was further stirred at room temperature for 12 h. $Na_2S_2O_3$ (3 g) and Et_2O (20 mL) were added to the mixture, and the resulting mixture was stirred for 1 h. The mixture was filtrated through the Celite pad, and the filtrate was washed with $NaHCO_3$ aq. and brine, and organic layer was extracted with Et_2O, which then was dried under reduced pressure to give amino alcohol **10**. The next reaction was conducted without further purification.

***N*-(2-(dimethyl(phenyl)silyl)-1-(*p*-tolyl)ethyl)-4-methylbenzenesulfonamide (10)**

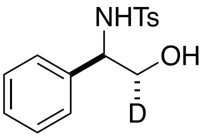

Extracted from Et_2O. 95% yield; colorless liquid; R_f 0.05 (*n*-hexane/EtOAc = 8:2); ^{1}H NMR (400 MHz, $CDCl_3$): δ 7.61 (2H, d, J = 8.0 Hz), 7.21–7.08 (7H, m), 5.44 (1H, d, J = 6.8 Hz), 4.41–4.38 (1H, m), 3.72 (1H, d, J = 5.6 Hz), 2.37 (3H, s), 2.10 (1H, br); ^{13}C NMR (100 MHz, $CDCl_3$): δ 143.2, 137.4, 136.9, 129.3, 128.4, 127.7, 127.0, 126.8, 65.7 (t, $J_{C–D}$ = 21.4), 59.5, 21.4; MS (EI$^+$) *m/z* (relative intensity, %): 206 (100), 155 ([Ts]$^+$, 53), 91 ([tolyl]$^+$, 72); HRMS (DART$^-$): calcd for $C_{15}H_{15}NO_3SD$ ([M–H]$^+$), 291.091, found 291.090.

Procedure for the intramolecular cyclization of 10. To a 50 mL flask, were added amino alcohol **10** (12.0 mg, 0.04 mmol), KOH (4.5 mg, 0.08 mmol, 2.0 equiv), and THF (6 mL), and the resulting mixture was stirred at room temperature for 10 min. To the mixture, TsCl (9.4 mg, 48 μmol, 1.2 equiv) was added, and the resulting mixture was stirred at 60 °C n an aluminum heating block for 12 h. The reaction mixture was washed with brine, and organic layer was extracted with Et_2O, which then was dried under reduced pressure. The residue was purified by flash chromatography on silica gel to give *cis*-**1a**-*d*$_1$.

A typical procedure for the C2-selective ring-opening C(sp^3)–Si cross-coupling. To a 3 mL vial with a magnetic stir (10 mm) bar, were added aziridine **1** (0.50 mmol) and 1,10'-phenanthroline (9.0 mg, 50 μmol, 10 mol%). And the vial was transferred into a glove box. Inside the glove box, to the vial, were added CuF_2 (1.9 mg, 20 μmol, 4 mol%), SIPr–Pd–PPh_3 (7.5 mg, 10 μmol, 2 mol%), CPME (2.25 mL), (pin)B–$SiMe_2$Ph **2** (157.2 mg, 0.60 mmol, 1.2 equiv), and MeOH (250 μL). The vial was capped with a hole cap and a Teflon®/rubber septum and removed from the glove box. The resulting mixture was stirred at 50 °C on an aluminum heating block for 3 h. The reaction mixture was filtrated through the Celite pad (2.0 cm height), and the residue was washed with CH_2Cl_2 (10 mL × 3). The filtrate was dried by evaporating the solvents under vacuum. Yields of products were estimated based on ^{1}H NMR integration

of the crude product using 1,1,2,2-tetrachloroethane as an internal standard. The coupled product were isolated by flash chromatography on silica gel.

(*S*)-*N*-(2-(Dimethyl(phenyl)silyl)-2-phenylethyl)-4-methylbenzenesulfonamide ((*S*)-8a)

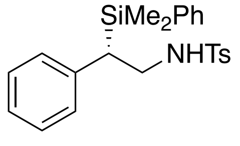

Purified by silica gel column chromatography (SiO_2 silica, *n*-hexane/EtOAc, 10:0 to 8:2). 89% yield; 99%ee (HPLC); colorless solid; R_f 0.31 (*n*-hexane/EtOAc = 8:2); mp 100.8 °C; ^{1}H NMR (400 MHz, $CDCl_3$): δ 7.55 (2H, d, *J* = 8.0 Hz), 7.38–7.11 (10H, m), 6.67 (2H, d, *J* = 8.0 Hz), 4.01 (1H, dd, *J* = 8.0, 2.4 Hz), 3.40 (1H, ddd, *J* = 12.8, 8.0, 4.4 Hz), 3.25 (1H, ddd, *J* = 12.8, 12.8, 2.4 Hz), 2.45 (3H, s), 2.32 (1H, dd, *J* = 12.8, 4.4 Hz), 0.18 (3H, s), 0.14 (3H, s); ^{13}C NMR (100 MHz, $CDCl_3$): δ 143.2, 138.5, 136.6, 135.6, 133.8, 129.5, 129.4, 128.6, 127.79, 127.73, 1127.0, 125.7, 43.3, 36.9, 21.5, –4.1, –5.4; ^{29}Si NMR (79 MHz, $CDCl_3$): δ –2.86; IR (ATR, cm^{-1}): 3271, 3022, 2954, 1598, 1489, 1323, 1253, 1112, 1055, 835, 813, 771, 721; MS (EI^+) *m/z* (relative intensity, %): 409 ($[M]^+$, 1), 394 ($[M–Me]^+$, 2), 290 ($[M–119]^+$, 32), 135 ($[SiMe_2Ph]^+$, 73), 104 (100); HRMS ($DART^-$): calcd for $C_{23}H_{27}NO_2SSi$ ($[M]^+$), 409.1532, found 409.1524; HPLC (Chiralcel OJ; 1.0 mL/min; *i*-PrOH/*n*-hexane 10:90; λ = 254 nm): t_S = 10.2 min (retention times of racemate: t_S = 10.2 min, t_R = 14.6 min); $[\alpha]_D^{20}$= +1.3 (*c* 1.0, $CHCl_3$). The absolute stereochemistry of the product was determined by the X-ray crystallography of its single crystal grown from *n*-hexane by slow solvent evaporation technique. For the detailed crystallographic data, see the “Single Crystal X-ray Crystallographic Data” section and S-8a.cif file.

***N*-(2-(Dimethyl(phenyl)silyl)-2-(*p*-tolyl)ethyl)-4-methylbenzenesulfonamide (8b)**

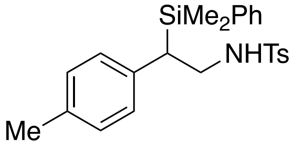

Purified by silica gel column chromatography (SiO_2 silica, *n*-hexane/EtOAc, 10:0 to 8:2). 78% yield; colorless solid; R_f 0.33 (*n*-hexane/EtOAc = 8:2); mp 128.5 °C; ^{1}H NMR (400 MHz, $CDCl_3$): δ 7.53 (2H, d, *J* = 8.4 Hz), 7.36–7.28 (5H, m), 7.22 (2H, d, *J* = 8.4 Hz), 6.94 (2H, d, *J* = 7.6 Hz), 6.57 (2H, d, *J* = 7.6 Hz), 4.27 (1H, dd, *J* = 8.8, 2.4 Hz), 3.36 (1H, ddd, *J* = 12.8, 8.8, 4.4 Hz), 3.25 (1H, ddd, *J* = 12.8, 12.8, 2.4 Hz), 2.42 (3H, s), 2.27 (1H, dd, *J* = 12.8, 4.4 Hz), 2.26 (3H, s), 0.16 (3H, s), 0.12 (3H, s); ^{13}C NMR (100 MHz, $CDCl_3$): δ 143.1, 136.5, 135.8, 135.1, 135.0, 133.8, 129.4, 129.3, 129.2, 127.6, 127.5, 126.9, 43.3, 36.2, 21.4, 20.8, –4.1, –5.5; ^{29}Si NMR (79 MHz, $CDCl_3$): δ –2.72; IR (ATR, cm^{-1}): 3250, 2962, 2860, 1512, 1479, 1427, 1315, 1303, 1247, 1163, 1112, 1055, 914, 839, 808, 740, 704; MS (EI^+) *m/z* (relative intensity, %): 423 ($[M]^+$, 1), 408 ($[M–Me]^+$, 1), 306 (40), 135 ($[SiMe_2Ph]^+$, 42), 118 (100); HRMS ($DART^-$): calcd for $C_{23}H_{27}NO_2SSi$ ($[M]^+$), 423.1688, found 423.1695.

(*S*)-*N*-(2-(Dimethyl(phenyl)silyl)-2-(4-fluorophenyl)ethyl)-4-methylbenzenesulfonamide (8c)

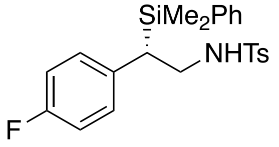

Purified by silica gel column chromatography (SiO_2 silica, *n*-hexane/EtOAc, 10:0 to 8:2). 70% yield; 99%ee (HPLC); colorless solid; R_f 0.30 (*n*-hexane/EtOAc = 8:2); mp 119.3 °C; ^{1}H NMR (400 MHz, $CDCl_3$): δ 7.55 (2H, d, *J* = 8.0 Hz), 7.40–7.24 (7H, m), 6.83 (2H, m), 6.61 (2H, m), 4.16 (1H, dd, *J* = 8.0, 3.2 Hz), 3.40 (1H, ddd, *J* = 12.8, 8.0, 4.4 Hz), 3.20 (1H, ddd, *J* = 12.8, 12.8, 3.2 Hz), 2.44 (3H, s), 2.32 (1H, dd, *J* =

12.8, 4.4 Hz), 0.18 (3H, s), 0.15 (3H, s); ^{13}C NMR (100 MHz, $CDCl_3$): δ 160.9 (d, $J_{C–F}$ = 242.9 Hz), 143.3, 136.5, 135.4, 134.28, 133.8, 129.5, 128.9 (d, $J_{C–F}$ = 8.2 Hz), 127.8, 127.0, 115.3 (d, $J_{C–F}$ = 20.6 Hz), 43.5, 36.3, 21.5, –4.2, –5.3; ^{29}Si NMR (79 MHz, $CDCl_3$): δ –2.75; IR (ATR, cm^{-1}): 3282, 3070, 2958, 1597, 1504, 1454, 1427, 1333, 1317, 1251, 1222, 1151, 1093, 1058, 997, 848, 810, 759, 734; MS (EI^+) *m/z* (relative intensity, %): 427 ($[M]^+$, 1), 412 ($[M–Me]^+$, 1), 290 ($[M–137]^+$, 49), 228 (72), 135 ($[SiMe_2Ph]^+$, 79), 122 (100); HRMS ($DART^–$): calcd for $C_{23}H_{26}FNO_2SSi$ ($[M]^+$), 427.1434, found 427.1438; HPLC (Chiralcel OJ; 1.0 mL/min; *i*-PrOH/*n*-hexane 10:90; λ = 254 nm): t_S = 32.3 min (retention times of racemate: t_R = 12.5 min, t_S = 32.3 min); $[\alpha]_D^{20}$= +1.0 (*c* 1.0, $CHCl_3$). The absolute configuration was tentatively conjectured by the sign of specific optical rotation.

(*S*)-*N*-(2-(4-Chlorophenyl)-2-(dimethyl(phenyl)silyl)ethyl)-4-methylbenzenesulfonamide (8d)

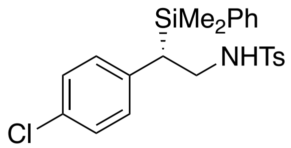

Purified by silica gel column chromatography (SiO_2 silica, *n*-hexane/EtOAc, 10:0 to 8:2). 86% yield; 97%ee (HPLC); colorless solid; R_f 0.30 (*n*-hexane/EtOAc = 8:2); mp 142.9 °C; ^{1}H NMR (400 MHz, $CDCl_3$): δ 7.53 (2H, d, *J* = 8.4 Hz), 7.40–7.23 (7H, m), 7.10 (2H, d, *J* = 8.8 Hz), 6.59 (2H, d, *J* = 8.8 Hz), 4.20 (1H, dd, *J* = 8.0, 3.6 Hz), 3.39 (1H, ddd, *J* = 12.8, 8.0, 3.6 Hz), 3.25 (1H, ddd, *J* = 12.8, 12.8, 3.6 Hz), 2.44 (3H, s), 2.33 (1H, dd, *J* = 12.8, 3.6 Hz), 0.18 (3H, s), 0.16 (3H, s); ^{13}C NMR (100 MHz, $CDCl_3$): δ 143.3, 137.3, 136.5, 135.2, 133.8, 131.2, 129.6, 129.5, 128.9, 128.6, 127.8, 126.9, 43.3, 36.7, 21.5, –4.2, –5.4; ^{29}Si NMR (79 MHz, $CDCl_3$): δ –2.75; IR (ATR, cm^{-1}): 3248, 3022, 2954, 2864, 1598, 1490, 1479, 1427, 1315, 1249, 1163, 1093, 1053, 1012, 914, 839, 808, 740, 727, 704; MS (EI^+) *m/z* (relative intensity, %): 443 ($[M]^+$, 1), 428 ($[M–Me]^+$, 1), 290 ($[M–153]^+$, 54), 228 (76), 138 (100), 135 ($[SiMe_2Ph]^+$, 93); HRMS ($DART^–$): calcd for $C_{23}H_{26}ClNO_2SSi$ ($[M]^+$), 443.1142, found 443.1145; HPLC (Chiralcel OJ; 1.0 mL/min; *i*-PrOH/*n*-hexane 10:90; λ = 254 nm): t_S = 31.0 min (retention times of racemate: t_R = 13.1 min, t_S = 31.0 min); $[\alpha]_D^{20}$= +0.2 (*c* 1.0, $CHCl_3$). The absolute configuration was tentatively conjectured by the sign of specific optical rotation.

***N*-(2-(Dimethyl(phenyl)silyl)-2-(4-(trifluoromethyl)phenyl)ethyl)-4-methylbenzenesulfonamide (8e)**

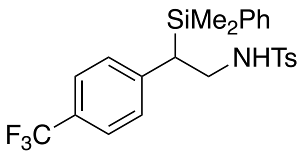

Purified by silica gel column chromatography (SiO_2 silica, *n*-hexane/EtOAc, 10:0 to 8:2). 84% yield; colorless solid; R_f 0.30 (*n*-hexane/EtOAc = 8:2); mp 102.9 °C; ^{1}H NMR (400 MHz, $CDCl_3$): δ 7.50 (2H, d, *J* = 8.4 Hz), 7.39–7.20 (9H, m), 6.77 (2H, d, *J* = 8.0 Hz), 4.35 (1H, dd, *J* = 6.8, 4.4 Hz), 3.39 (1H, ddd, *J* = 13.2, 6.8, 4.4 Hz), 3.29 (1H, ddd, *J* = 13.2, 13.2, 4.4 Hz), 2.46 (1H, dd, *J* = 13.2, 4.4 Hz), 2.43 (3H, s), 0.19 (3H, s), 0.17 (3H, s); ^{13}C NMR (100 MHz, $CDCl_3$): δ 143.5, 143.3, 136.4, 134.9, 133.8, 129.7, 129.5, 127.9, 127.8, 127.6 (q, $J_{C–F}$ = 32.1 Hz), 126.9, 125.2 (q, $J_{C–F}$ = 4.1 Hz), 124.1 (q, $J_{C–F}$ = 270.9 Hz), 43.1, 37.6, 21.4, –4.3, –5.4; ^{29}Si NMR (79 MHz, $CDCl_3$): δ –2.75; IR (ATR, cm^{-1}): 3280, 2956, 1614, 1427, 1321, 1249, 1184, 1149, 1122, 1060, 1014, 999, 852, 817, 798, 783, 761, 738, 702; MS (EI^+) *m/z* (relative intensity, %): 477 ($[M]^+$, 1), 462 ($[M–Me]^+$, 2), 290 ($[M–187]^+$, 86), 228 (100), 135 ($[SiMe_2Ph]^+$, 95); HRMS ($DART^–$): calcd for $C_{23}H_{26}ClNO_2SSi$ ($[M]^+$), 477.1406, found 477.1404.

N-(2-(Dimethyl(phenyl)silyl)-2-(p-tolyl)ethyl)-4-methylbenzenesulfonamide (8f)

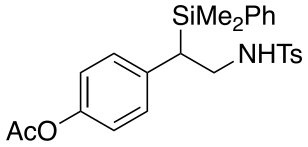

Purified by silica gel column chromatography (SiO_2 silica, *n*-hexane/EtOAc, 10:0 to 8:2). 80% yield; colorless solid; R_f 0.27 (*n*-hexane/EtOAc = 8:2); mp 109.2 °C; ^{1}H NMR (400 MHz, $CDCl_3$): δ 7.53 (2H, d, J = 8.4 Hz), 7.35–7.25 (5H, m), 7.22 (2H, d, J = 8.4 Hz), 6.86 (2H, d, J = 8.4 Hz), 6.66 (2H, d, J = 8.4 Hz), 4.47 (1H, dd, J = 8.0, 3.6 Hz), 3.37 (1H, ddd, J = 12.8, 8.0, 4.4 Hz), 3.21 (1H, ddd, J = 12.8, 12.8, 3.6 Hz), 2.41 (3H, s), 2.36 (1H, dd, J = 12.8, 4.4 Hz), 2.25 (3H, s), 0.17 (3H, s), 0.14 (3H, s); ^{13}C NMR (100 MHz, $CDCl_3$): δ 169.2, 148.3, 143.0, 136.4, 136.1, 135.4, 133.7, 129.4, 129.3, 128.3, 127.6, 126.8, 121.3, 43.3, 36.4, 21.3, 20.9, –4.3, –5.5; ^{29}Si NMR (79 MHz, $CDCl_3$): δ –2.68; IR (ATR, cm^{-1}): 3248, 2960, 1759, 1598, 1504, 1423, 1365, 1323, 1251, 1219, 1161, 1107, 1055, 1016, 912, 848, 810, 736, 702; MS (EI$^+$) *m/z* (relative intensity, %): 467 ([M]$^+$, 1), 452 ([M–Me]$^+$, 1), 290 ([M–177]$^+$, 10), 306 (29), 135 ([$SiMe_2Ph$]$^+$, 84), 120 (100); HRMS (DART$^-$): calcd for $C_{25}H_{29}NO_4SSi$ ([M]$^+$), 467.1587, found 467.1595.

Methyl 4-(1-(dimethyl(phenyl)silyl)-2-((4-methylphenyl)sulfonamido)ethyl)benzoate (8g)

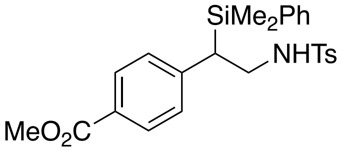

Purified by silica gel column chromatography (SiO_2 silica, *n*-hexane/EtOAc, 10:0 to 8:2). 54% yield; colorless solid; R_f 0.18 (*n*-hexane/EtOAc = 8:2); mp 141.1 °C; ^{1}H NMR (400 MHz, $CDCl_3$): δ 7.81 (2H, d, J = 8.4 Hz), 7.54 (2H, d, J = 8.0 Hz), 7.41–7.24 (7H, m), 6.72 (2H, d, J = 8.4 Hz), 4.10 (1H, dd, J = 8.0, 3.6 Hz), 3.91 (3H, s), 3.43 (1H, ddd, J = 13.2, 8.0, 4.4 Hz), 3.29 (1H, ddd, J = 13.2, 13.2, 3.2 Hz), 2.46 (1H, m), 2.45 (3H, s), 0.19 (3H, s), 0.17 (3H, s); ^{13}C NMR (100 MHz, $CDCl_3$): δ 166.8, 144.8, 143.2, 136.4, 135.0, 133.7, 129.59, 129.55, 129.4, 127.7, 127.5, 127.2, 126.8, 51.8, 43.0, 37.7, 21.4, –4.3, –5.4; IR (ATR, cm^{-1}): 3325, 2958, 1705, 1429, 1325, 1288, 1157, 1141, 1112, 1093, 1058, 1016, 906, 858, 812, 773, 721, 700; MS (EI$^+$) *m/z* (relative intensity, %): 467 ([M]$^+$, 1), 452 ([M–Me]$^+$, 1), 290 ([M–177]$^+$, 99), 228 (90), 135 ([$SiMe_2Ph$]$^+$, 15); HRMS (DART$^+$): calcd for $C_{25}H_{30}NO_4SSi$ ([M]$^+$), 468.1659, found 467.1660.

A typical procedure for the C2-selective ring-opening tandem reaction. To a 3 mL vial with a magnetic stir (10 mm) bar, were added aziridine **1** (0.20 mmol), 2,2'-bipyridine (3.12 mg, 20 μmol, 10 mol%), CuF_2 (0.81 mg, 8 μmol, 4 mol%), PPh_3 (2.1 mg, 8 μmol, 4 mol%). And the vial was transferred into a glove box. Inside the glove box, to the vial, were added SIPr–Pd–PPh_3 (3.04 mg, 4 μmol, 2 mol%), toluene (900 μL), and MeOH (100 μL), and (pin)B–$SiMe_2Ph$ **2** (62.9 mg, 0.24 mmol, 1.2 equiv). The vial was capped with a hole cap and a Teflon®/rubber septum and removed from the glove box. The resulting mixture was stirred at 50 °C on an aluminum heating block for 4 h. The reaction mixture was filtrated through the Celite pad (2.0 cm height), and the residue was washed with CH_2Cl_2 (10 mL × 3). The filtrate was dried by evaporating the solvents under vacuum. Yields of products were estimated based on ^{1}H NMR integration of the crude product using 1,1,2,2-tetrachloroethane as an internal standard. The products were isolated by flash chromatography on silica gel.

N-[1-(Dimethylphenylsilyl)-2-phenylethyl]-4-methylbenzenesulfonamide (9a)

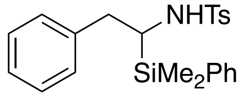

Purified by silica gel column chromatography (n-hexane/EtOAc, 10:0 to 8:2). 87% yield; colorless oil; R_f 0.58 (n-hexane/EtOAc = 8:2); ^{1}H NMR (400 MHz, $CDCl_3$): δ 7.55–7.53 (2H, m), 7.41–7.31 (5H, m), 7.16–7.12 (5H, m), 6.99–6.97 (2H, m), 4.21 (1H, d, J = 8.8 Hz), 3.32–3.26 (1H, m), 2.77 (1H, dd, J = 14.0, 7.6 Hz), 2.68 (1H, dd, J = 14.0, 6.4 Hz), 2.38 (3H, s), 0.14 (3H, s), 0.11 (3H, s); ^{13}C NMR (100 MHz, $CDCl_3$): δ 142.7, 138.6, 137.7, 135.3, 134.0, 129.4, 129.4, 129.2, 128.2, 127.9, 126.8, 126.2, 45.2, 38.0, 21.4, –4.6, –4.9; IR (ATR, cm^{-1}): 3285, 3048, 2922, 2253, 1709, 1597, 1514, 1427, 1321, 1250, 1155, 1113, 1094, 1040, 908, 833, 812, 779, 733, 703; MS (FAB$^-$) m/z (relative intensity, %): 408 ([M–H]$^+$, 79), 306 (20), 199 (18), 168 (28) 153 (100), 46 (21); HRMS (FAB$^-$): calcd for $C_{23}H_{26}NO_2SSi$ ([M–H]$^+$), 408.1460.

N-[1-(dimethylphenylsilyl)-2-(4-methylphenylethyl)]-4-methylbenzenesulfonamide (9b)

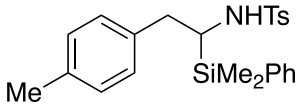

Purified by silica gel column chromatography (n-hexane/EtOAc, 10:0 to 8:2). 80% yield; colorless oil; R_f 0.28 (n-hexane/EtOAc = 8:2); ^{1}H NMR (400 MHz, $CDCl_3$) δ 7.53 (2H, d, J = 8.4 Hz), 7.40–7.29 (5H, m), 7.13 (2H, d, J = 8.0 Hz), 6.91 (2H, d, J = 8.0 Hz), 6.83 (2H, d, J = 8.4 Hz), 4.33 (1H, d, J = 8.8 Hz), 3.30–3.24 (1H, m), 2.72 (1H, dd, J = 14.4, 7.6 Hz), 2.60 (1H, dd, J = 14.0, 6.8 Hz), 2.38 (3H, s), 2.27 (3H, s), 0.17 (3H, s), 0.13 (3H, s); ^{13}C NMR (100 MHz, $CDCl_3$): δ 142.8, 137.8, 135.8, 135.4, 134.1, 129.5, 129.4, 129.1, 128.9, 128.0, 126.9, 45.5, 37.5, 21.5, 21.0, –4.4, –4.8, 1C is missing in the aromatic region, probably due to the overlap of signals in the aromatic region.; IR (ATR, cm^{-1}): 3265, 3024, 2955, 2922, 1599, 1454, 1427, 1321, 1250, 1153, 1113, 1094, 1042, 928, 907, 833, 812, 775, 734; MS (FAB$^-$) m/z (relative intensity, %): 422 ([M–H]$^+$, 84), 306 (30), 199 (26), 168 (30) 153 (100), 122 (12), 46 (21); HRMS (FAB$^-$): calcd for $C_{24}H_{28}NO_2SSi$ ([M–H]$^+$), 422.1616

N-[1-(dimethyl(phenyl)silyl)-2-((4-fluorophenyl)ethyl)]-4-methylbenzenesulfonamide (9c)

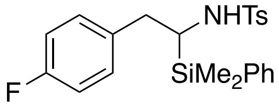

Purified by silica gel column chromatography (n-hexane/EtOAc, 10:0 to 8:2). 72% yield; colorless solid; R_f 0.48 (n-hexane/EtOAc = 8:2); mp 92.9 °C; ^{1}H NMR (400 MHz, $CDCl_3$): δ 7.49 (2H, d, J = 8.0 Hz), 7.40–7.32 (5H, m), 7.14 (2H, d, J = 8.0 Hz), 6.90 (2H, dd, $J_{H\text{–}H}$ = 8.8, $^4J_{H\text{–}F}$ = 5.6 Hz), 6.77 (2H, dd, $J_{H\text{–}H}$ = 8.8, $J_{H\text{–}F}$ = 8.8 Hz), 4.14 (1H, d, J = 8.8 Hz), 3.24 (1H, ddd, J = 8.8, 6.8, 6.8 Hz), 2.78 (1H, dd, J = 14.4, 6.8 Hz), 2.59 (1H, dd, J = 14.4, 6.8 Hz), 2.40 (3H, s), 0.20 (3H, s), 0.17 (3H, s); ^{13}C NMR (100 MHz, $CDCl_3$): δ 161.5 (d, $^1J_{C\text{–}F}$ = 242.9 Hz), 142.9, 137.7, 135.1, 134.0, 130.5 (d, $^3J_{C\text{–}F}$ = 8.2 Hz), 129.6, 129.4, 128.1, 126.8, 114.9 (d, $^2J_{C\text{–}F}$ = 20.6 Hz), 45.7, 37.1, 21.4, –4.5, –5.0, 1C is missing in the aromatic region, probably due to the overlap of signals in the aromatic region.; IR (ATR, cm^{-1}): 3289, 3040, 2918, 1599, 1504, 1427, 1321, 1263, 1219, 1148, 1115, 1090, 1051, 947, 926, 833, 800, 766; MS (EI$^+$) m/z (relative intensity, %): 412 ([M–CH_3]$^+$, 2), 318 ([M–C_6H_4F]$^+$, 64), 272 ([M–$SO_2C_6H_4CH_3$]$^+$, 76), 211 (38), 149 (28) 135 (100), 91 (20); HRMS (DART$^-$): calcd for $C_{23}H_{25}NO_2SSiF$ ([M–H]$^+$), 426.1364, found 426.1369.

***N*-[1-(dimethyl(phenyl)silyl)-2-((4-chlorophenyl)ethyl)]-4-methylbenzenesulfonamide (9d)**

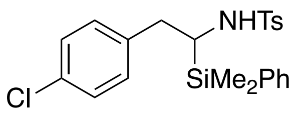

Purified by silica gel column chromatography (*n*-hexane/EtOAc, 10:0 to 8:2). 61% yield; colorless solid; R_f 0.48 (*n*-hexane/EtOAc = 8:2); mp 116.9 °C; ^{1}H NMR (400 MHz, $CDCl_3$) δ 7.45 (2H, d, *J* = 8.4 Hz), 7.43–7.32 (5H, m), 7.12 (2H, d, *J* = 8.0 Hz), 7.00 (2H, d, *J* = 8.4 Hz), 6.83 (2H, d, *J* = 8.4 Hz), 4.32 (1H, d, *J* = 8.8 Hz), 3.25 (1H, ddd, *J* = 8.8, 8.0, 6.4 Hz), 2.76 (1H, dd, *J* = 14.0, 6.4Hz), 2.53 (1H, dd, *J* = 14.0, 8.0 Hz), 2.41 (3H, s), 0.25 (3H, s), 0.22 (3H, s); ^{13}C NMR (100 MHz, $CDCl_3$): δ 142.9, 137.7, 137.3, 135.0, 134.0, 132.1, 130.4, 129.7, 129.4, 128.2, 128.1, 126.7, 45.7, 37.3, 21.5, –4.4, –5.1; IR (ATR, cm^{-1}): 3264, 3049, 2957, 1599, 1491, 1427, 1408, 1319, 1252, 1152, 1094, 1059, 1015, 953, 932, 801, 777, 731; MS (EI^+) *m/z* (relative intensity, %): 428 ($[M–CH_3]^+$, 2), 318 ($[M–C_6H_4Cl]^+$, 72), 288 ($[M–SO_2C_6H_4CH_3]^+$, 76), 211 (37),149 (27), 135 (100) 91 (24); HRMS ($DART^-$): calcd for $C_{23}H_{25}NO_2SSiCl$ ($[M–H]^+$), 442.1069, found 442.1071.

4-(2-(Dimethyl(phenyl)silyl)-2-((4-methylphenyl)sulfonamido)ethyl)phenyl acetate (9f)

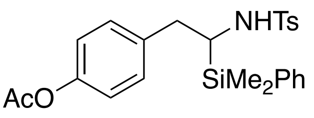

Purified by silica gel column chromatography (*n*-hexane/EtOAc, 10:0 to 8:2). 78% yield; colorless solid; R_f 0.25 (*n*-hexane/EtOAc = 8:2); mp 81.2 °C, ^{1}H NMR (400 MHz, $CDCl_3$) δ 7.52 (2H, d, *J* = 7.6 Hz), 7.42–7.30 (5H, m), 7.16 (2H, d, *J* = 8.4 Hz), 6.97 (2H, d, *J* = 8.4 Hz), 6.84 (2H, d, *J* = 8.4 Hz), 4.15 (1H, d, *J* = 8.8 Hz), 3.26 (1H, ddd, *J* = 8.8, 6.8, 6.8 Hz), 2.78 (1H, dd, *J* = 14.0, 7.2 Hz), 2.64 (1H, dd, *J* = 14.0, 6.8 Hz), 2.39 (3H, s), 2.29 (3H, s), 0.18 (3H, s), 0.14 (3H, s); ^{13}C NMR (100 MHz, $CDCl_3$): δ 169.4, 149.1, 142.9, 137.6, 136.2, 135.0, 134.0, 130.0, 129.6, 129.5, 128.0, 126.8, 121.2, 45.3, 37.3, 21.4, 21.1, –4.5, –4.9; IR (ATR, cm^{-1}): 3294, 3071, 2957, 1748, 1506, 1427, 1368, 1319, 1221, 1196, 1150, 1115, 1092, 1059, 1013, 951, 917, 812, 791, 734, 703; HRMS ($DART^+$): calcd for $C_{25}H_{30}NO_4SSi$ ($[M+H]^+$), 468.1659, found 468.1664.

Methyl 4-(2-(dimethyl(phenyl)silyl)-2-((4-methylphenyl)sulfonamido)ethyl)benzoate (9g)

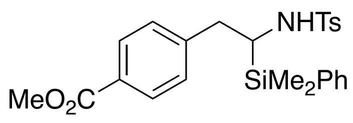

Purified by silica gel column chromatography (*n*-hexane/EtOAc, 10:0 to 8:2). 51% yield; colorless solid; R_f 0.28 (*n*-hexane/EtOAc = 8:2); mp 114.0 °C; ^{1}H NMR (400 MHz, $CDCl_3$) δ 7.74 (2H, d, *J* = 8.4 Hz), 7.45 (2H, d, *J* = 8.4 Hz), 7.43–7.32 (5H, m), 7.09 (2H, d, *J* = 7.6 Hz), 7.00 (2H, d, *J* = 8.4 Hz), 4.27 (1H, d, *J* = 8.8 Hz), 3.91 (3H, s), 3.34–3.28 (1H, m), 2.84 (1H, dd, *J* = 14.0, 6.8 Hz), 2.65 (1H, dd, *J* = 14.0, 7.6 Hz), 2.34 (3H, s), 0.22 (3H, s), 0.20 (3H, s); ^{13}C NMR (100 MHz, $CDCl_3$): δ 166.9, 144.5, 142.9, 137.7, 134.8, 134.0, 129.8, 129.5, 129.4, 129.2, 128.1, 126.8, 52.0, 45.5, 38.1, 21.4, –4.5, –5.0, 1C is missing in the aromatic region, probably due to the overlap of signals in the aromatic region.; IR (ATR, cm^{-1}): 3250, 2953, 1717, 1611, 1435, 1414, 1321, 1277, 1252, 1179, 1152, 1107, 1094, 1059, 1020, 962, 943, 845, 808, 773, 741; HRMS ($DART^+$): calcd for $C_{25}H_{30}NO_4SSi$ ($[M+H]^+$), 468.1659, found 468.1664.

Scheme S2. Control experiment: Cu-catalyzed silyl transfer from 2 to imine 11.

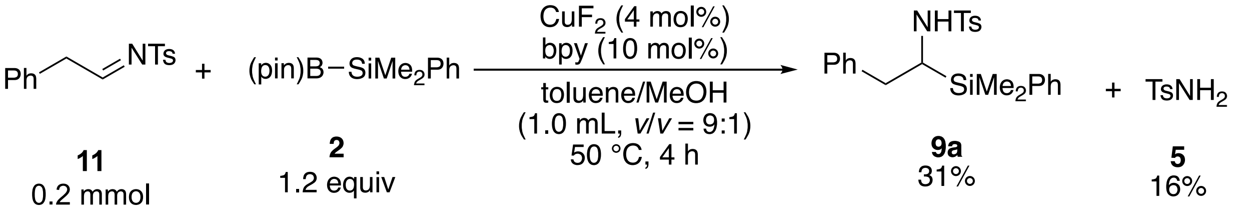

A procedure. To a 3 mL vial with a magnetic stir (10 mm) bar, were added imine **11** (52.0 mg, 0.20 mmol), 2,2'-bipyridine (3.12 mg, 20 μmol, 10 mol%), CuF_2 (0.81 mg, 8 μmol, 4 mol%). And the vial was transferred into a glove box. Inside the glove box, to the vial, were added toluene (900 μL) and MeOH (100 μL), and (pin)B–$SiMe_2Ph$ **2** (62.9 mg, 0.24 mmol, 1.2 equiv). The vial was capped with a hole cap and a Teflon®/rubber septum and removed from the glove box. The resulting mixture was stirred at 50 °C on an aluminum heating block for 1.5 h. The reaction mixture was filtrated through the Celite pad (2.0 cm height), and the residue was washed with CH_2Cl_2 (10 mL × 3). The filtrate was dried by evaporating the solvents under vacuum. Yields of products were estimated based on ^{1}H NMR integration of the crude product using 1,1,2,2-tetrachloroethane as an internal standard. The products were isolated by flash chromatography on silica gel.

Single Crystal X-ray Crystallographic Data of (*S*)-8a. The X-ray diffraction data of the single crystal of (*S*)-**8a** were collected on a two-dimensional X-ray detector (PILATUS 200K/R) equipped in Rigaku XtaLAB PRO diffractometer using thin multi-layer mirror monochromated Cu-Kα radiation (λ =1.54187 Å) to a $2\theta_{max}$ value of 148.8° at 93 K. The cell refinements were performed with a software CrysAlisPro 1.171.39.20a..[S15] The crystal structure was solved by direct methods (SHELXT Version 2014/5).[S16] All calculations were performed with the observed reflections [I > 2σ(I)] with the program CrystalStructure crystallographic software packages,[S17] except for refinement which was performed by SHELXL.[S18] The non-hydrogen atoms were refined anisotropically, and hydrogen atoms were refined using the riding model. The crystal data are summarized in Table S17. CCDC-1911669 contains the supplementary crystallographic data for (*S*)-**8a**, which are available free of charge from the Cambridge Crystallographic Data Center (CCDC) via www.ccdc.cam.ac.uk/data_request/cif

Table S17. Summary of the crystallographic data of (*S*)-**8a**.

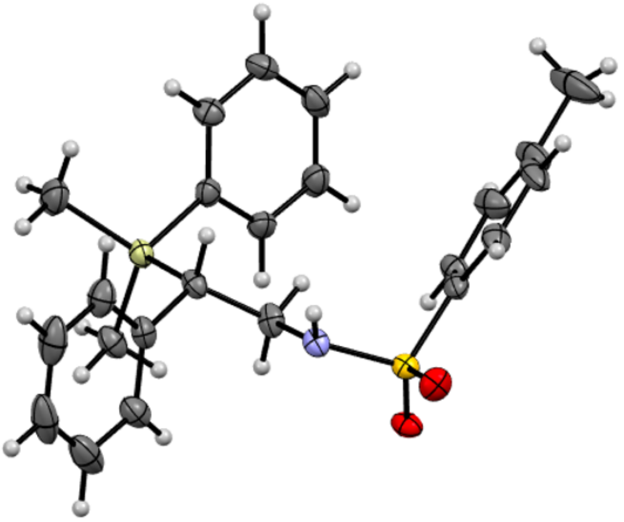

Empirical Formula	$C_{23}H_{27}NO_2SSi$	
Formula Weight	409.62	
Crystal System	monoclinic	
Space Group	$P2_1$ (#4)	
Unit cell dimensions	a = 9.63219(11) Å	α = 90°
	b = 9.47828(9) Å	β = 106.2700(12)°
	c = 12.72130(14) Å	γ = 90°
V	1114.90(2) Å^3	
Z	2	
Density (calculated)	1.220 g/cm^3	
Absorption coefficient	19.39 cm^{-1}	
R_1 [I>2σ(I)]	0.0352	
wR_2 (all data)	0.0950	
Crystal size	0.300 × 0.050 × 0.050 mm	
Goodness-of-fit on F^2	1.077	
Reflections collected/unique	12316/4387 [R(int) = 0.0352]	
Flack parameter	0.000(11)	

Copies of ^{1}H and ^{13}C NMR Charts

^{1}H NMR: (400 MHz, $CDCl_3$)

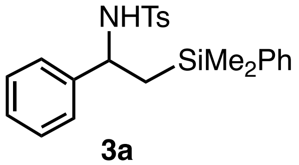

3a

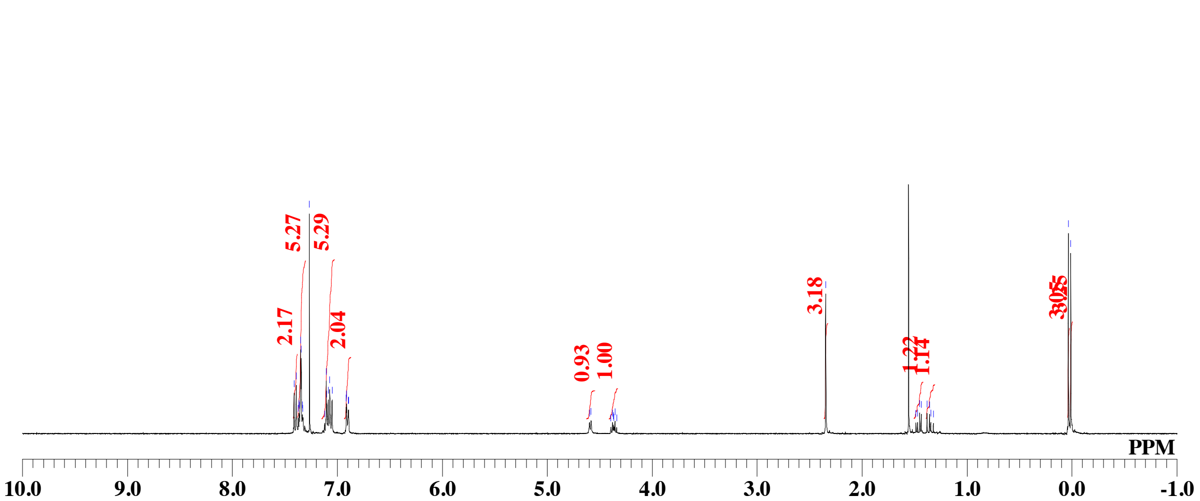

^{13}C NMR: (100 MHz, $CDCl_3$)

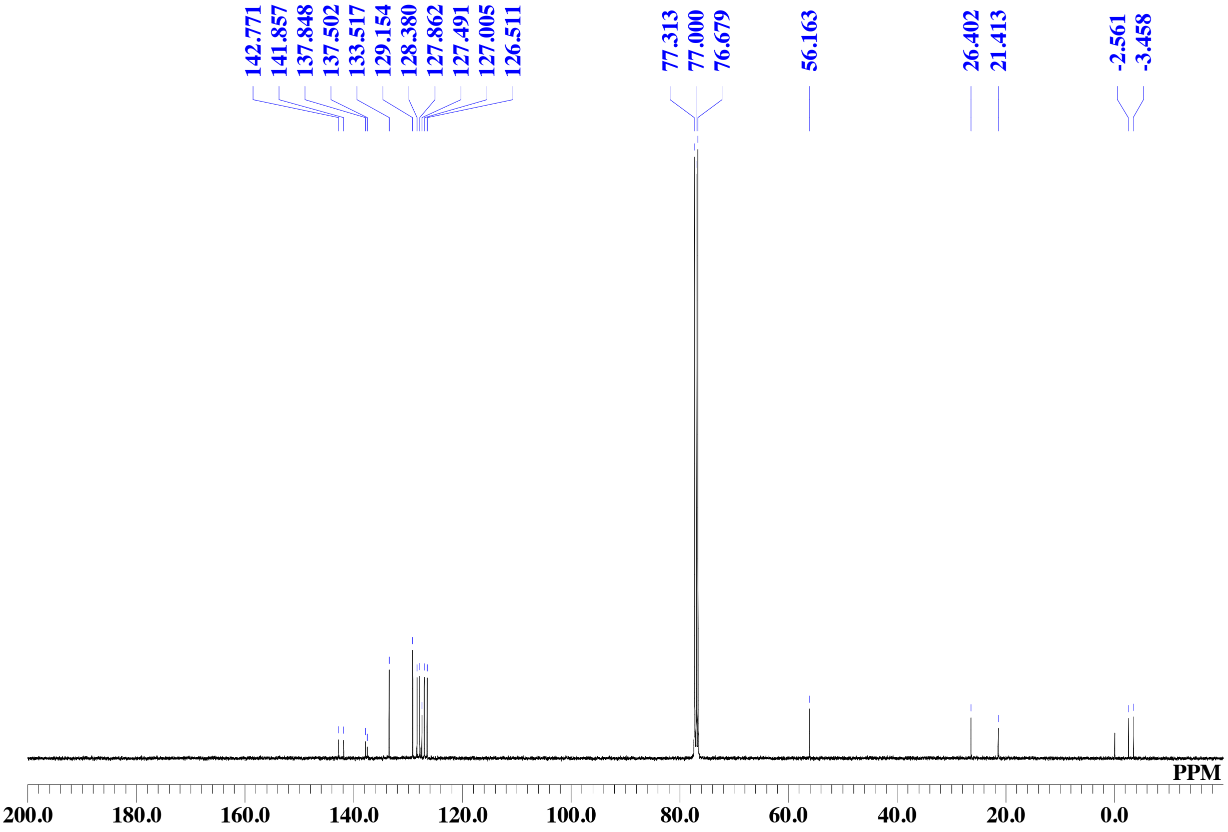

^{1}H NMR: (400 MHz, $CDCl_3$)

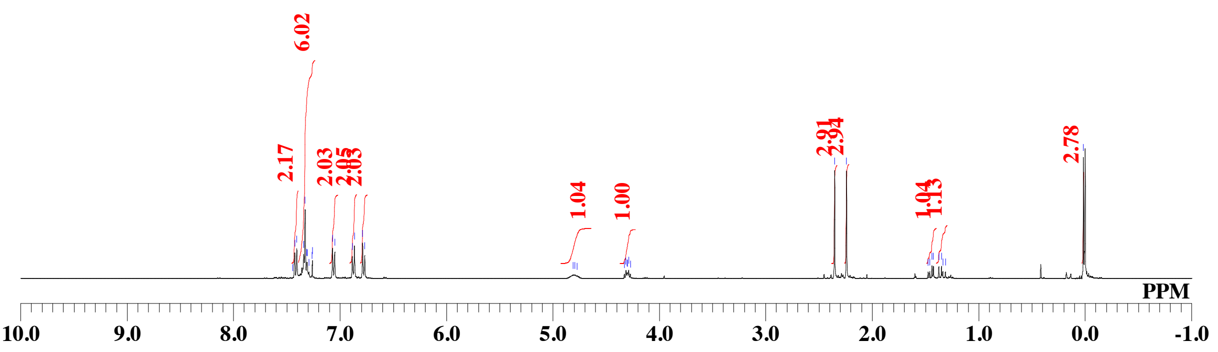

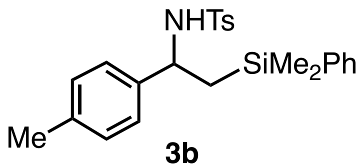

3b

^{13}C NMR: (100 MHz, $CDCl_3$)

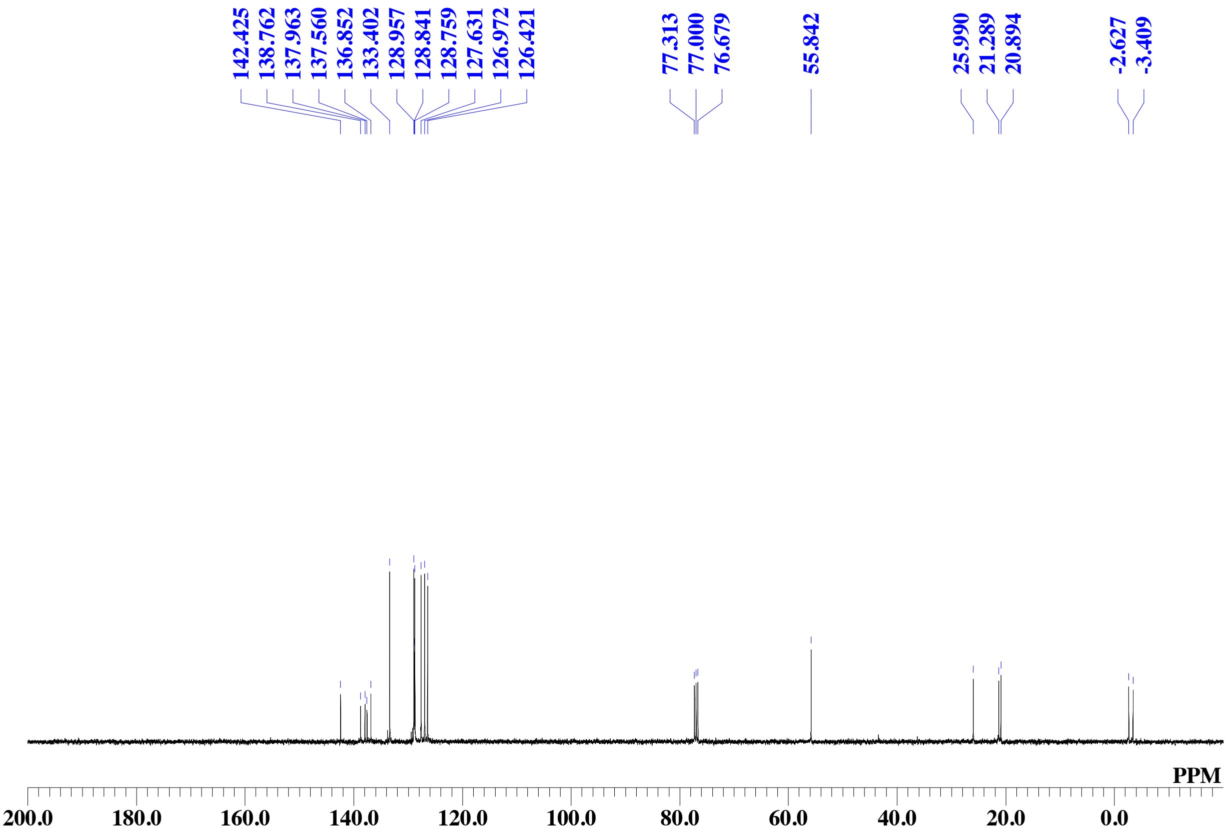

^{1}H NMR: (400 MHz, $CDCl_3$)

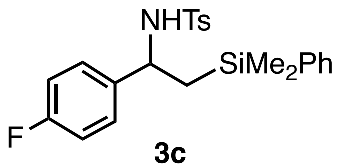

3c

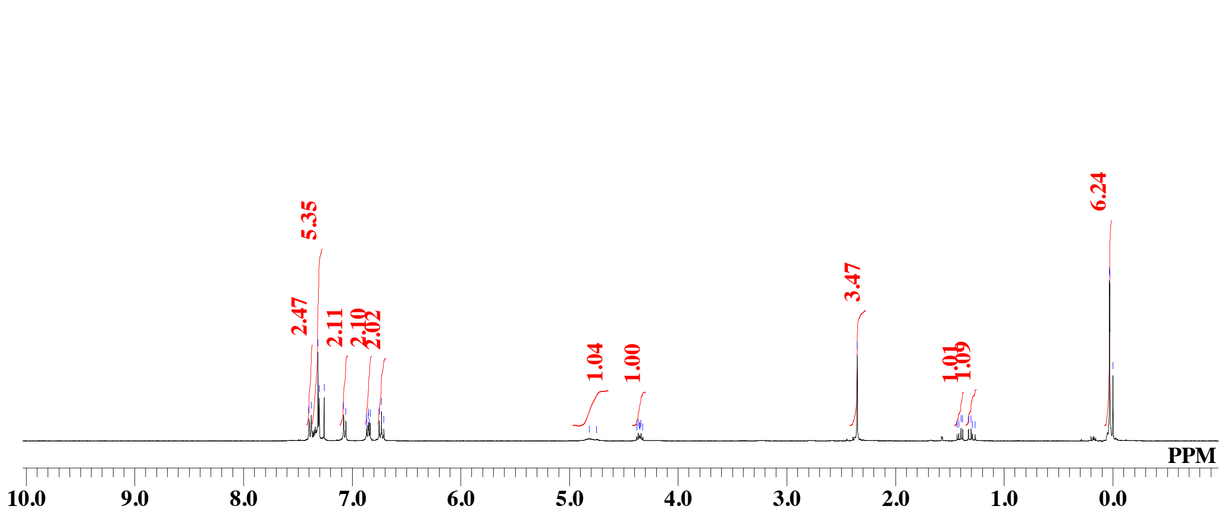

^{13}C NMR: (100 MHz, $CDCl_3$)

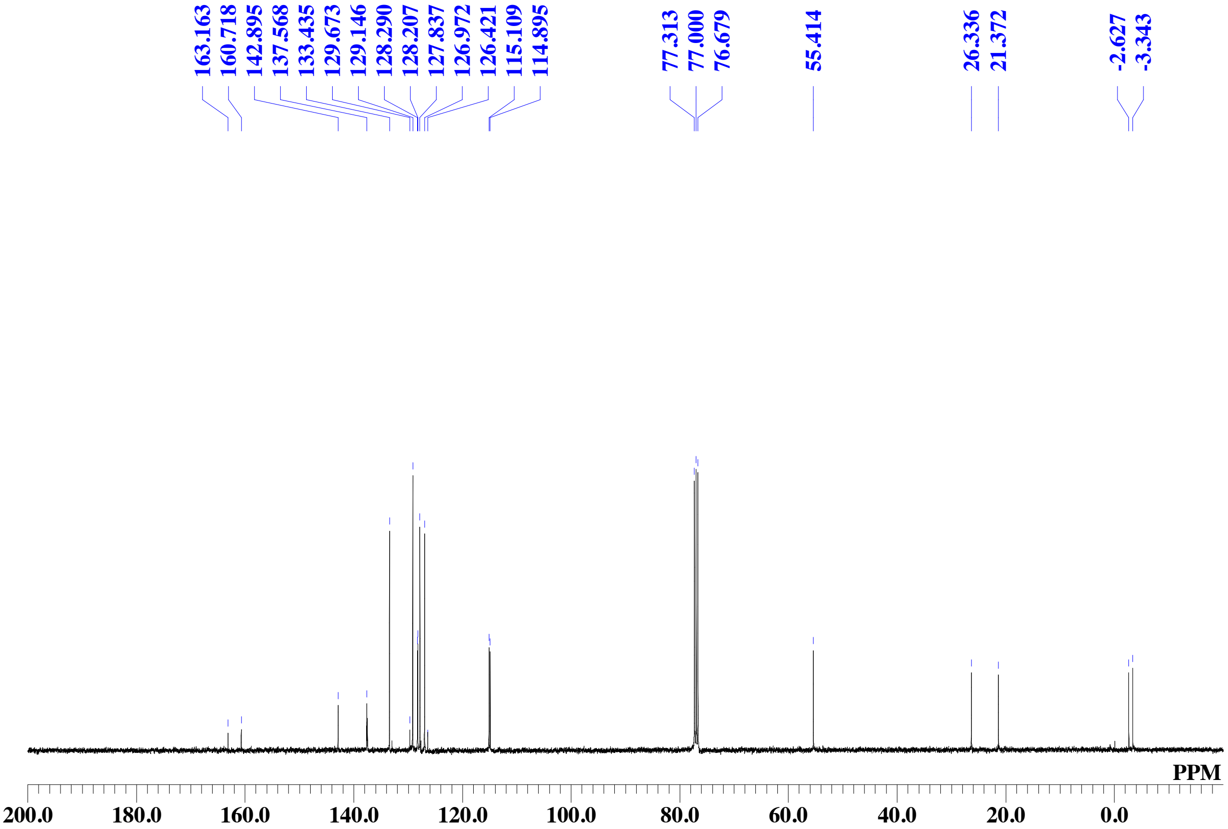

^{1}H NMR: (400 MHz, $CDCl_3$)

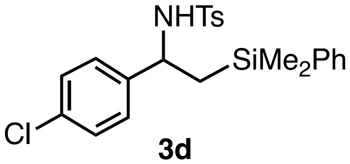

3d

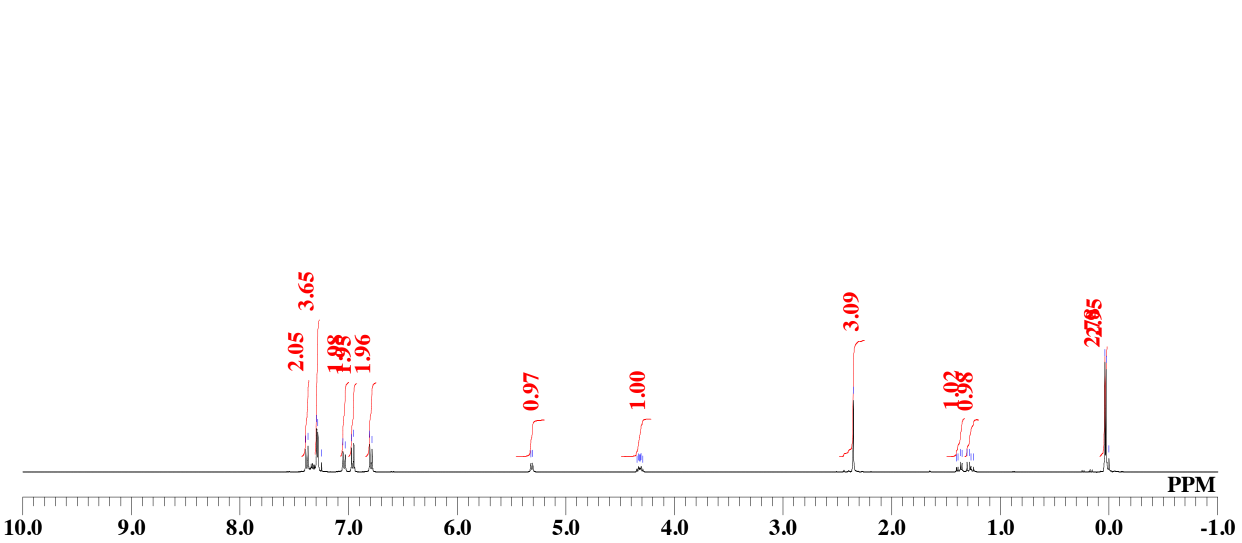

^{13}C NMR: (100 MHz, $CDCl_3$)

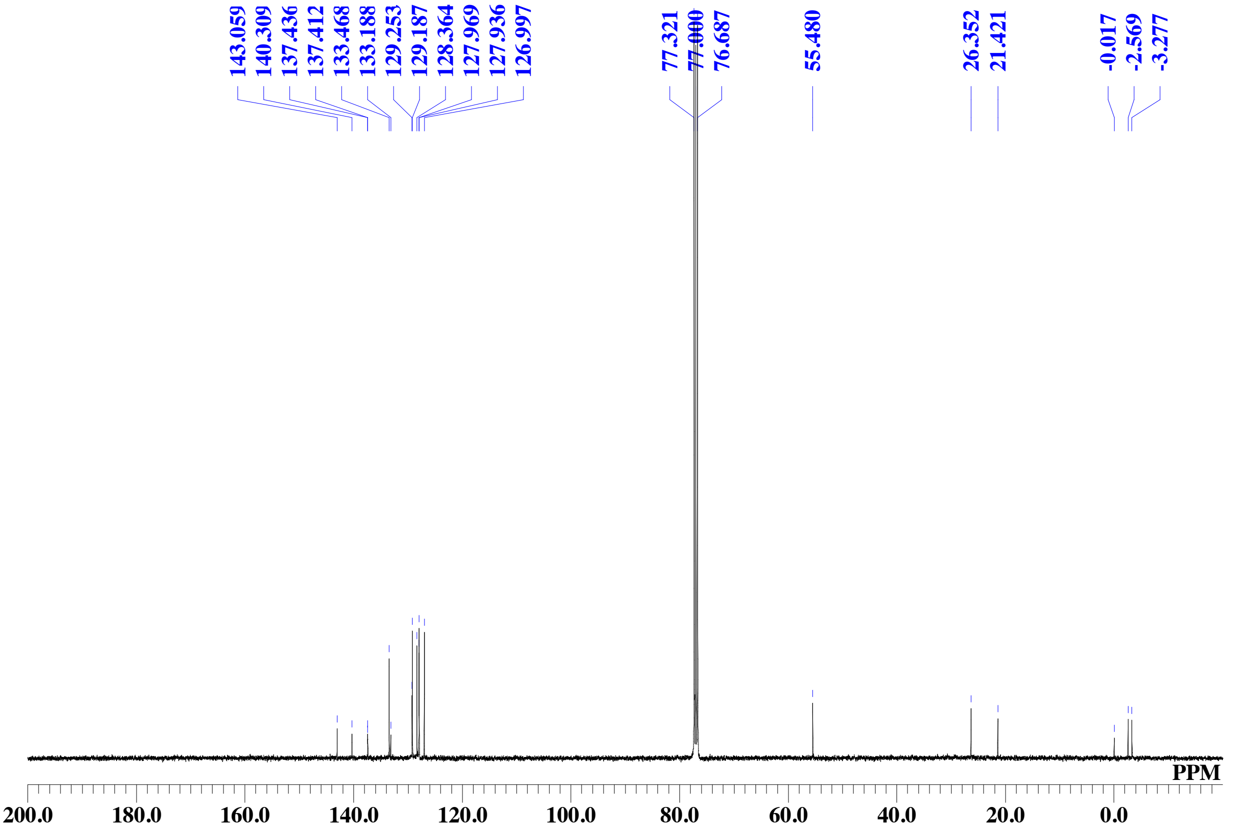

^{1}H NMR: (400 MHz, $CDCl_3$)

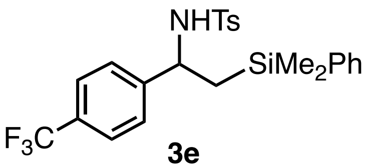

3e

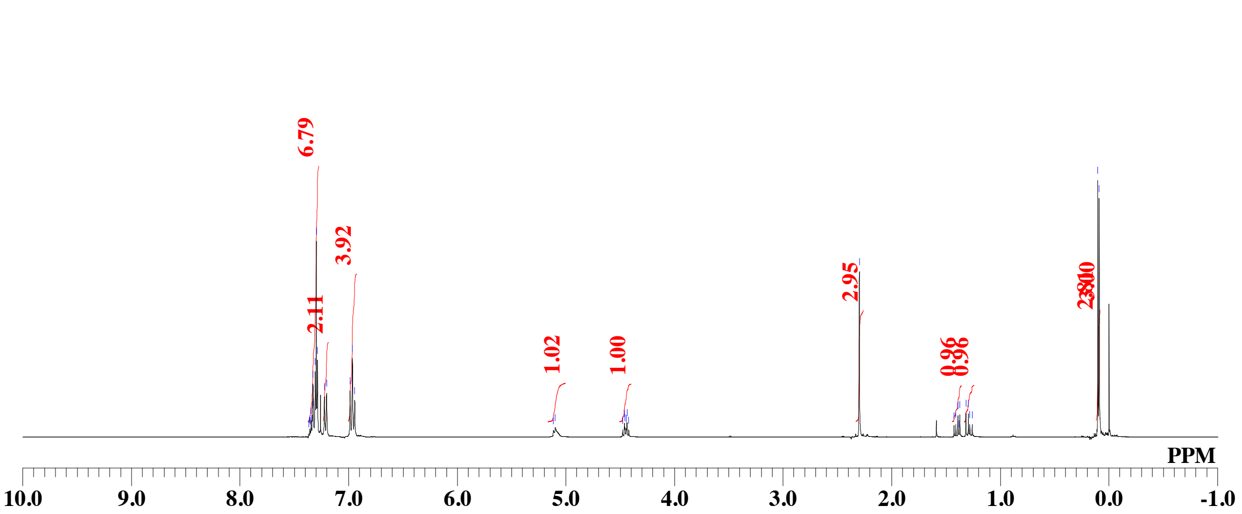

^{13}C NMR: (100 MHz, $CDCl_3$)

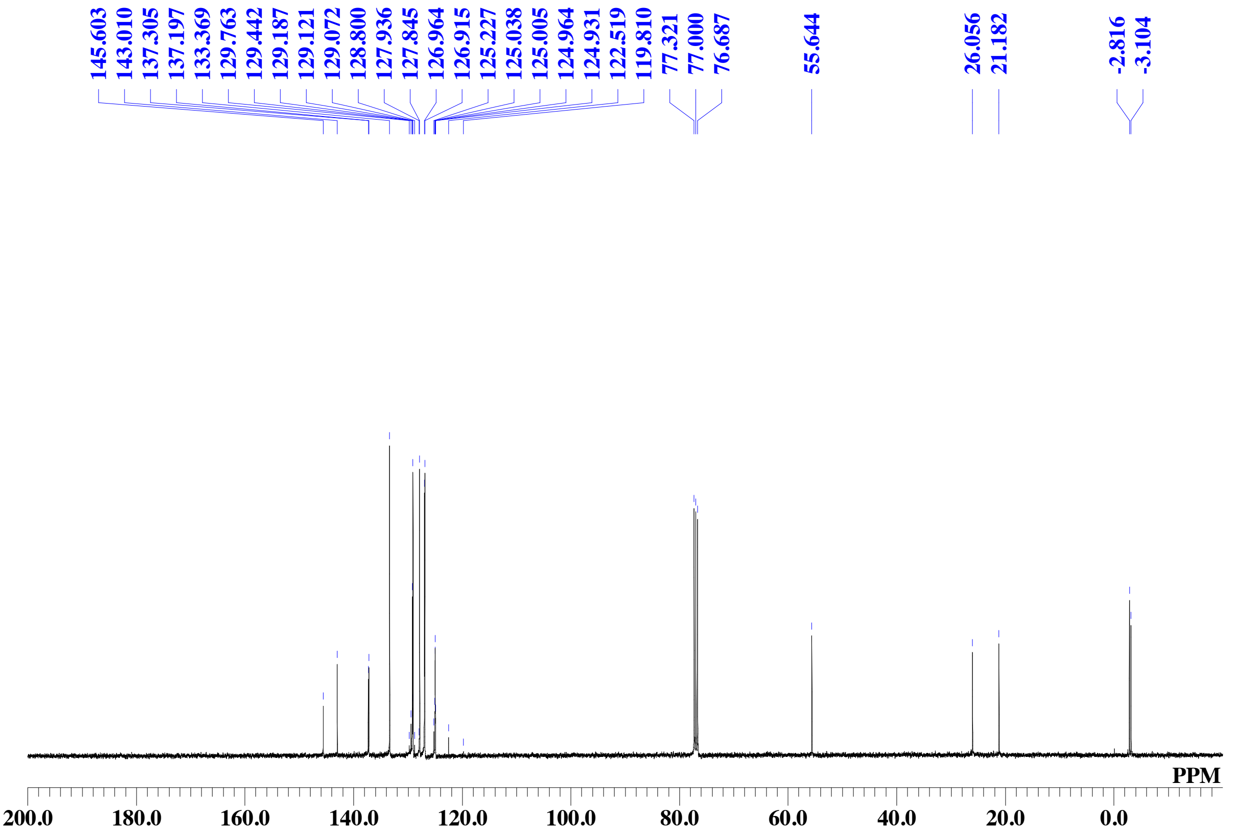

^{1}H NMR: (400 MHz, $CDCl_3$)

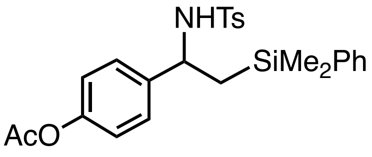

3f

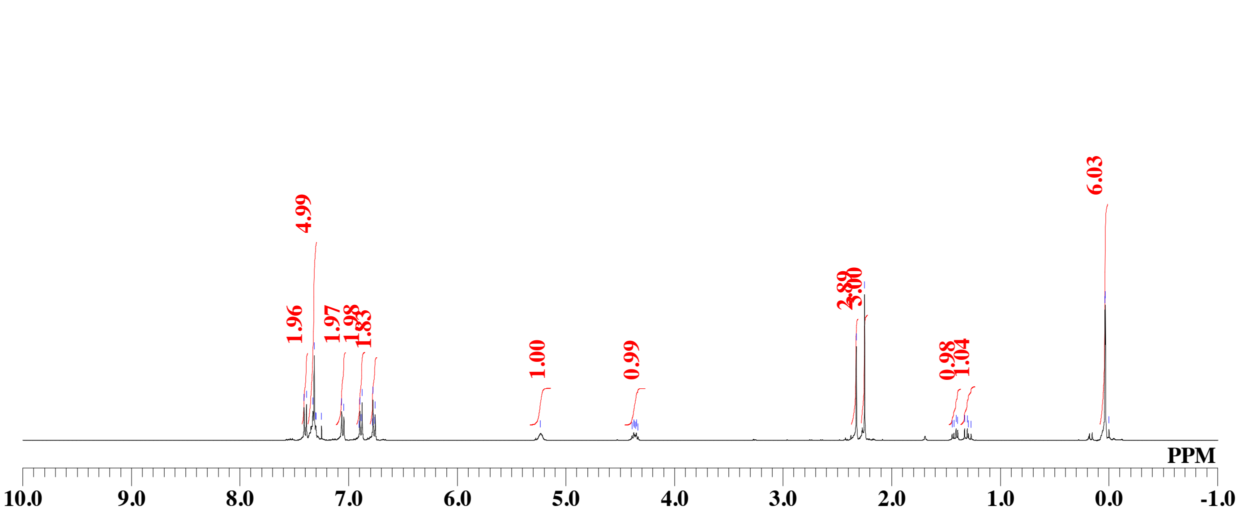

^{13}C NMR: (100 MHz, $CDCl_3$)

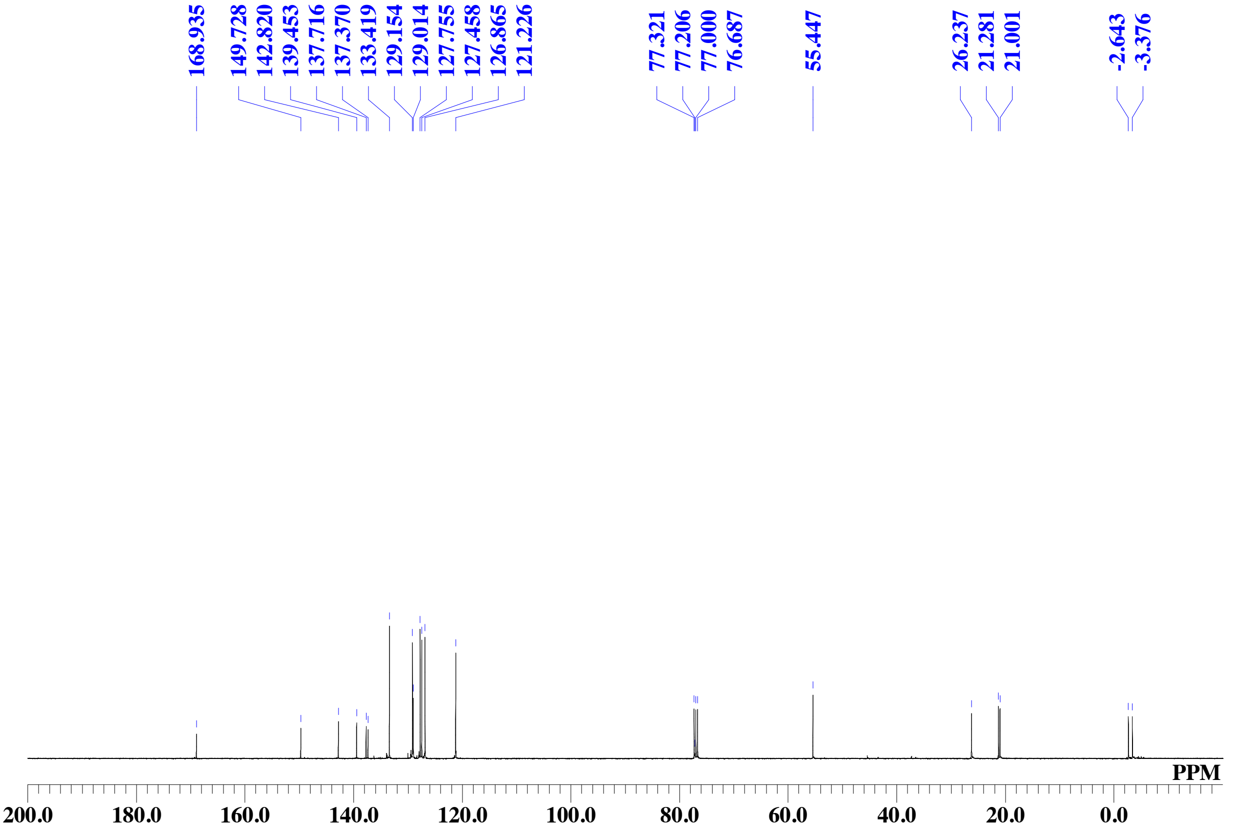

^{1}H NMR: (400 MHz, $CDCl_3$)

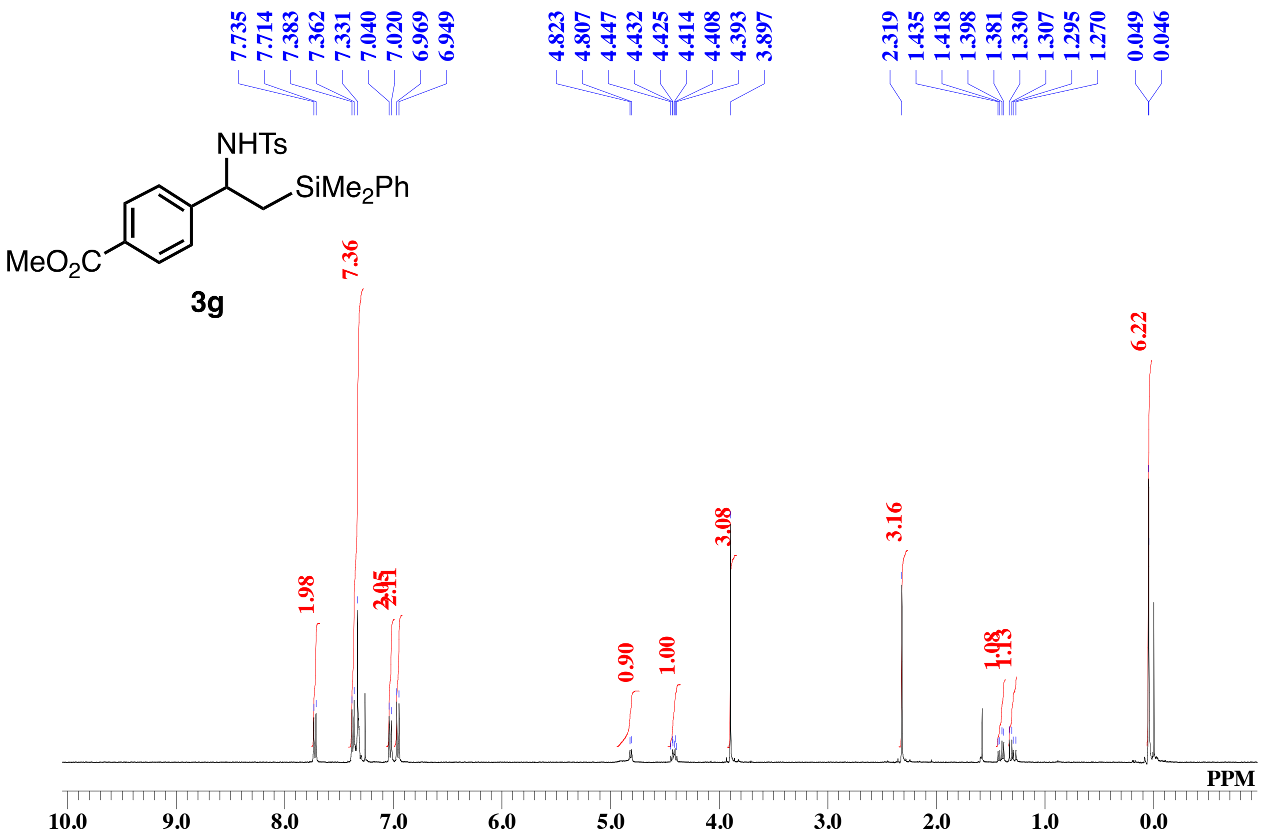

^{13}C NMR: (100 MHz, $CDCl_3$)

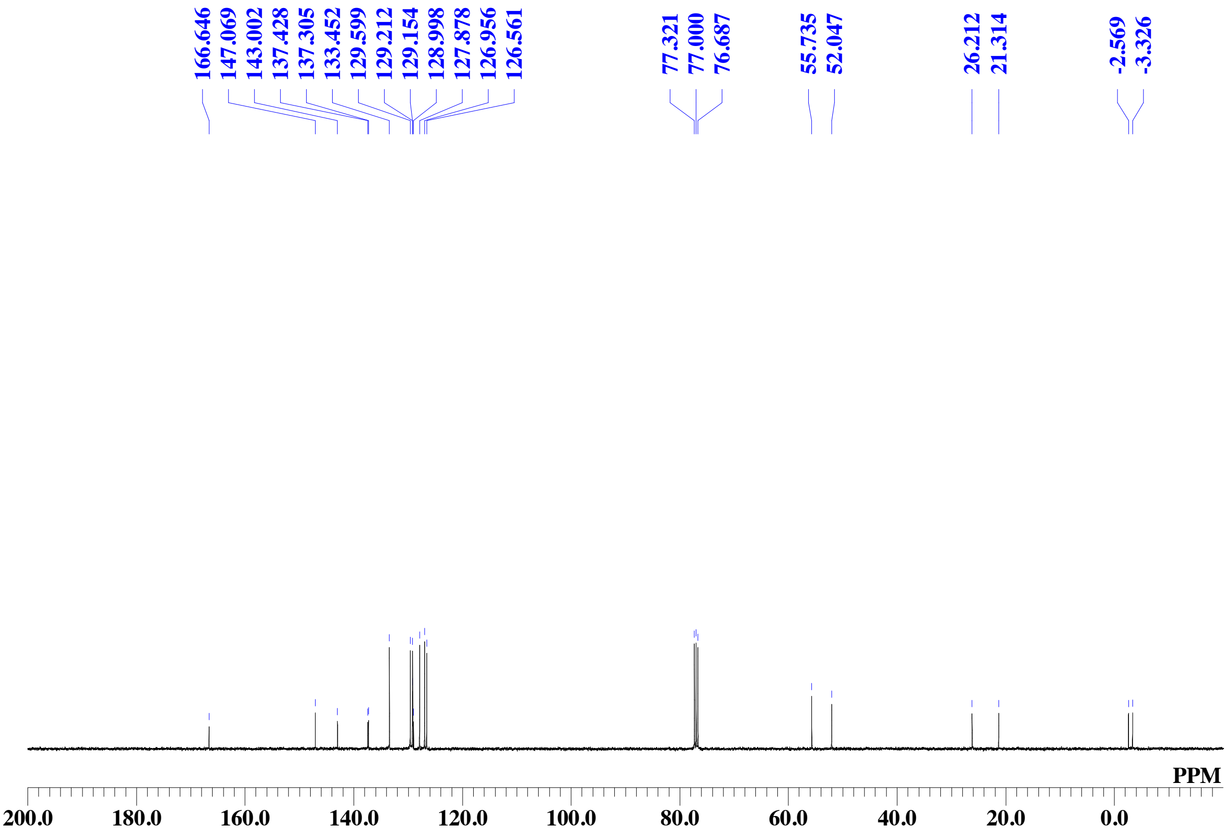

^{1}H NMR: (400 MHz, $CDCl_3$)

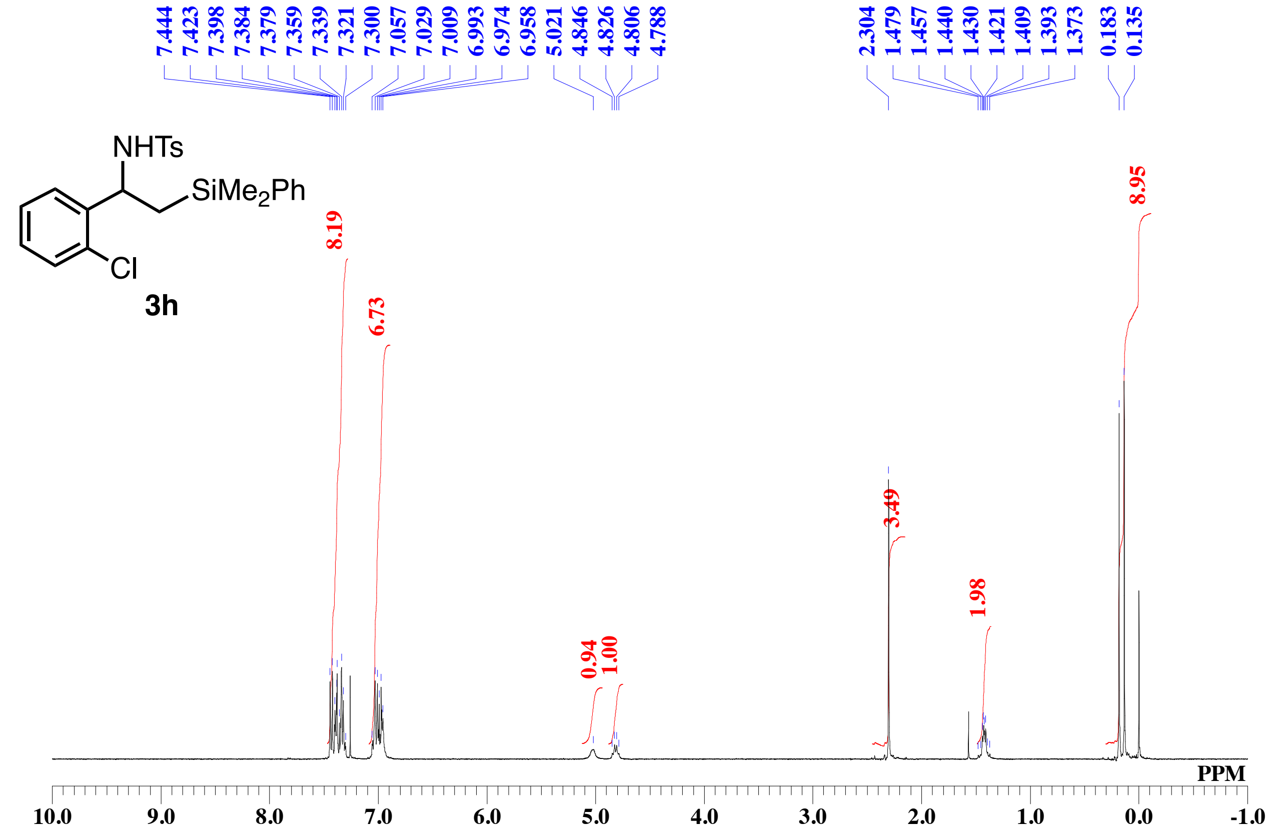

^{13}C NMR: (100 MHz, $CDCl_3$)

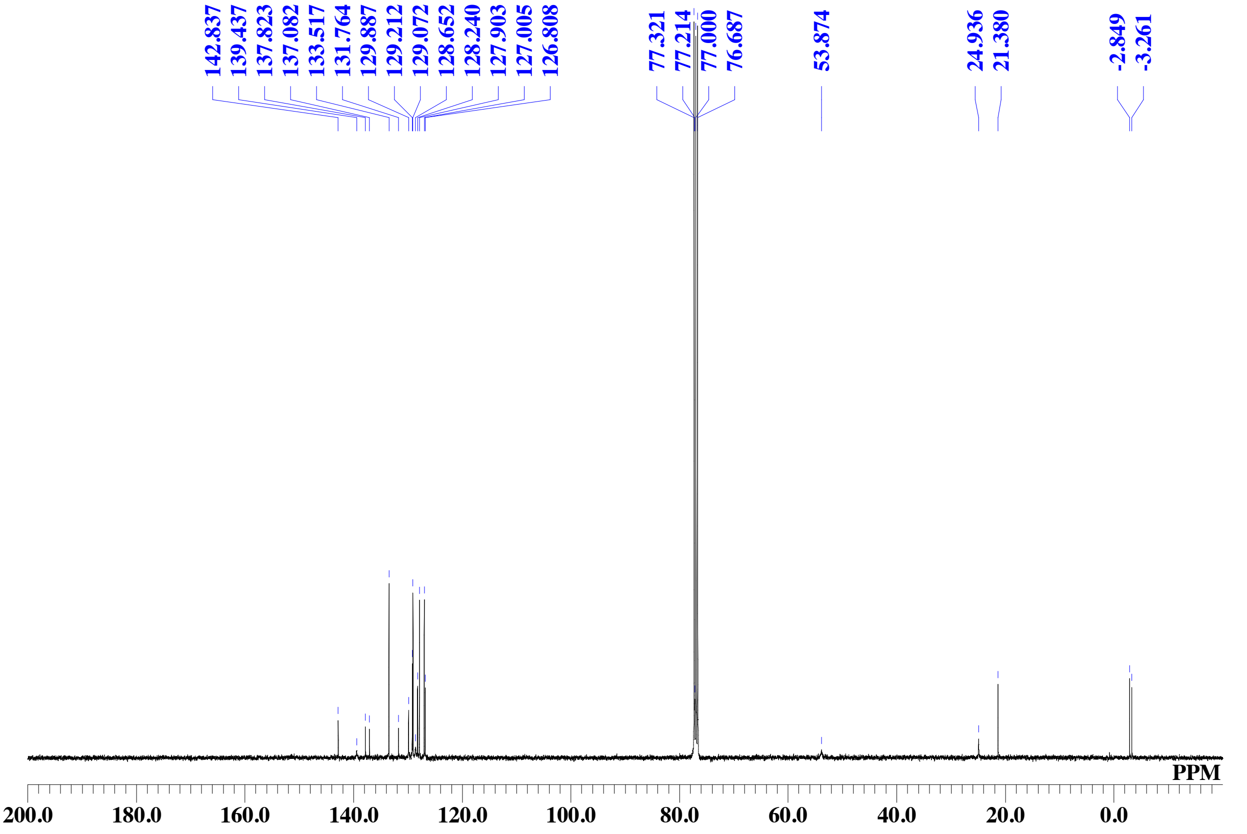

^{1}H NMR: (400 MHz, $CDCl_3$)

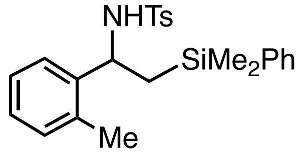

3i

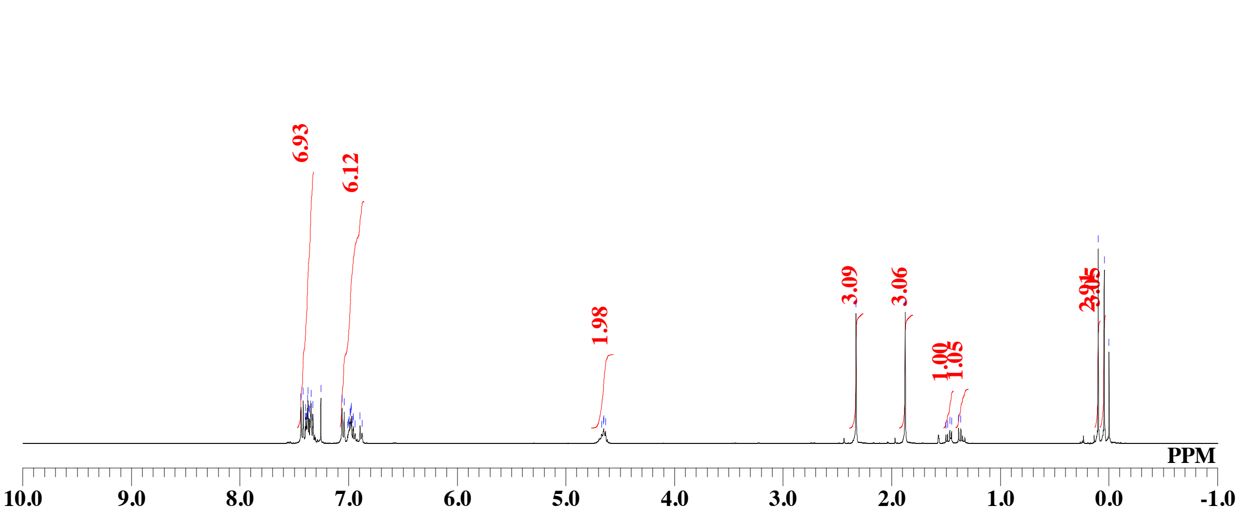

^{13}C NMR: (100 MHz, $CDCl_3$)

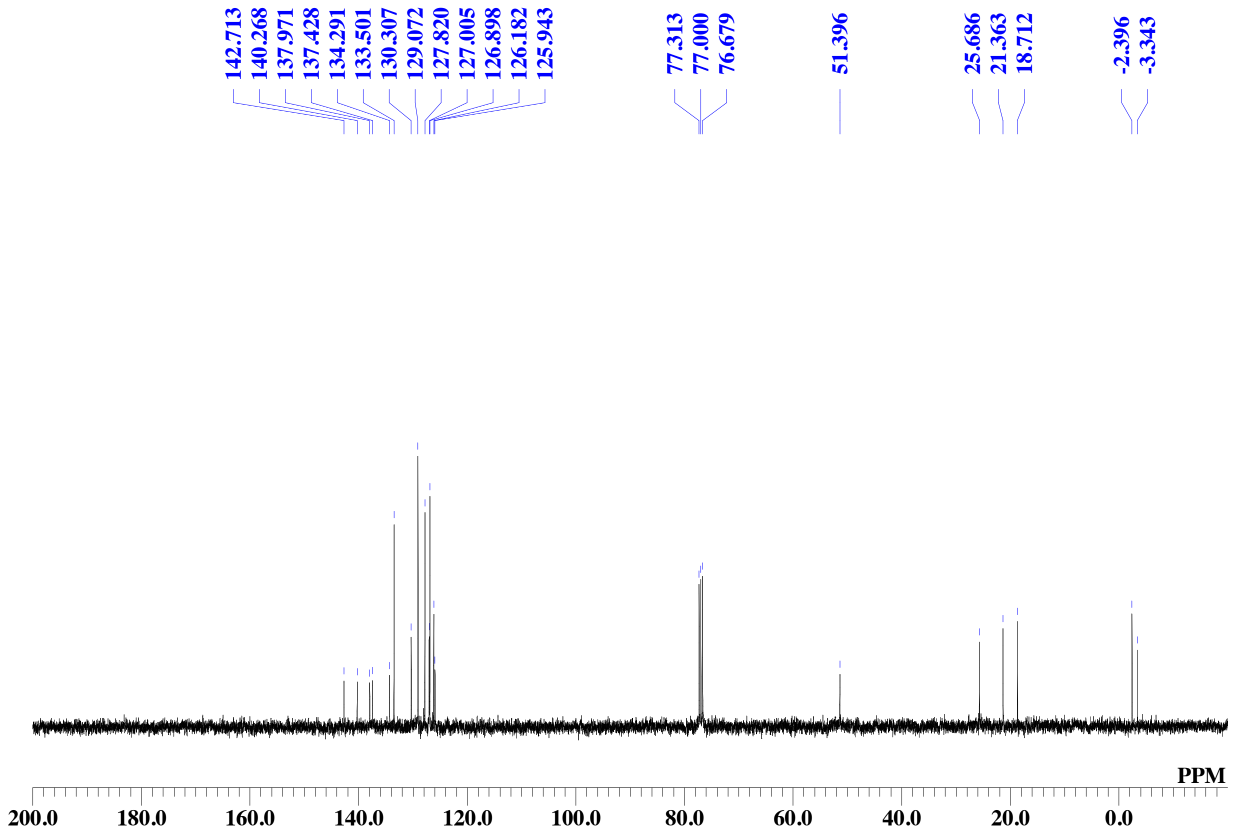

^{1}H NMR: (400 MHz, $CDCl_3$)

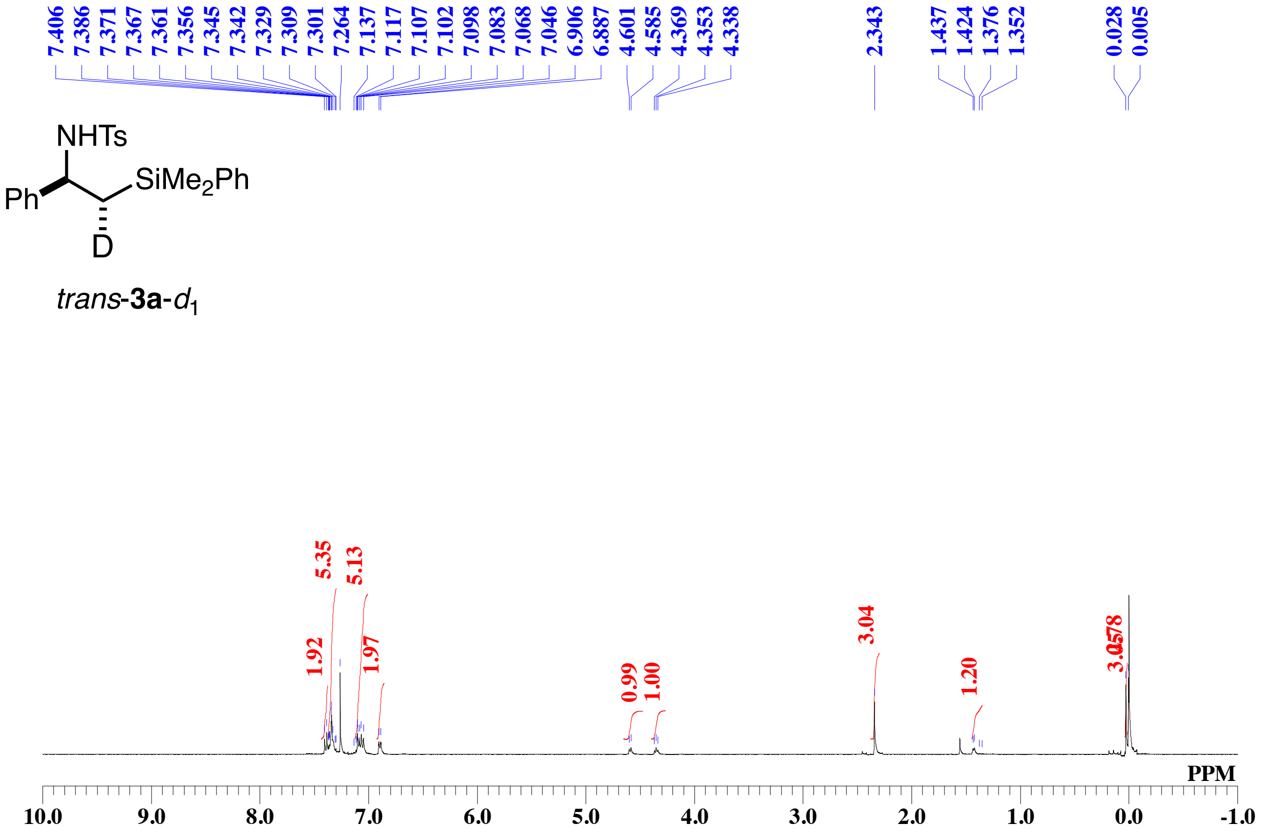

trans-**3a**-d_1

^{13}C NMR: (100 MHz, $CDCl_3$)

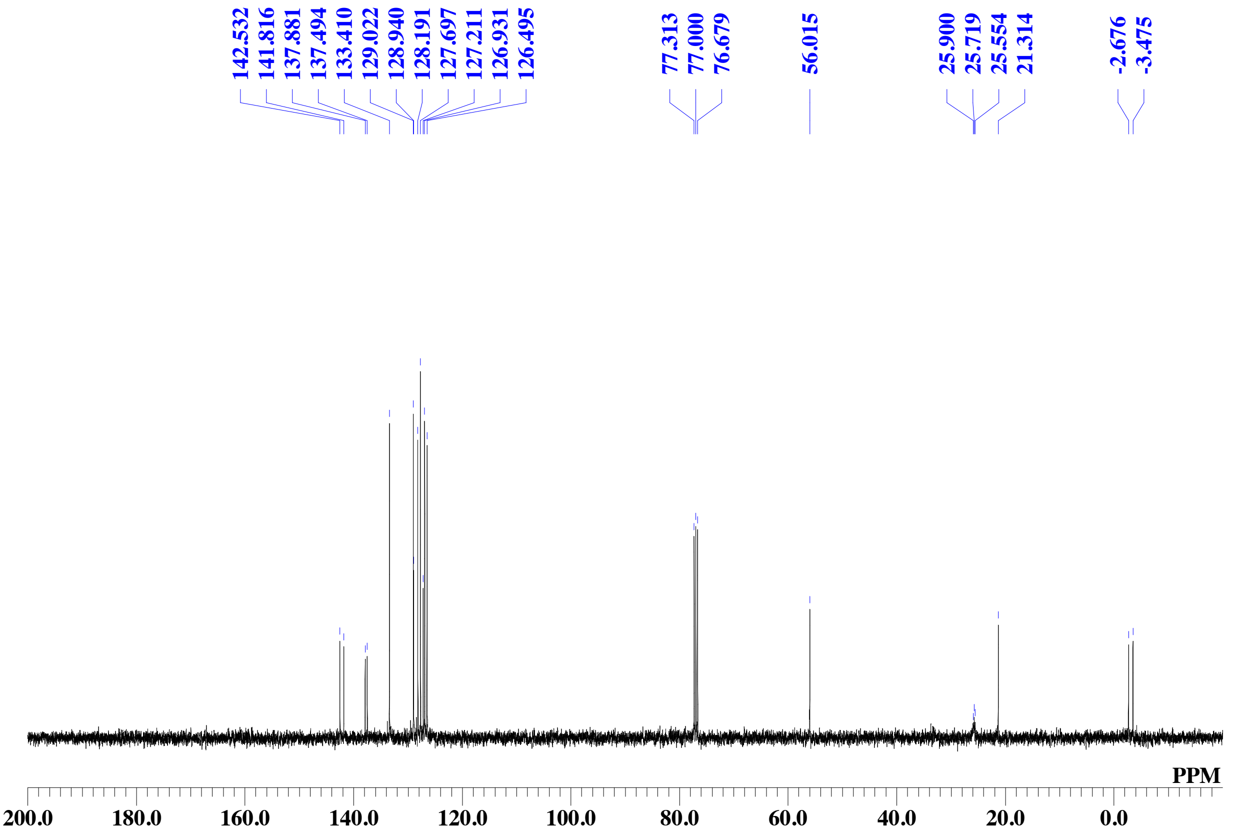

^{1}H NMR: (400 MHz, $CDCl_3$)

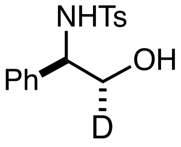

trans-**10**

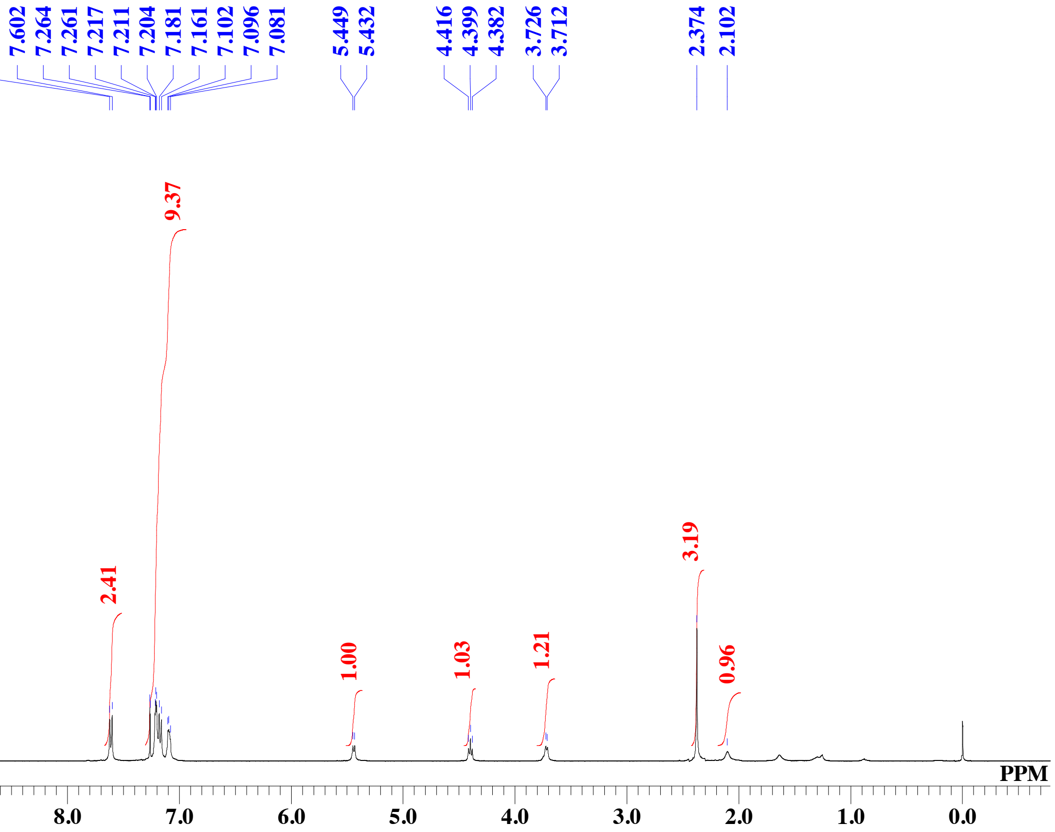

^{13}C NMR: (100 MHz, $CDCl_3$)

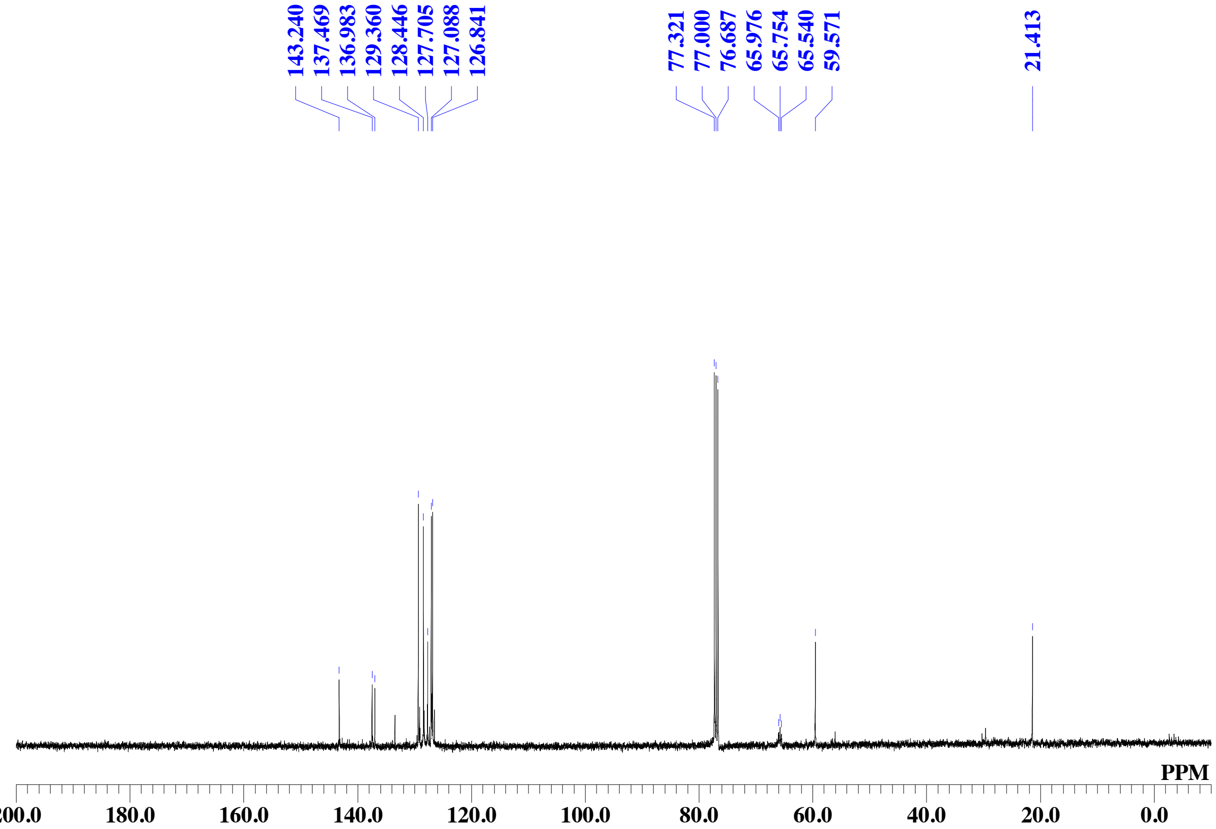

^{1}H NMR: (400 MHz, $CDCl_3$)

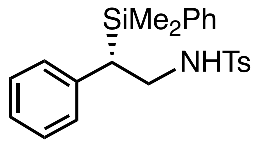

(*S*)-**8a**

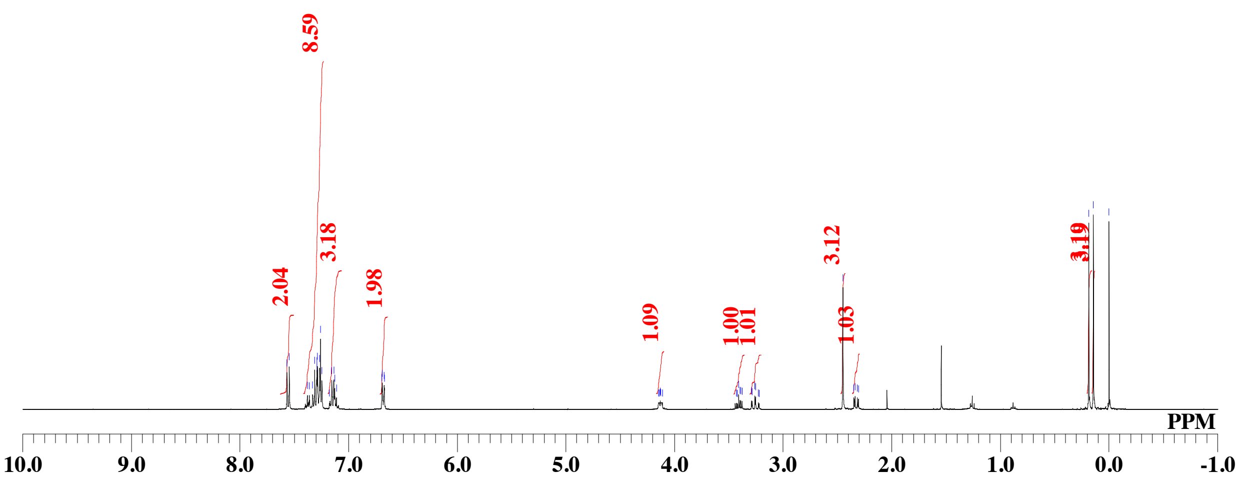

^{13}C NMR: (100 MHz, $CDCl_3$)

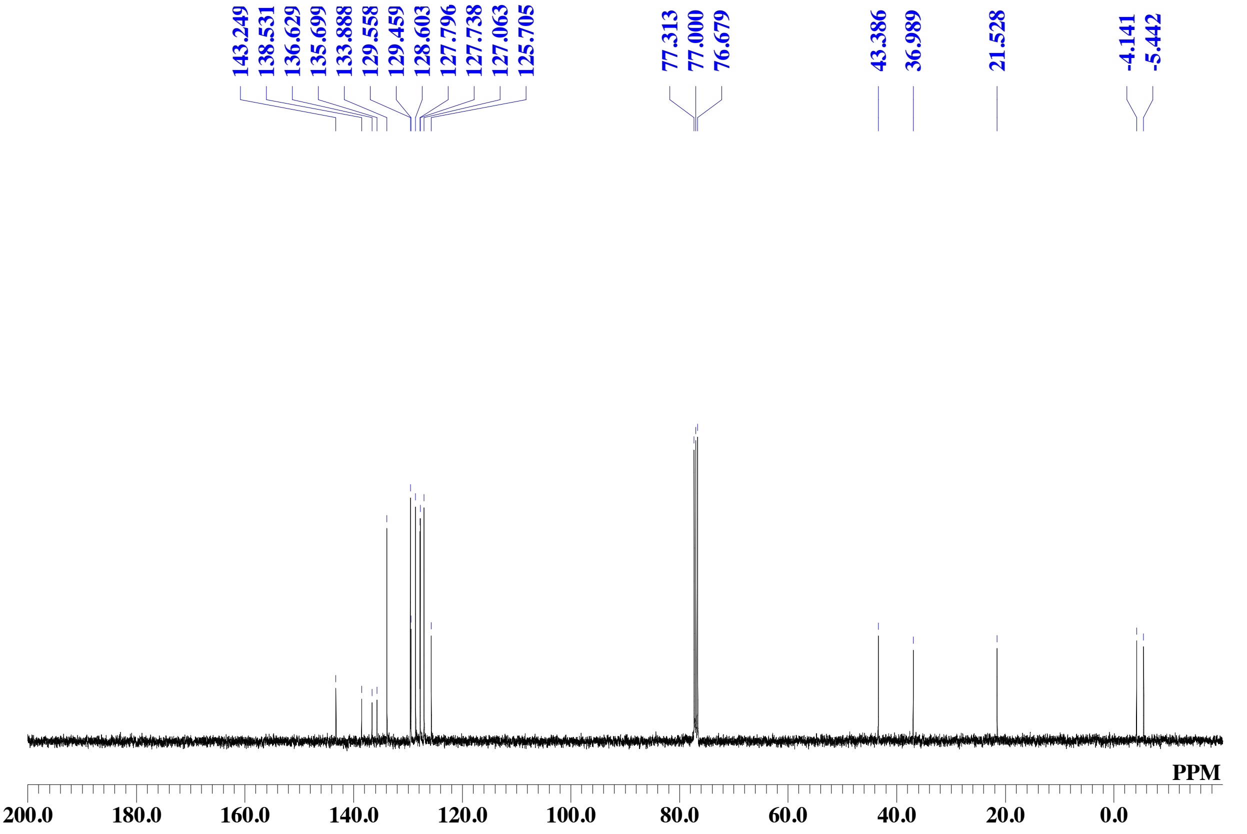

^{1}H NMR: (400 MHz, $CDCl_3$)

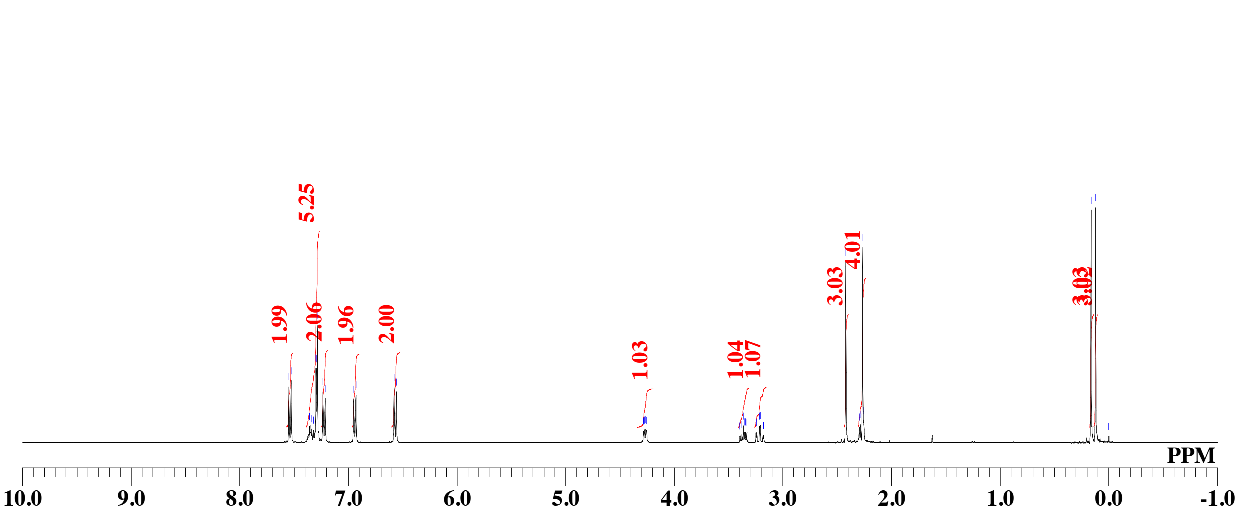

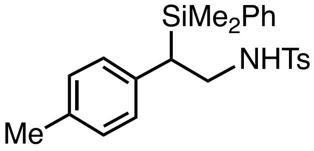

8b

^{13}C NMR: (100 MHz, $CDCl_3$)

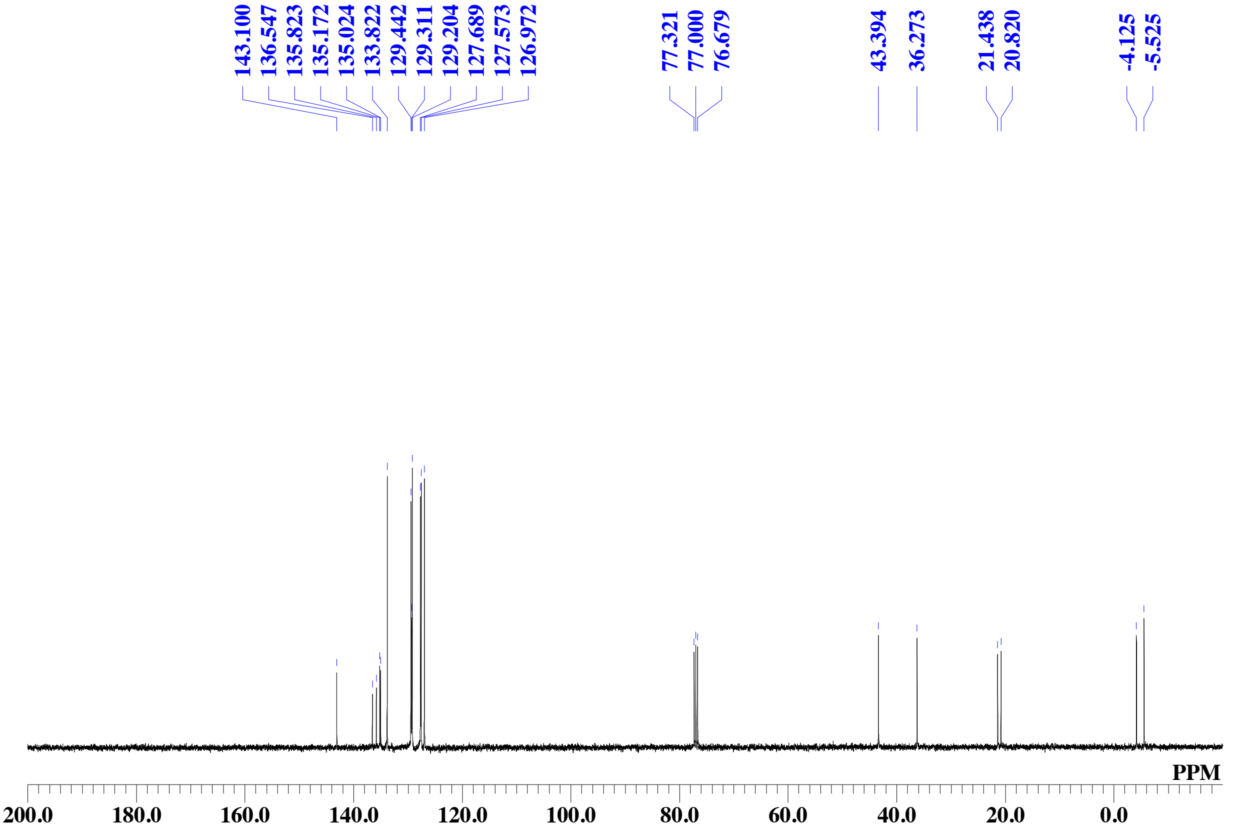

^{1}H NMR: (400 MHz, $CDCl_3$)

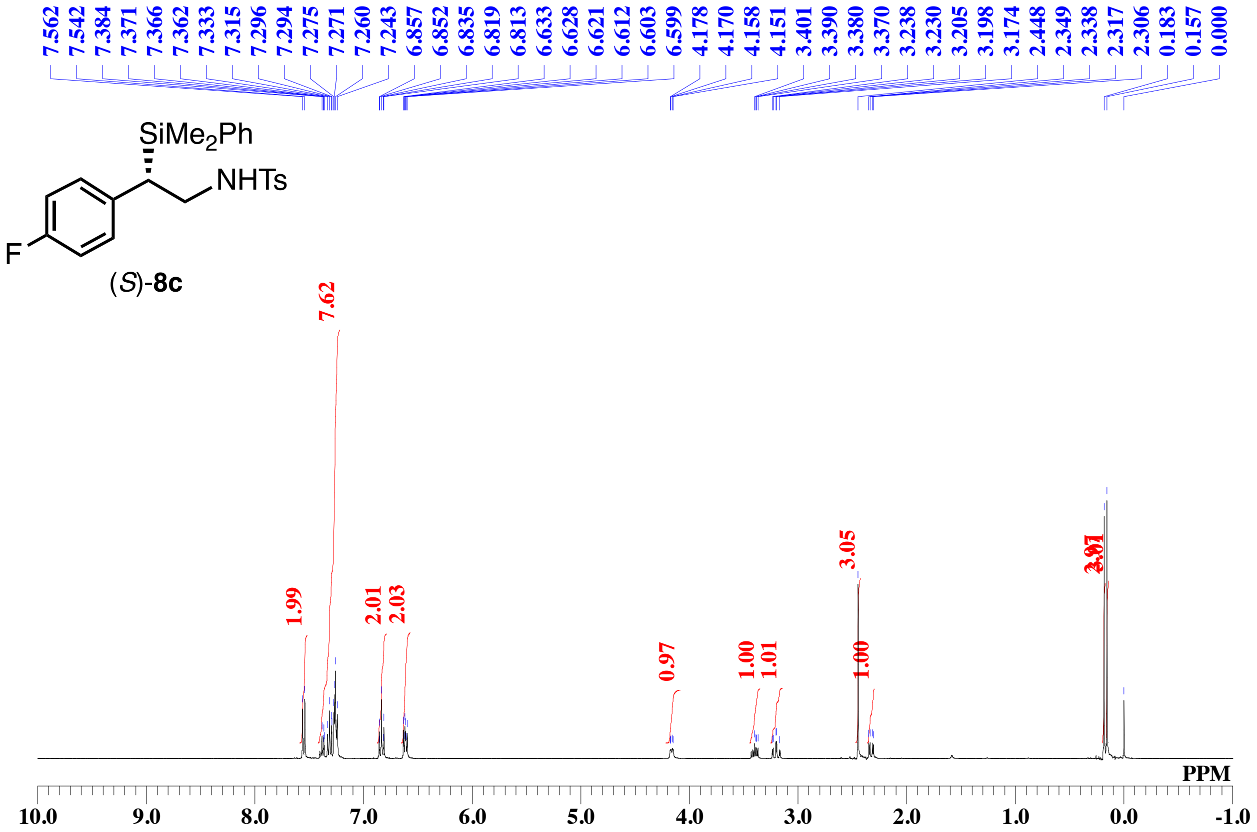

^{13}C NMR: (100 MHz, $CDCl_3$)

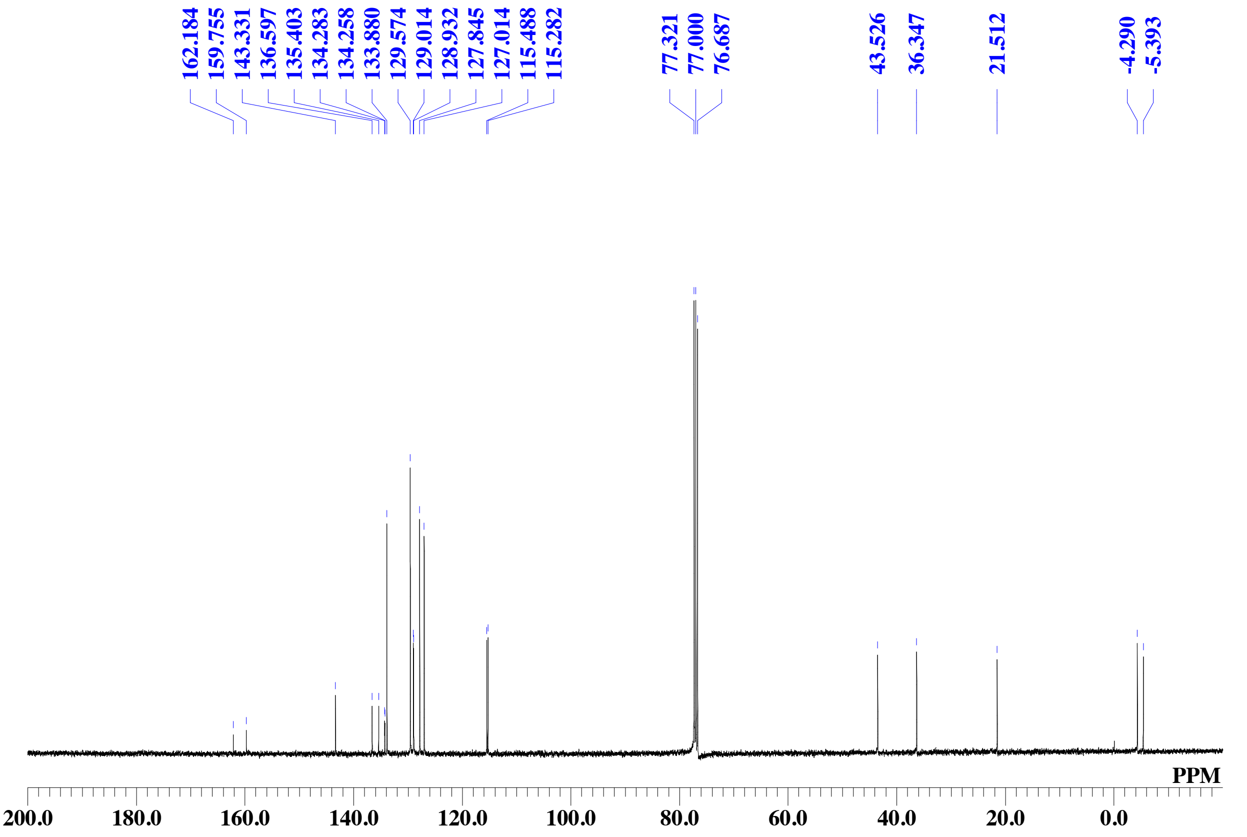

^{1}H NMR: (400 MHz, $CDCl_3$)

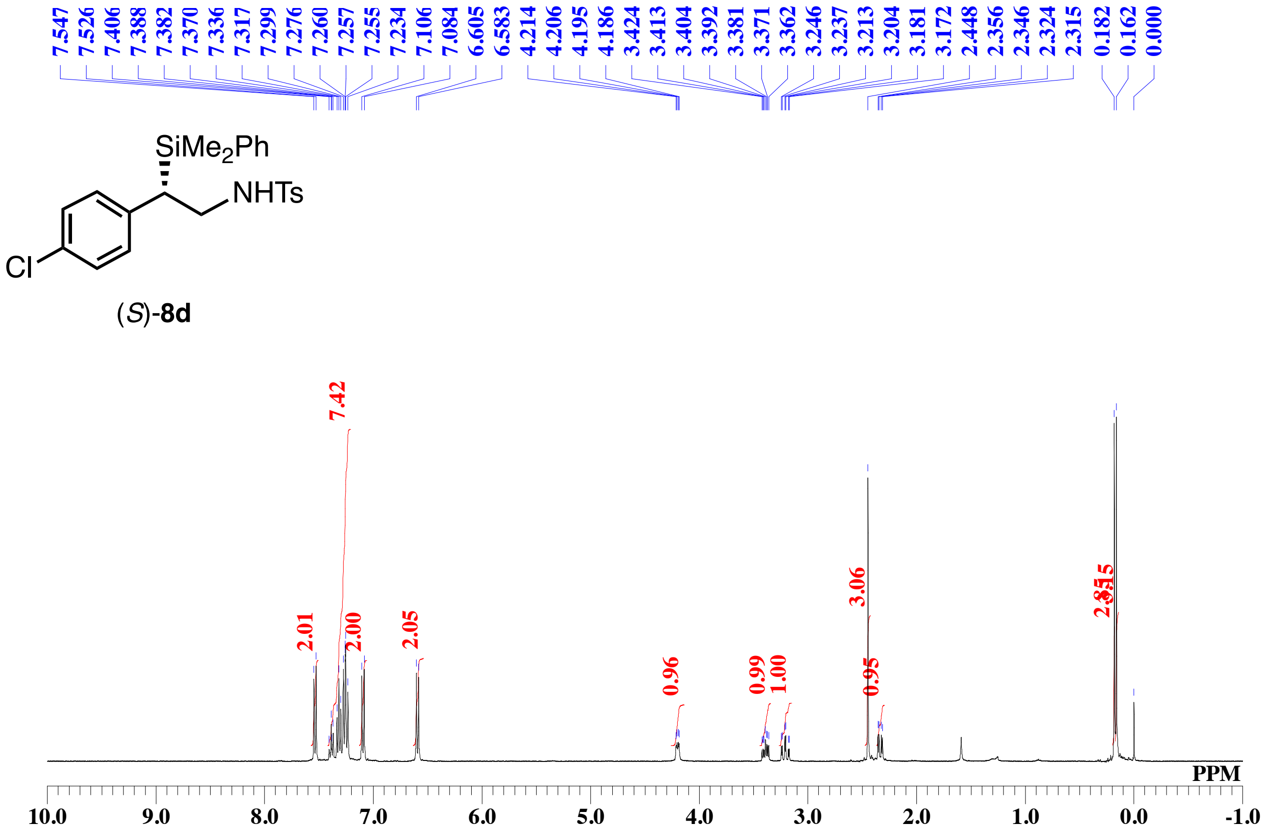

^{13}C NMR: (100 MHz, $CDCl_3$)

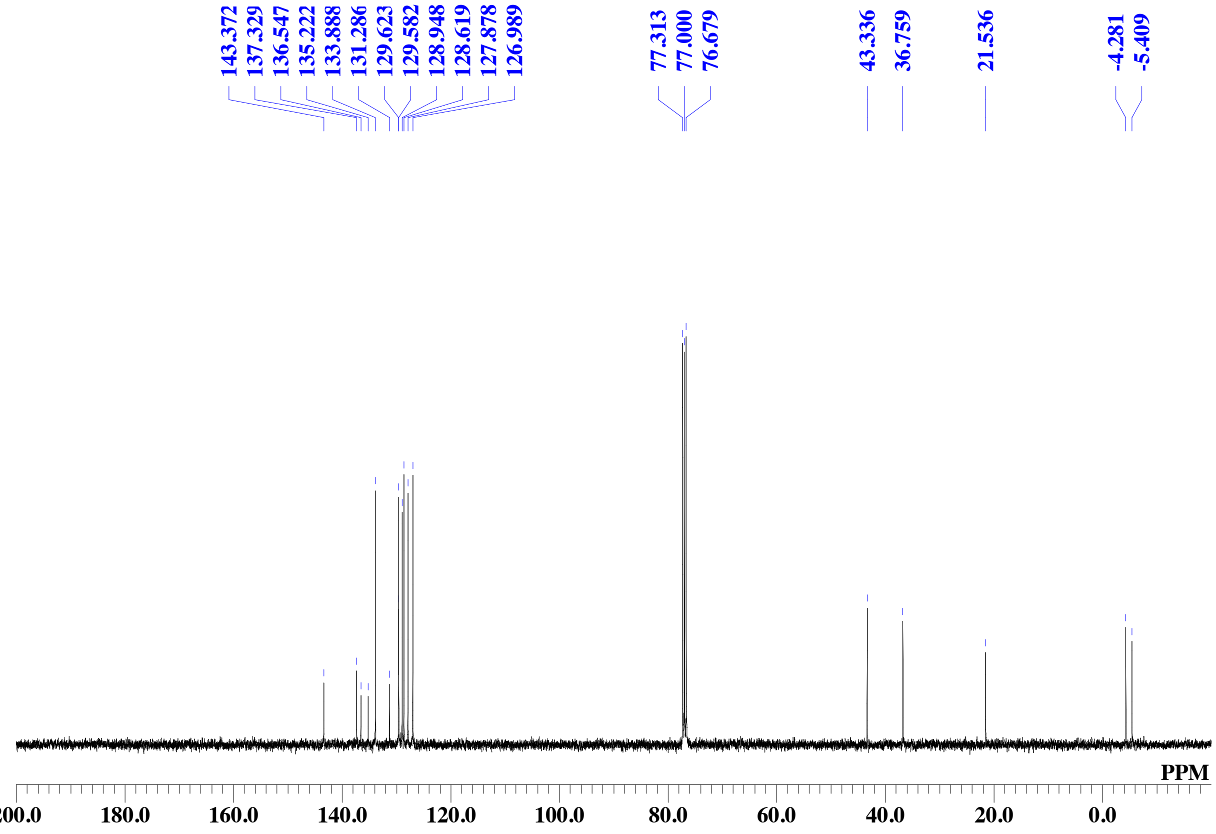

^{1}H NMR: (400 MHz, $CDCl_3$)

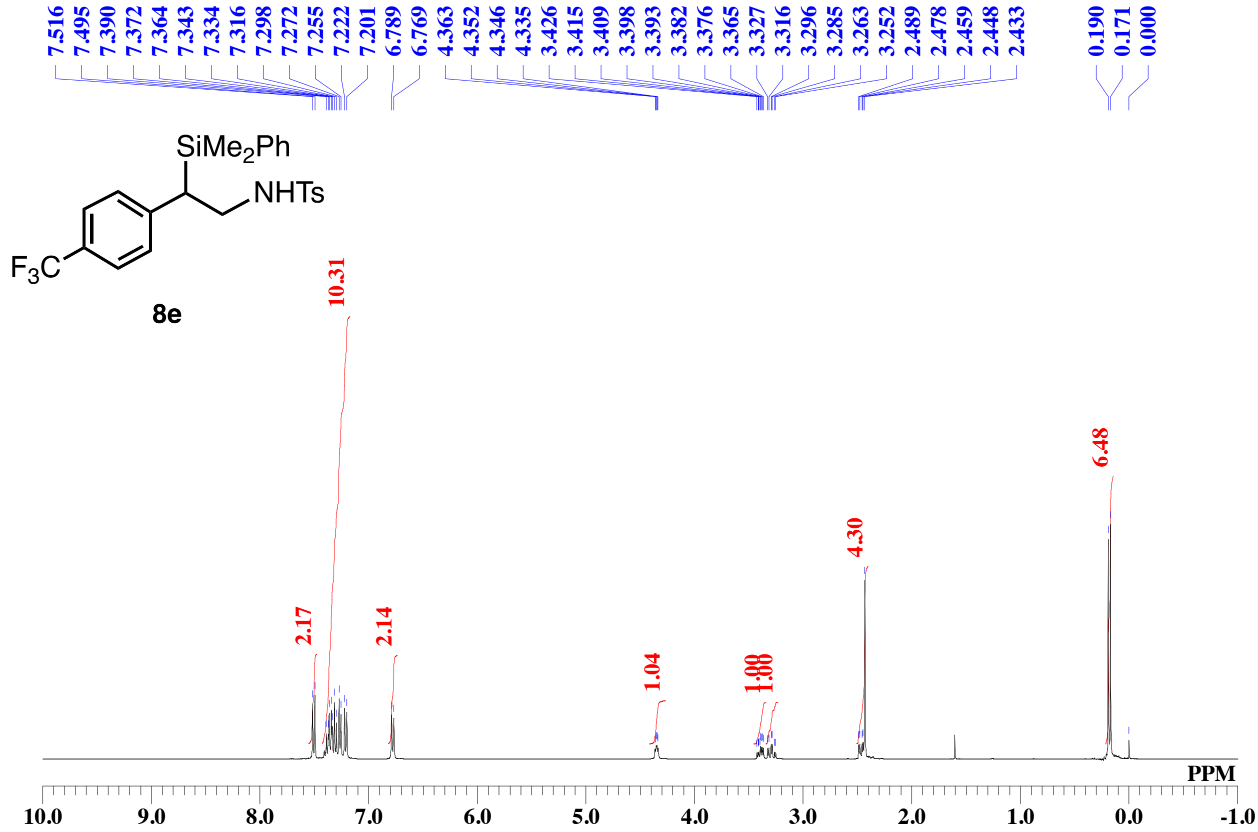

^{13}C NMR: (100 MHz, $CDCl_3$)

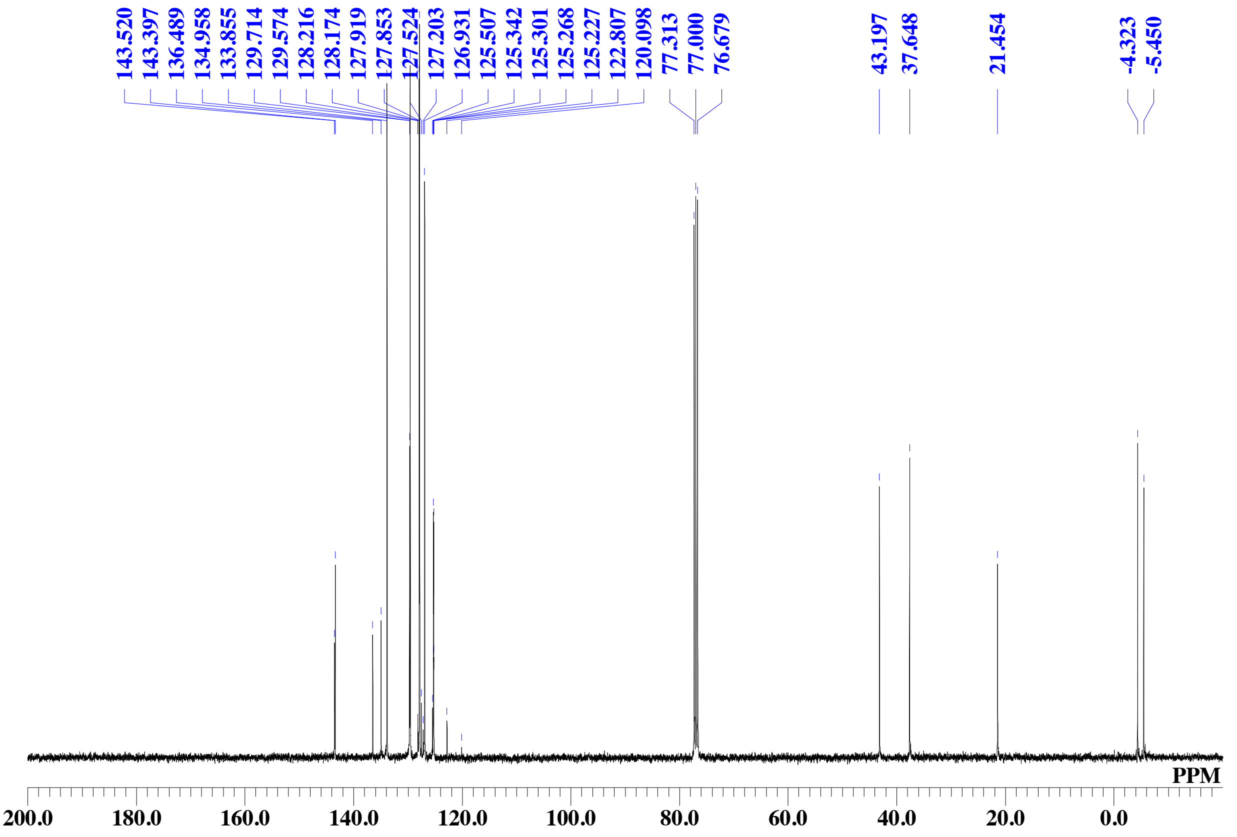

^{1}H NMR: (400 MHz, $CDCl_3$)

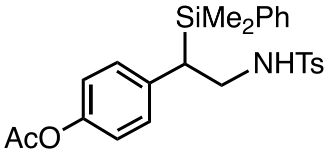

8f

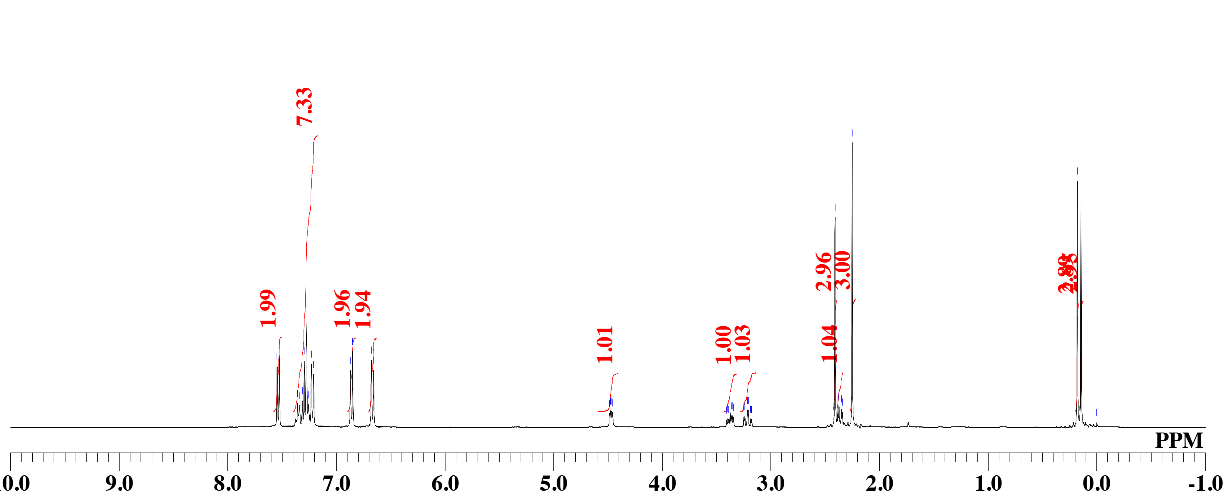

^{13}C NMR: (100 MHz, $CDCl_3$)

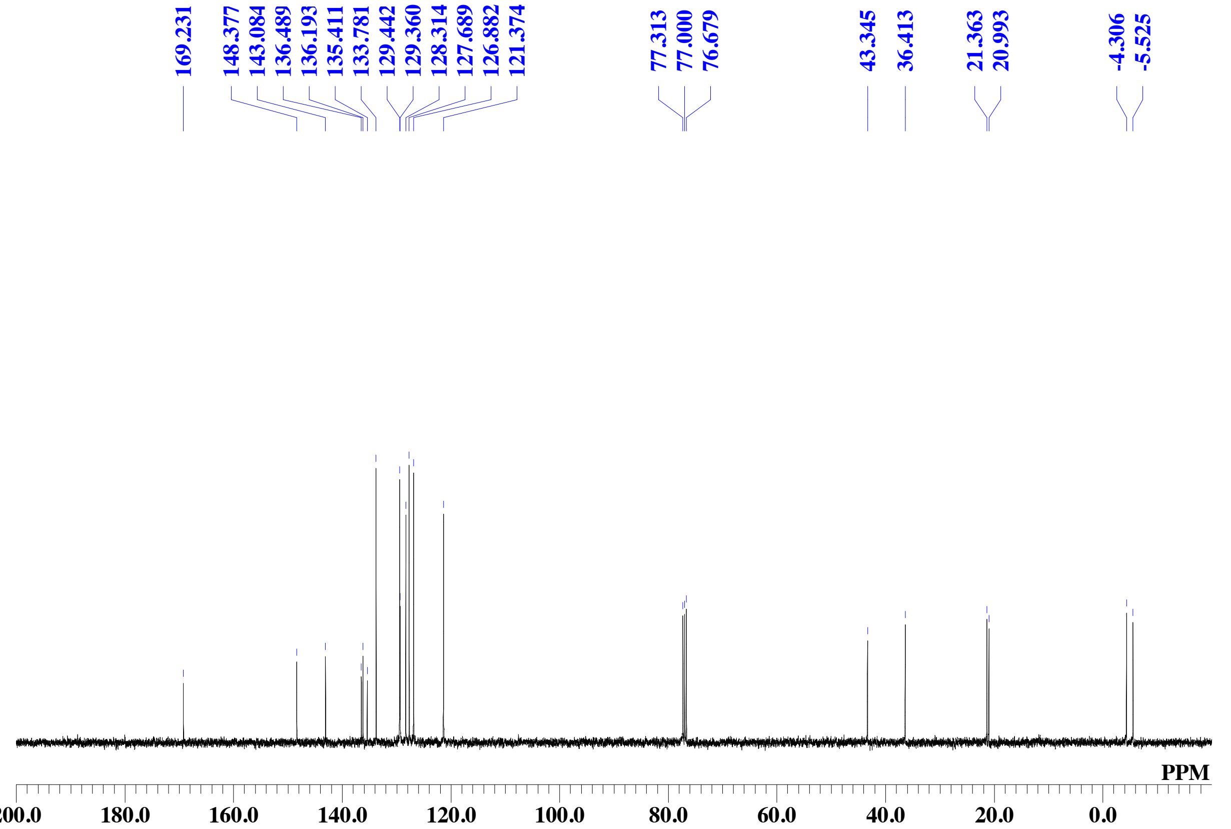

^1H NMR: (400 MHz, $CDCl_3$)

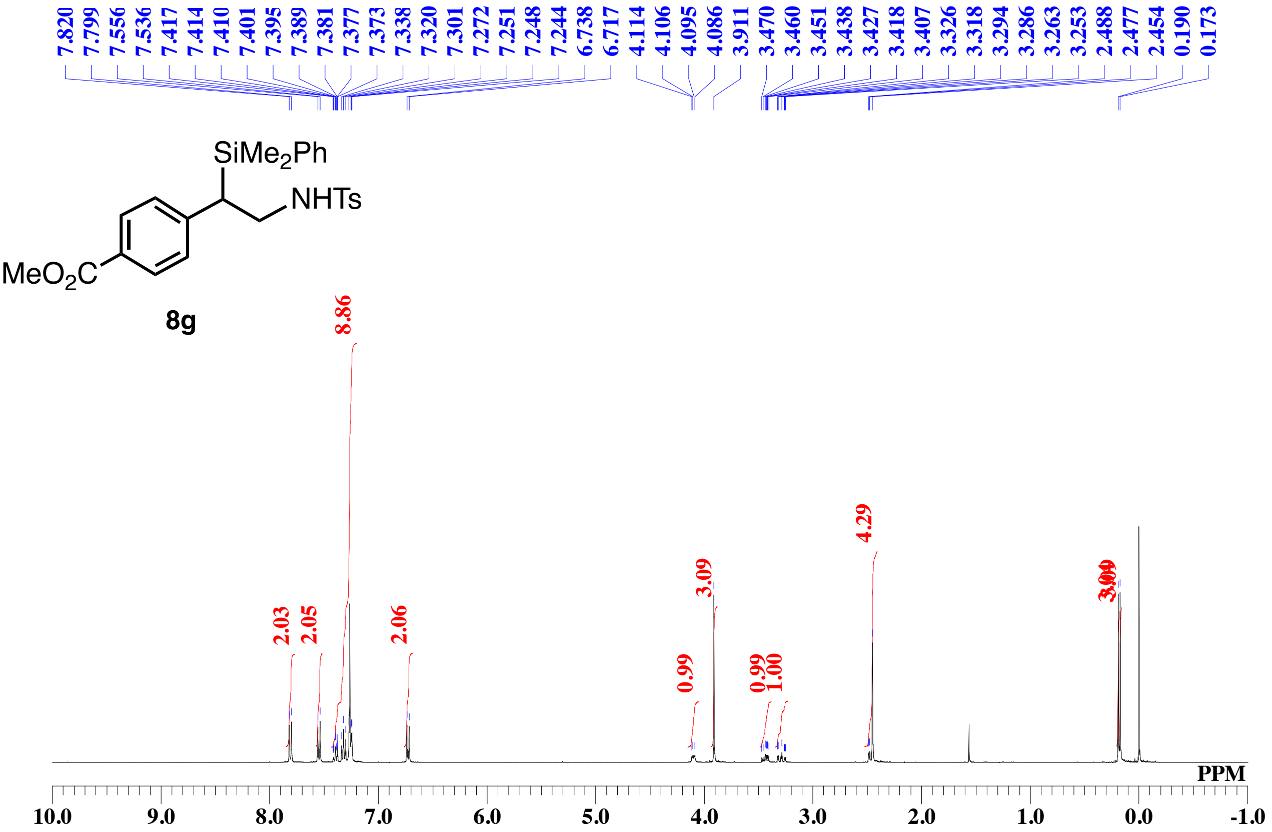

8g

^{13}C NMR: (100 MHz, $CDCl_3$)

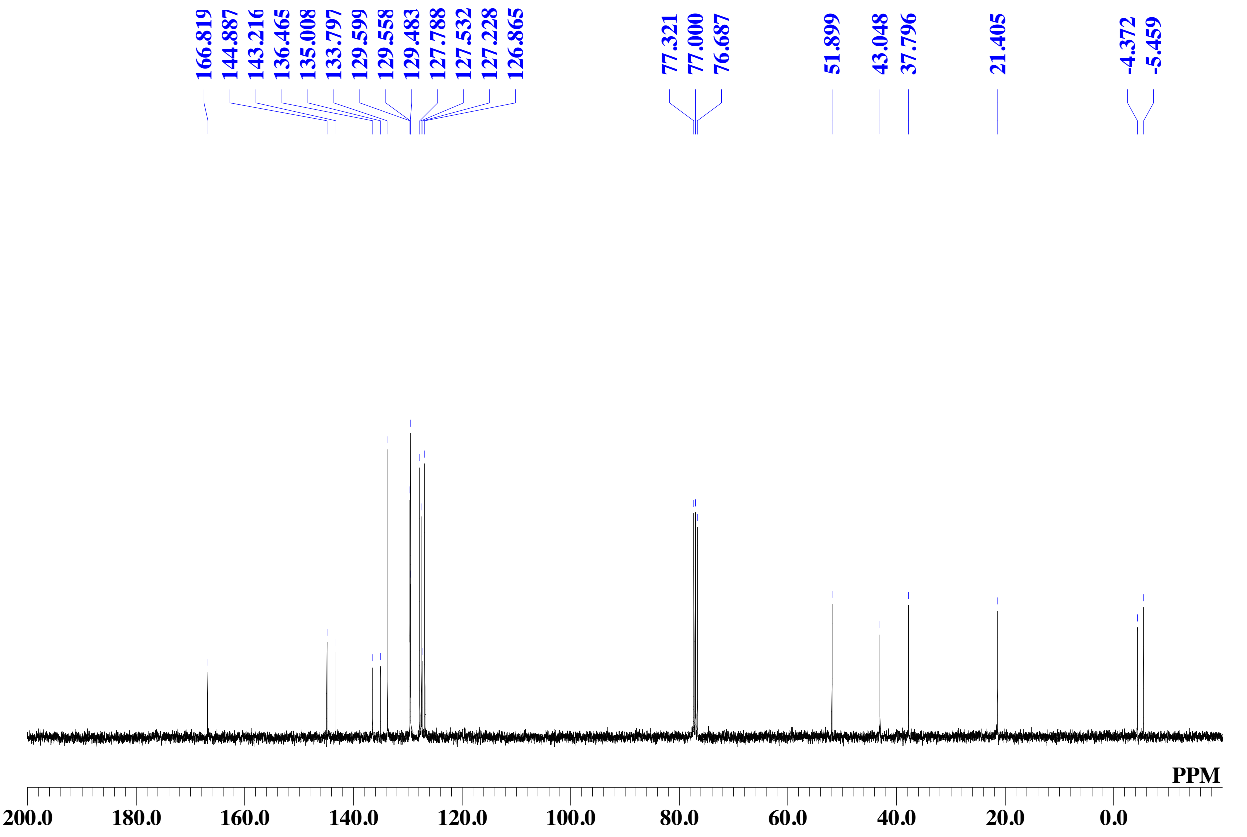

^{1}H NMR: (400 MHz, $CDCl_3$)

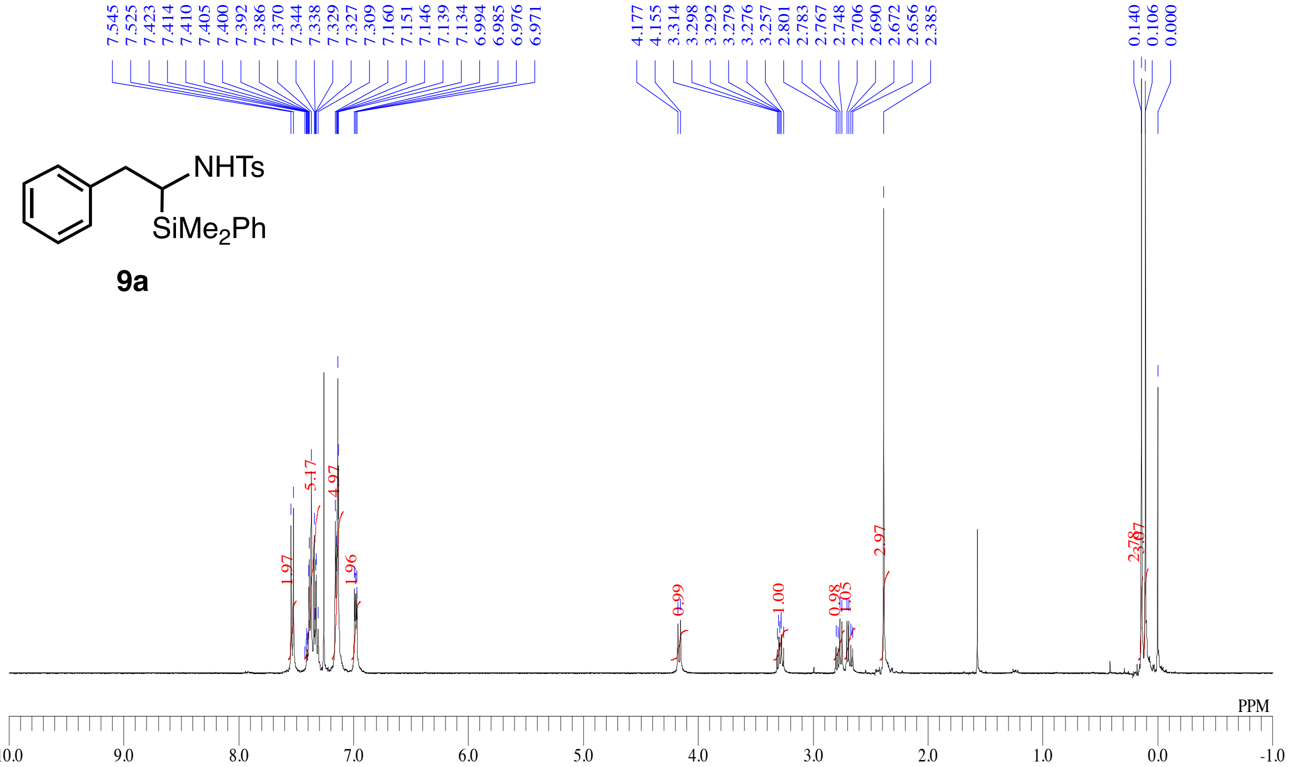

^{13}C NMR: (100 MHz, $CDCl_3$)

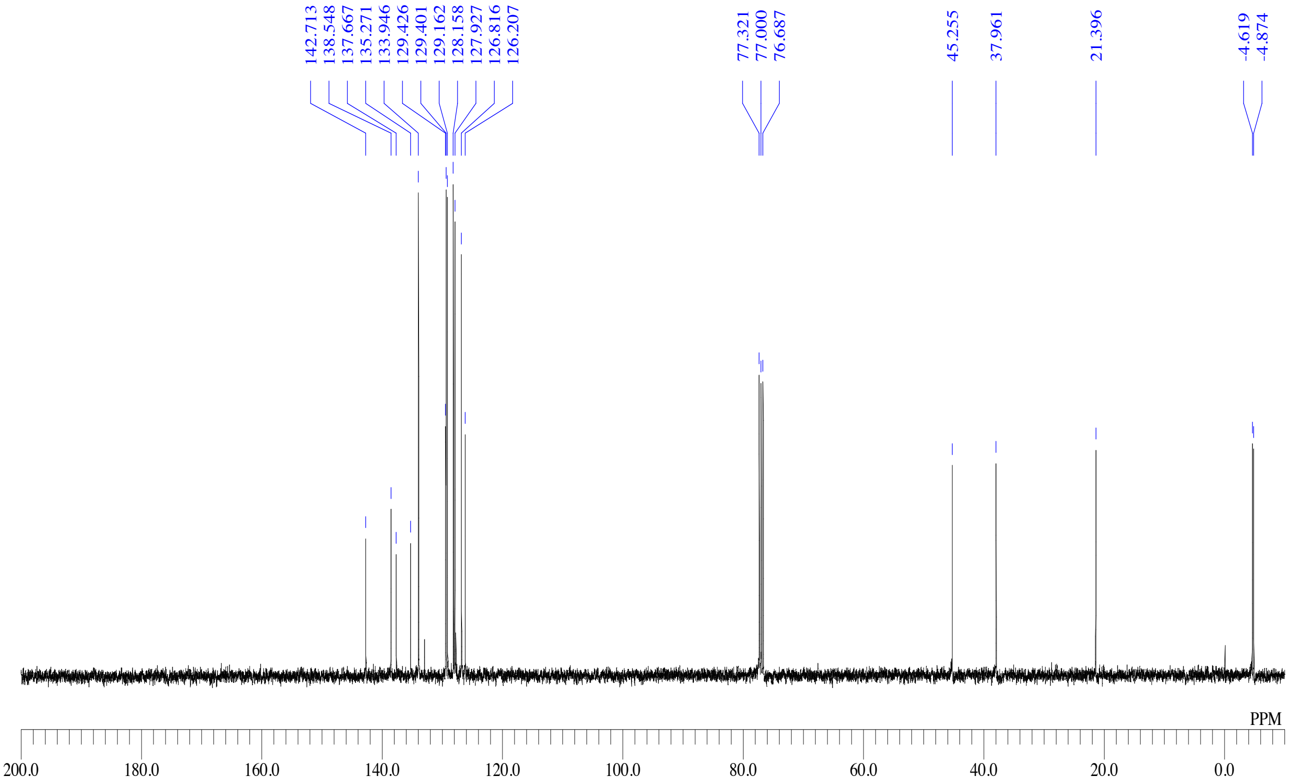

^{1}H NMR: (400 MHz, $CDCl_3$)

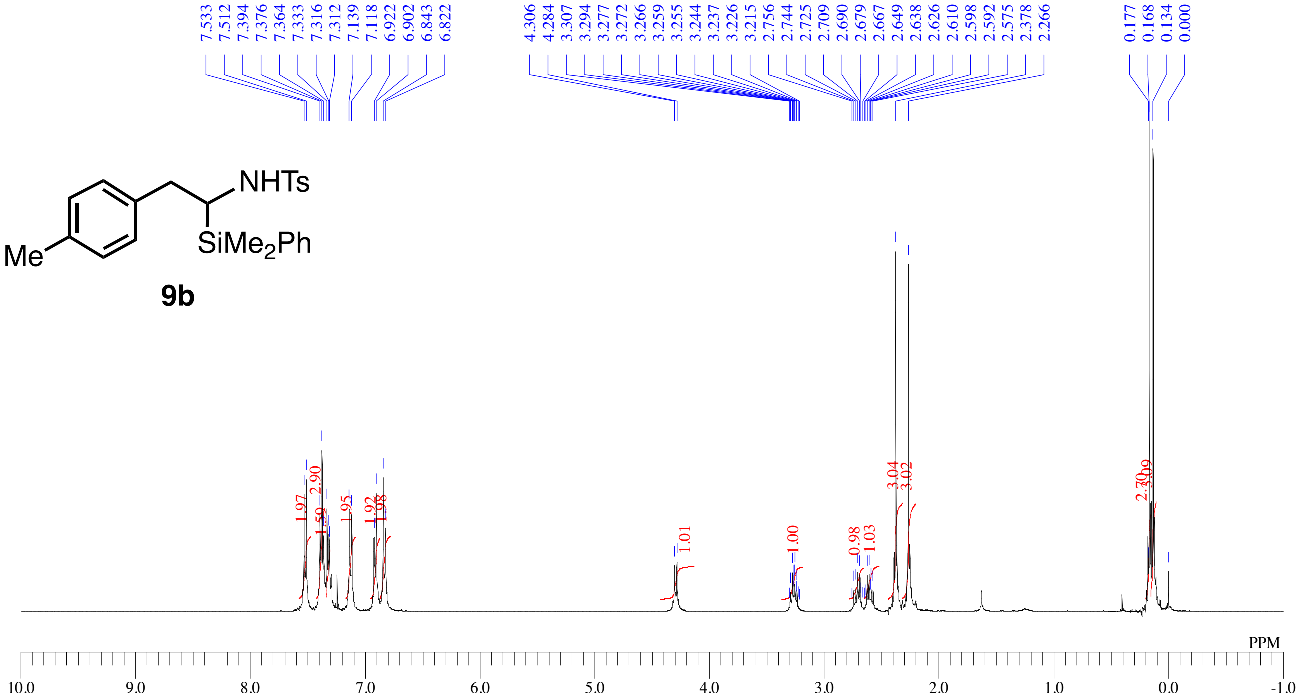

9b

^{13}C NMR: (100 MHz, $CDCl_3$)

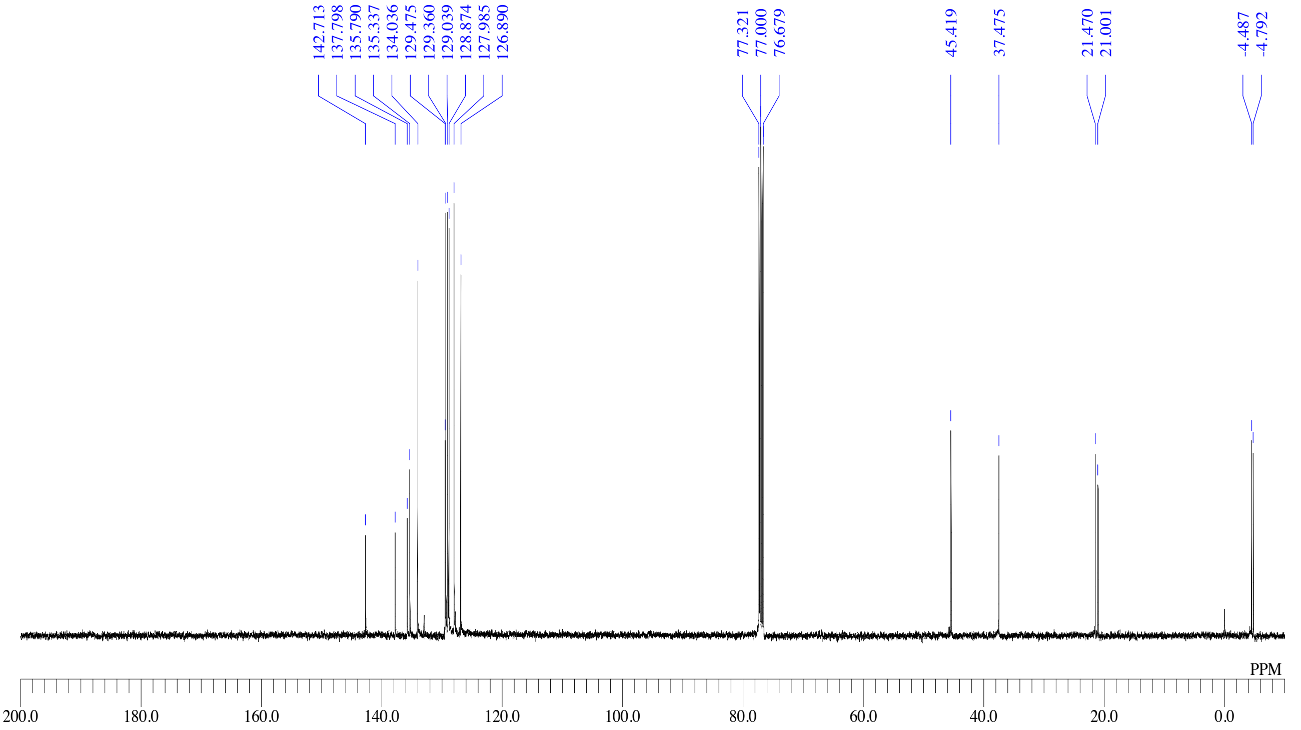

^{1}H NMR: (400 MHz, $CDCl_3$)

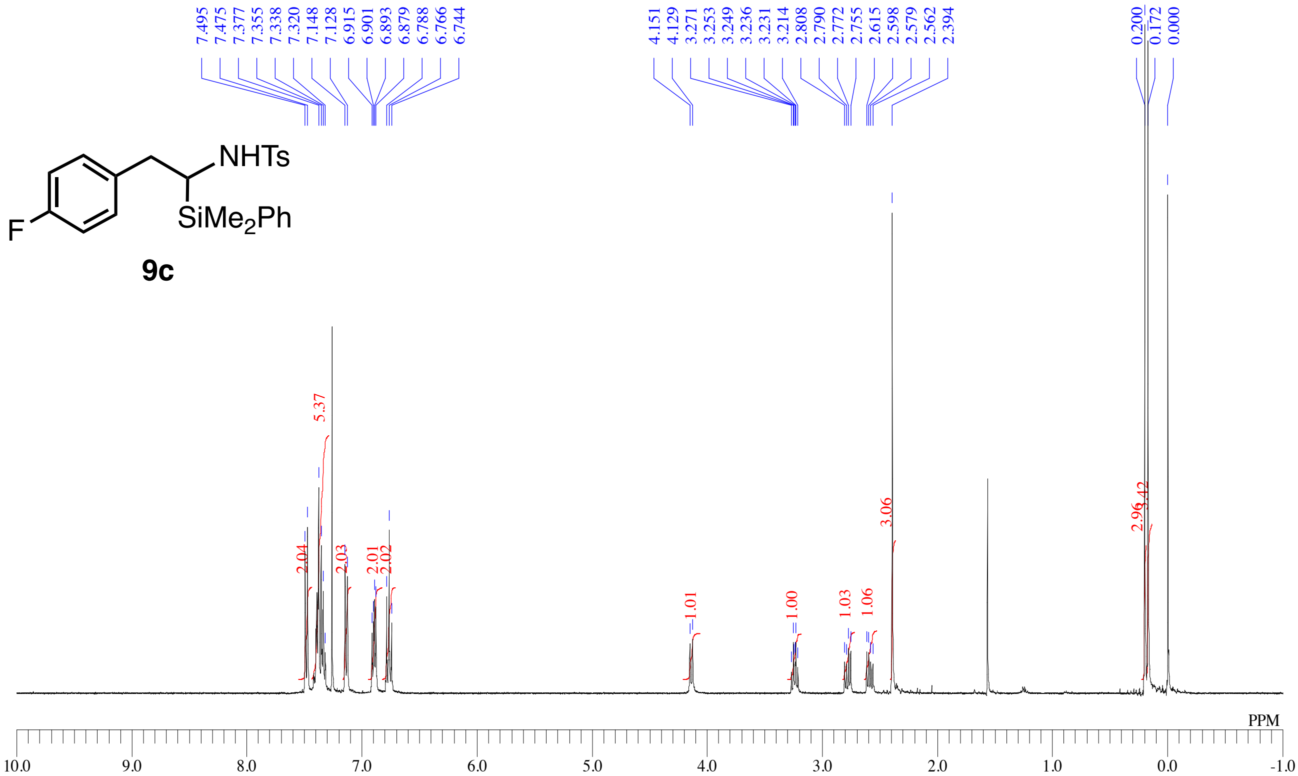

9c

^{13}C NMR: (100 MHz, $CDCl_3$)

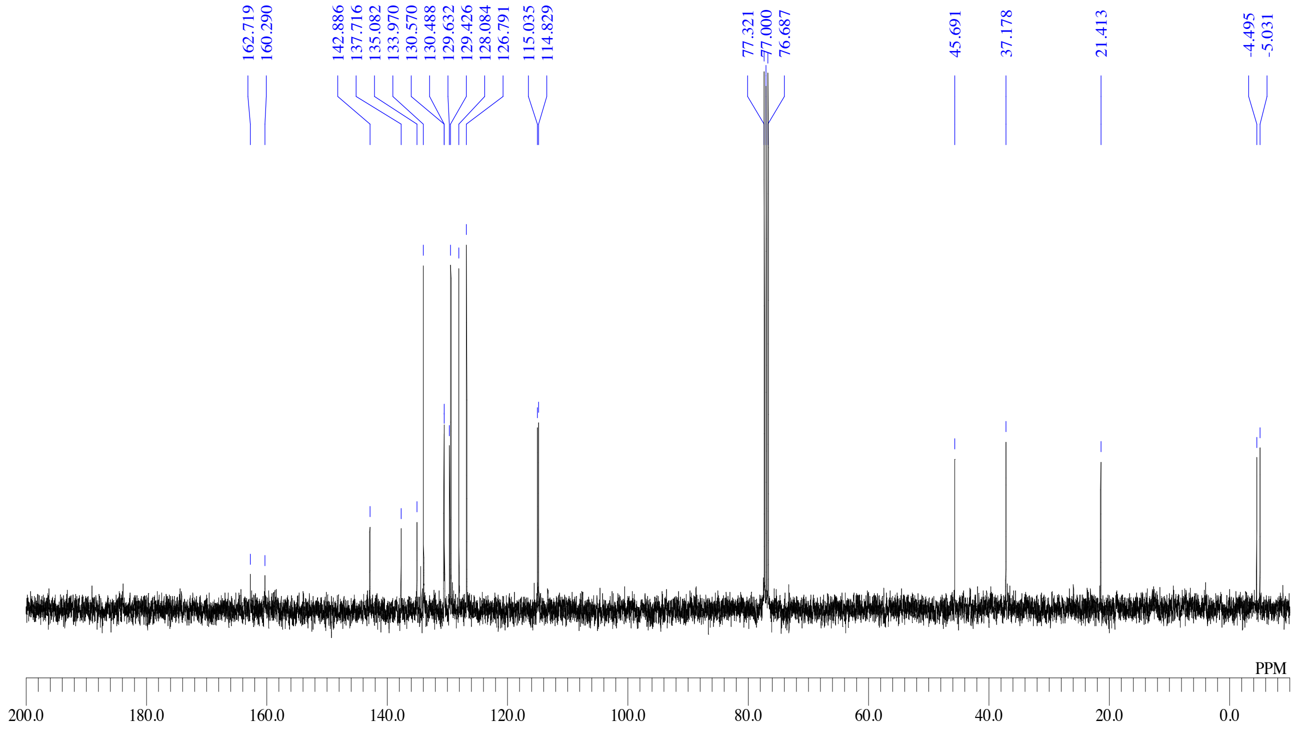

^{1}H NMR: (400 MHz, $CDCl_3$)

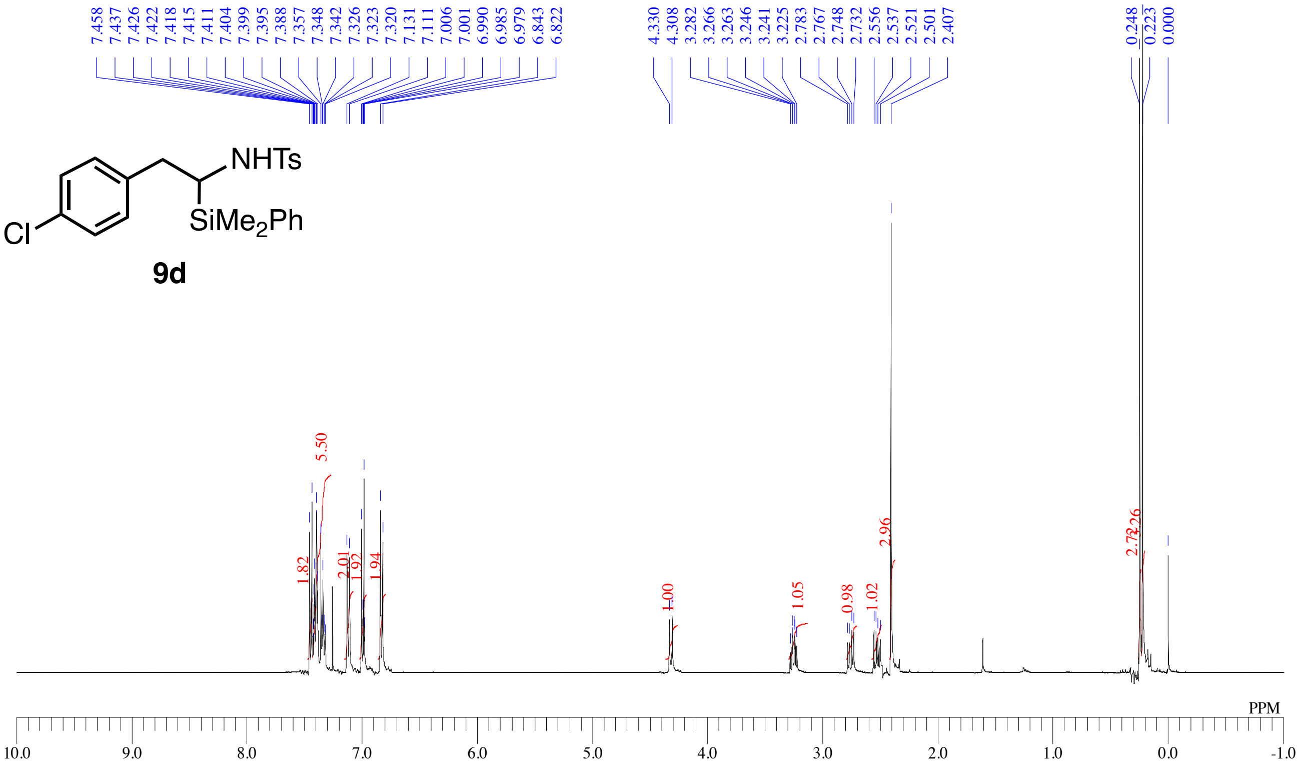

^{13}C NMR: (100 MHz, $CDCl_3$)

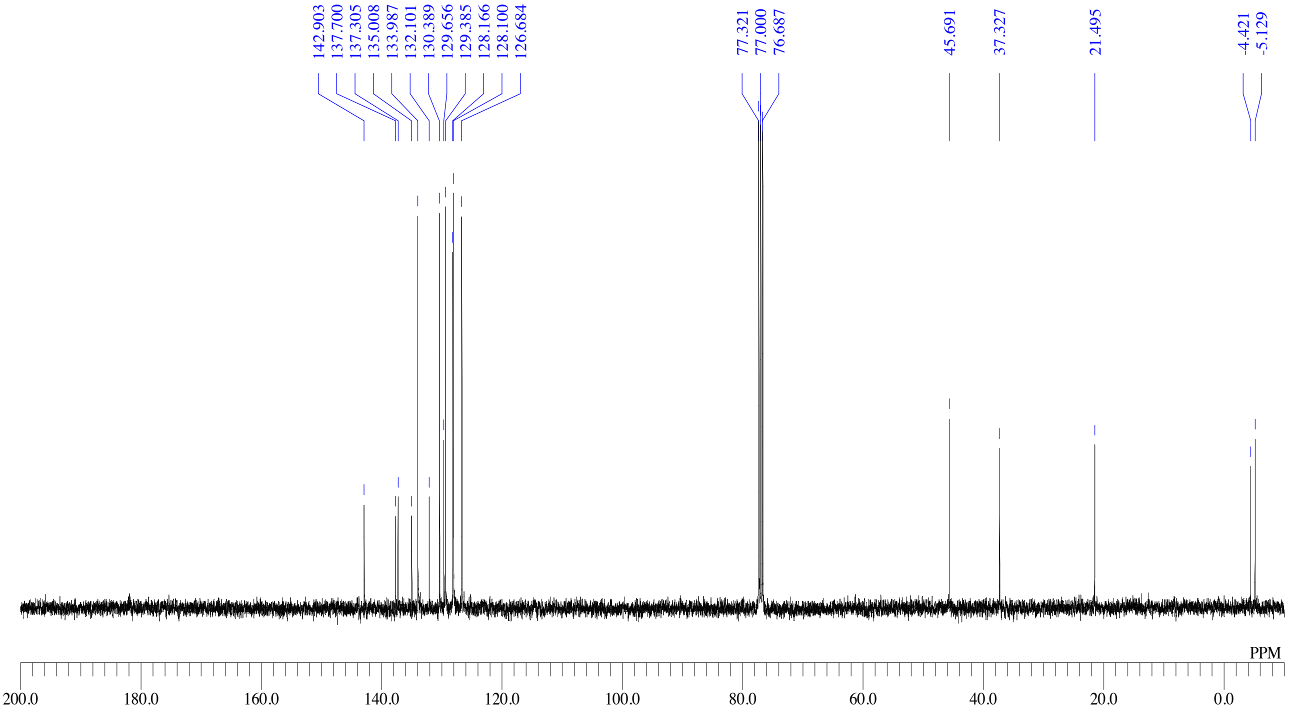

^{1}H NMR: (400 MHz, $CDCl_3$)

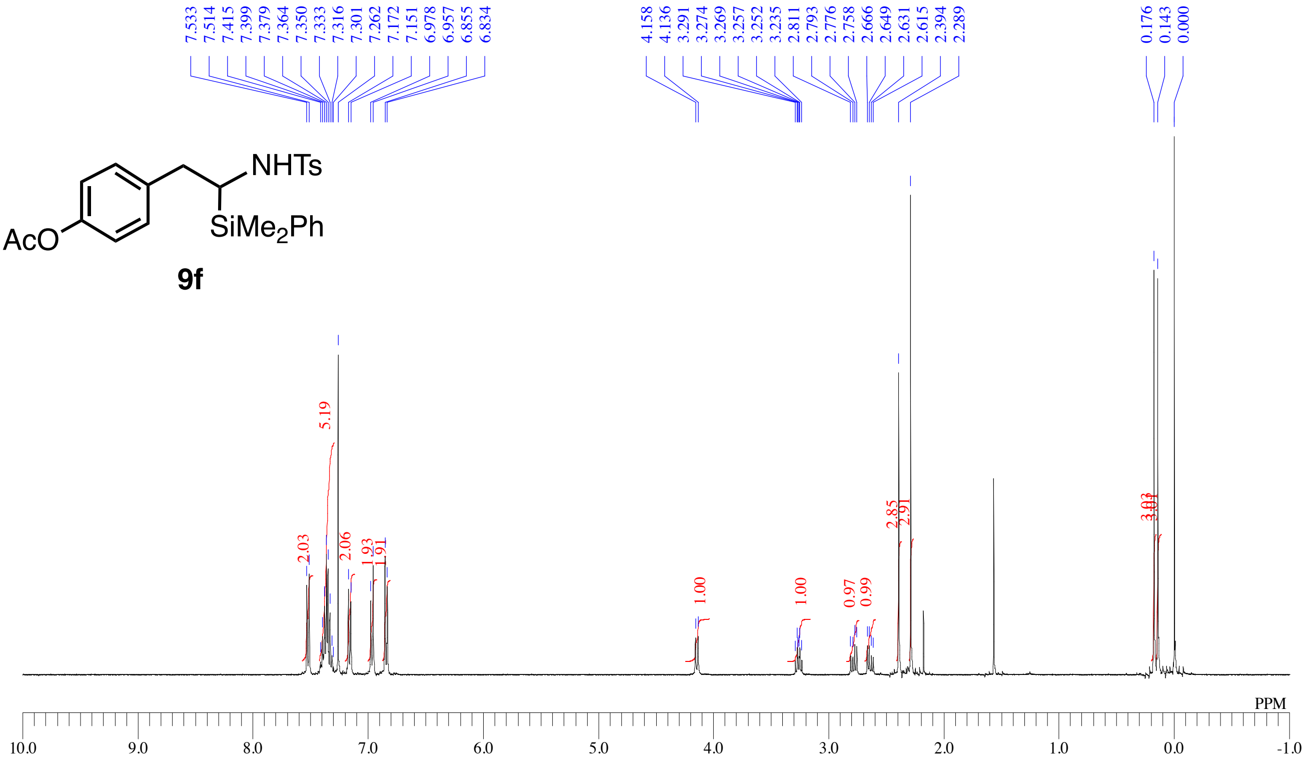

9f

^{13}C NMR: (100 MHz, $CDCl_3$)

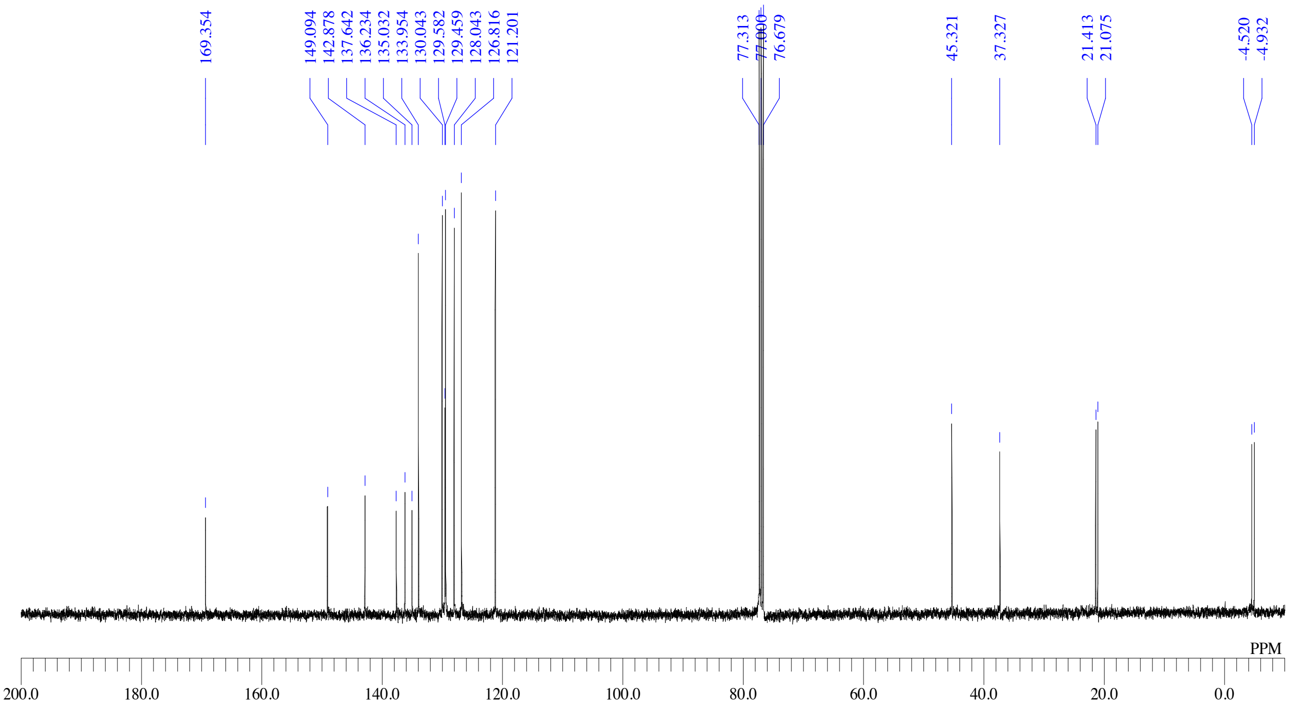

^{1}H NMR: (400 MHz, $CDCl_3$)

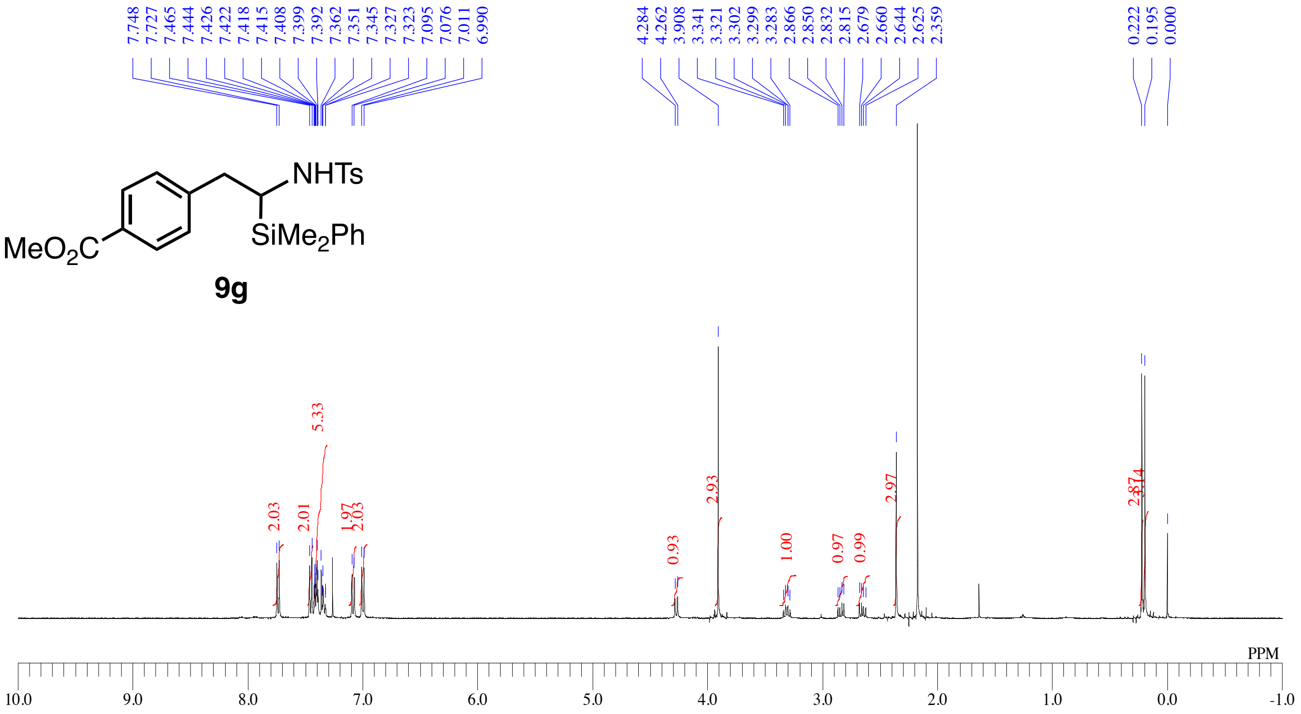

^{13}C NMR: (100 MHz, $CDCl_3$)

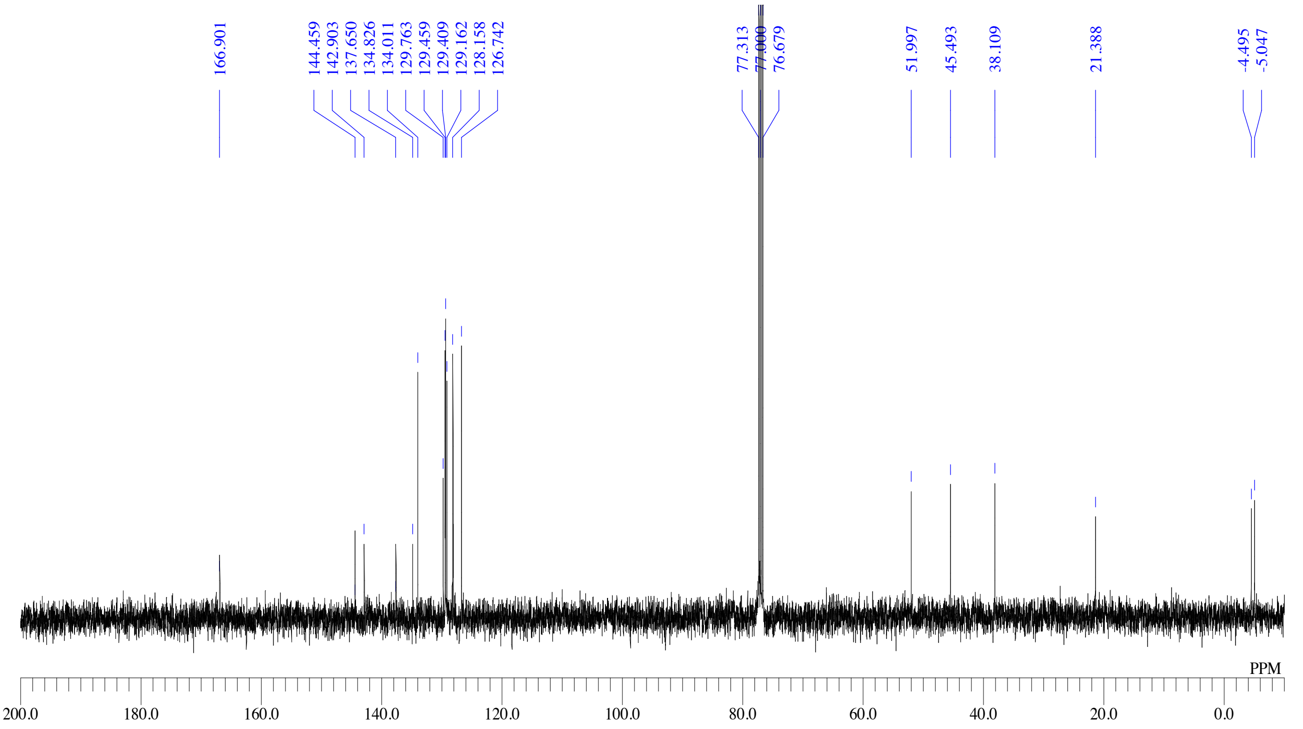

HPLC charts

(ChiralcelOJ; 1.0 mL/min; *i*-PrOH/*n*-hexane 30:70; λ = 254 nm)

<chrom atogram>

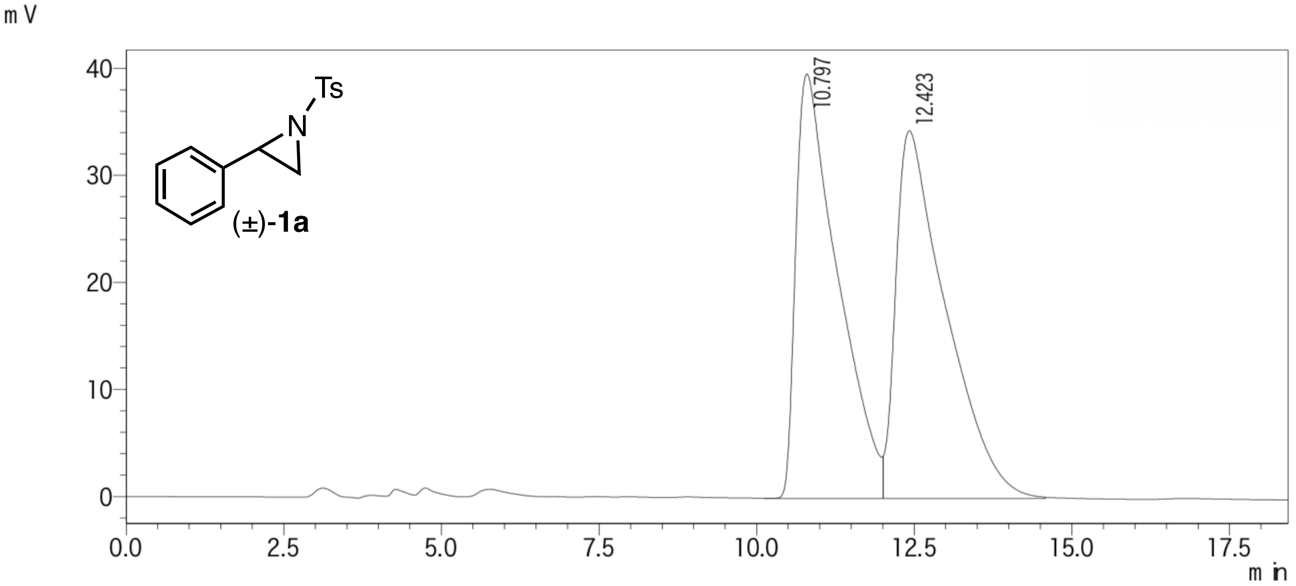

<peak report>

peak#	retanton tim e	area	he ight	area%
1	10.797	1833686	39683	49.091
2	12.423	1901562	34369	50.909
Total		3735248	74052	100.000

(ChiralcelOJ; 1.0 mL/min; *i*-PrOH/*n*-hexane 30:70; λ = 254 nm)

<chrom atogram>

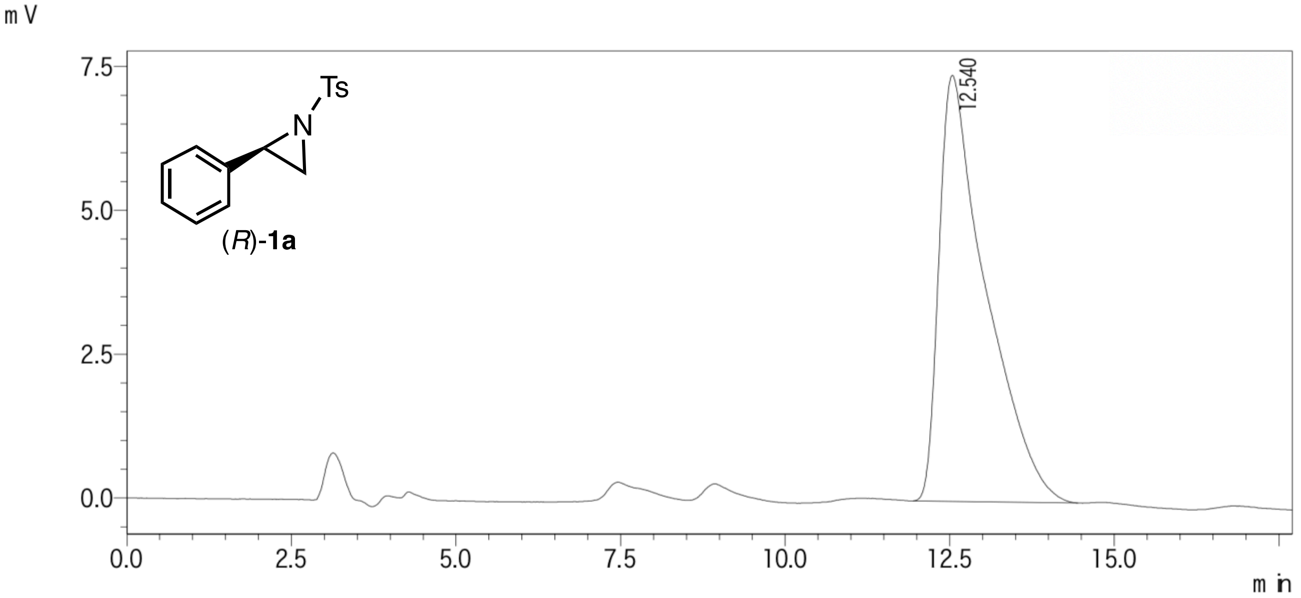

<peak report>

peak#	retantion tim e	area	he ight	area%
1	12.540	366916	7403	100.000
Total		366916	7403	100.000

(Chiralcel OJ; 1.0 mL/min; *i*-PrOH/*n*-hexane 30:70; λ = 254 nm)

<chromatogram>

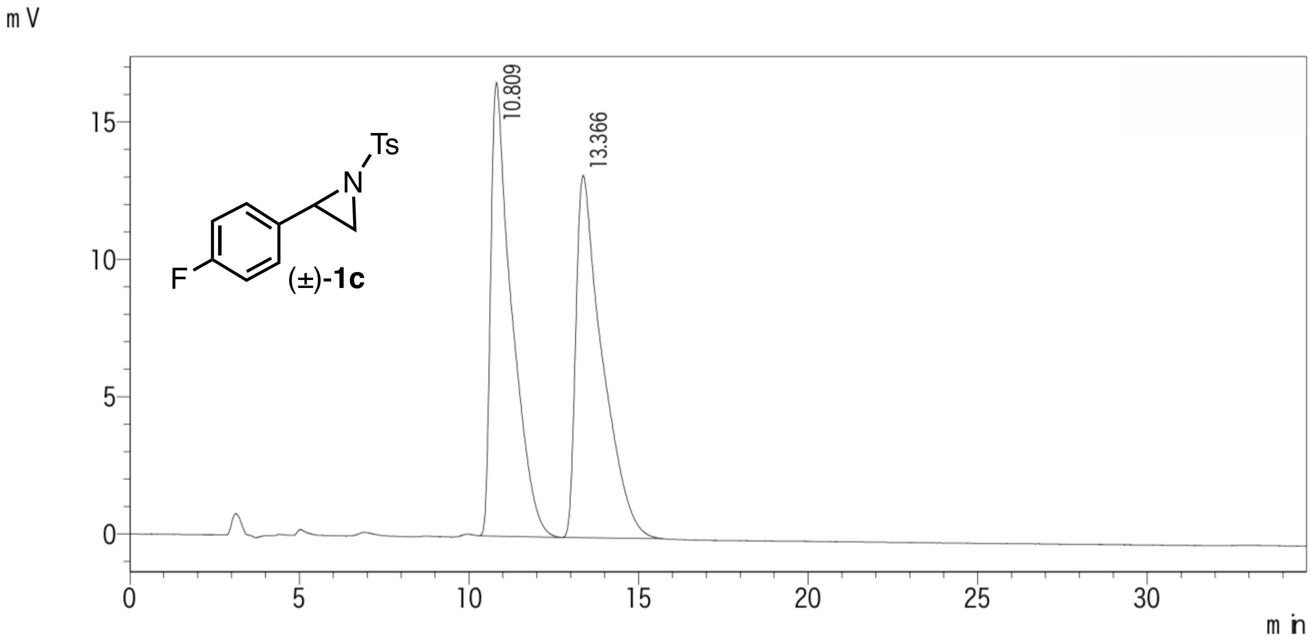

peak report

peak#	retantion time	area	height	area%
1	10.809	713586	16512	49.984
2	13.366	714037	13192	50.016
Total		1427623	29705	100.000

(Chiralcel OJ; 1.0 mL/min; *i*-PrOH/*n*-hexane 30:70; λ = 254 nm)

<chromatogram>

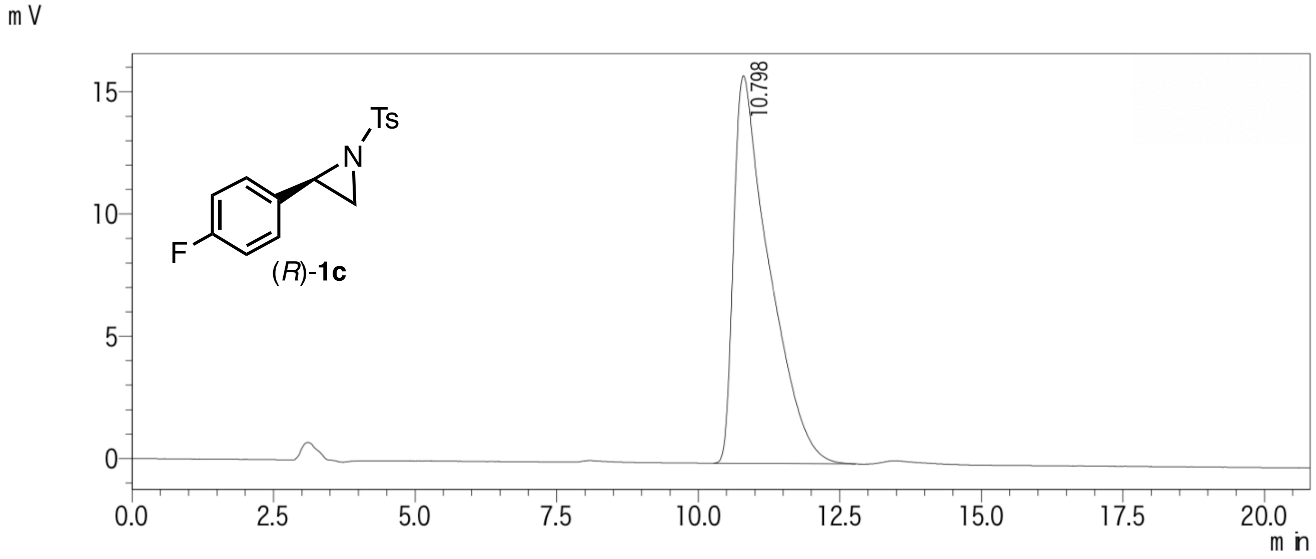

<peak report>

peak#	retantion time	area	height	area%
1	10.798	694806	15854	100.000
Total		694806	15854	100.000

(Chiralcel OJ; 1.0 mL/min; *i*-PrOH/*n*-hexane 30:70; λ = 254 nm)

<chromatogram>

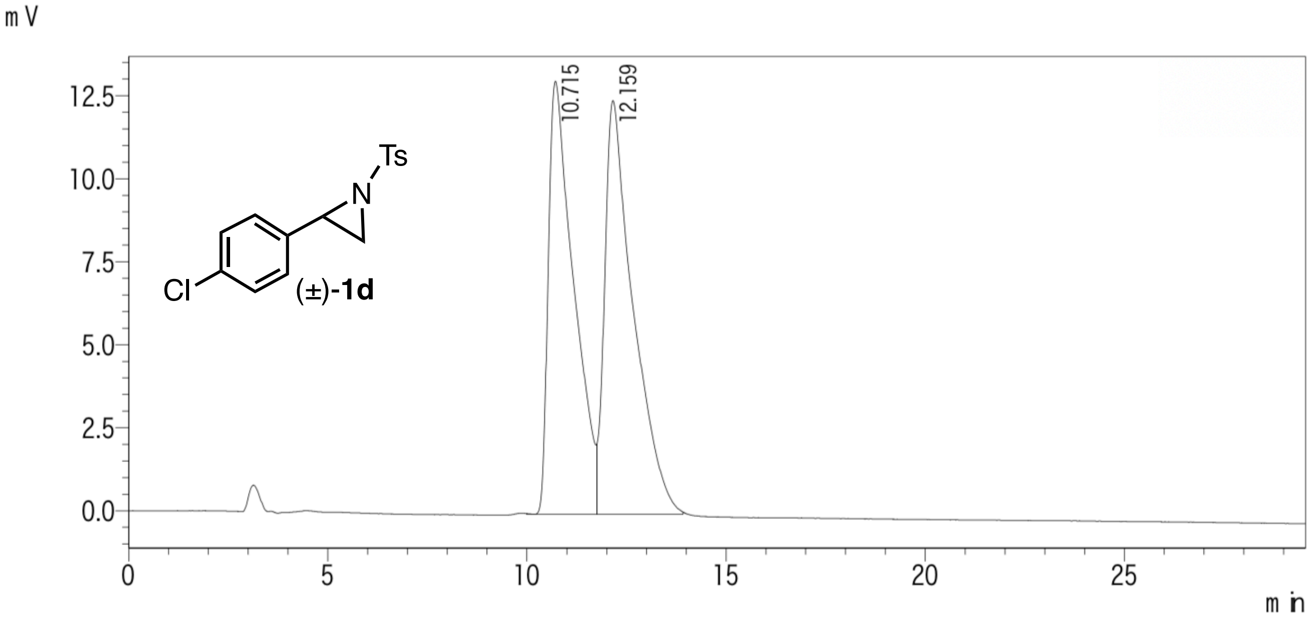

<peak report>

peak#	retantion time	area	height	area%
1	10.715	578691	13039	48.246
2	12.159	620761	12455	51.754
Total		1199453	25493	100.000

(Chiralcel OJ; 1.0 mL/min; *i*-PrOH/*n*-hexane 30:70; λ = 254 nm)

<chromatogram>

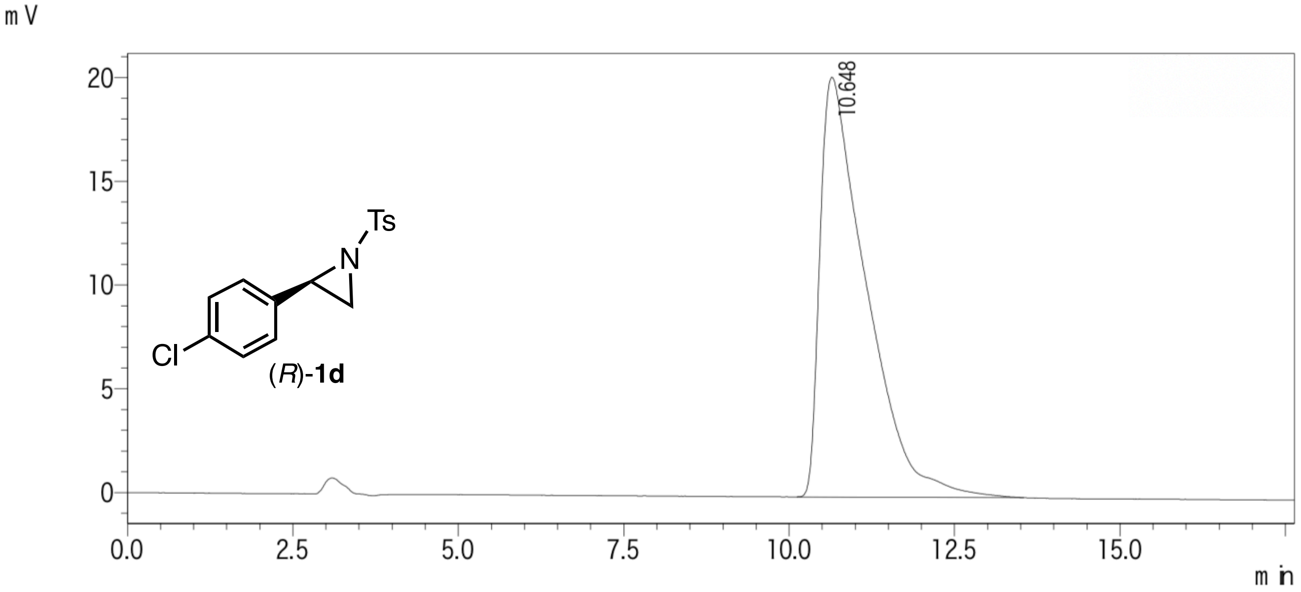

<peak report>

peak#	retantion time	area	height	area%
1	10.648	983278	20228	100.000
Total		983278	20228	100.000

(Chiralcel OJ; 1.0 mL/min; *i*-PrOH/*n*-hexane 10:90; λ = 254 nm)

<chromatogram>

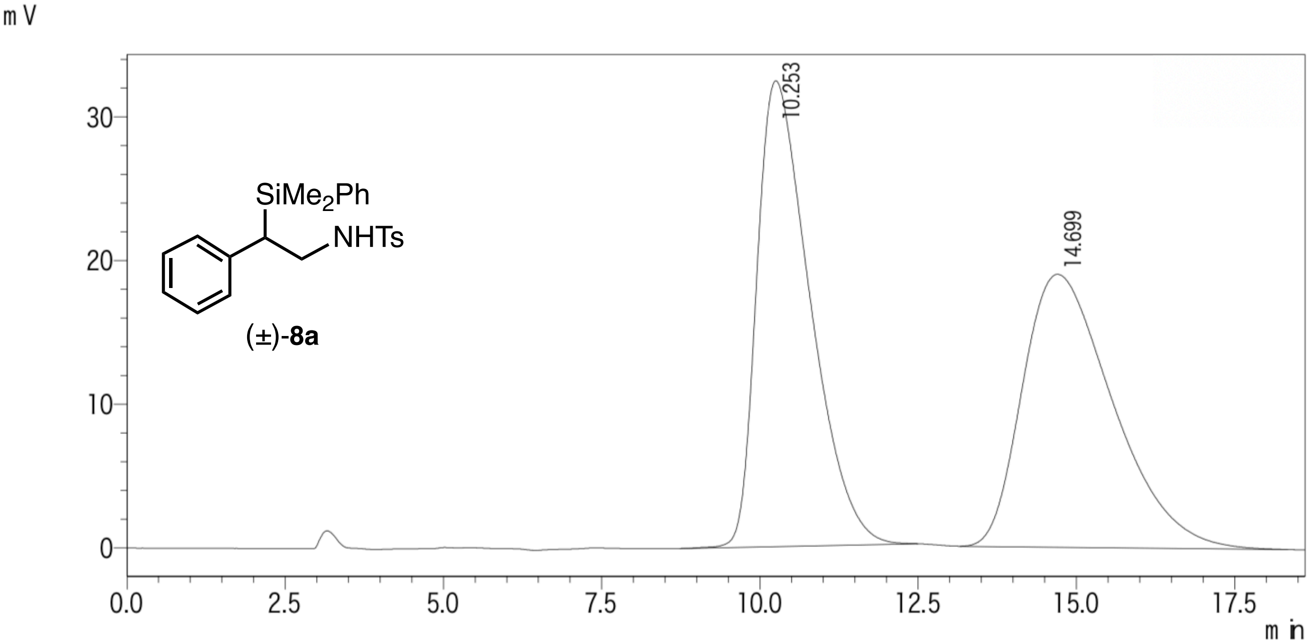

<peak report>

peak#	retantion time	area	height	area%
1	10.253	1896813	32402	50.143
2	14.699	1885981	18989	49.857
Total		3782794	51390	100.000

(Chiralcel OJ; 1.0 mL/min; *i*-PrOH/*n*-hexane 10:90; λ = 254 nm)

<chromatogram>

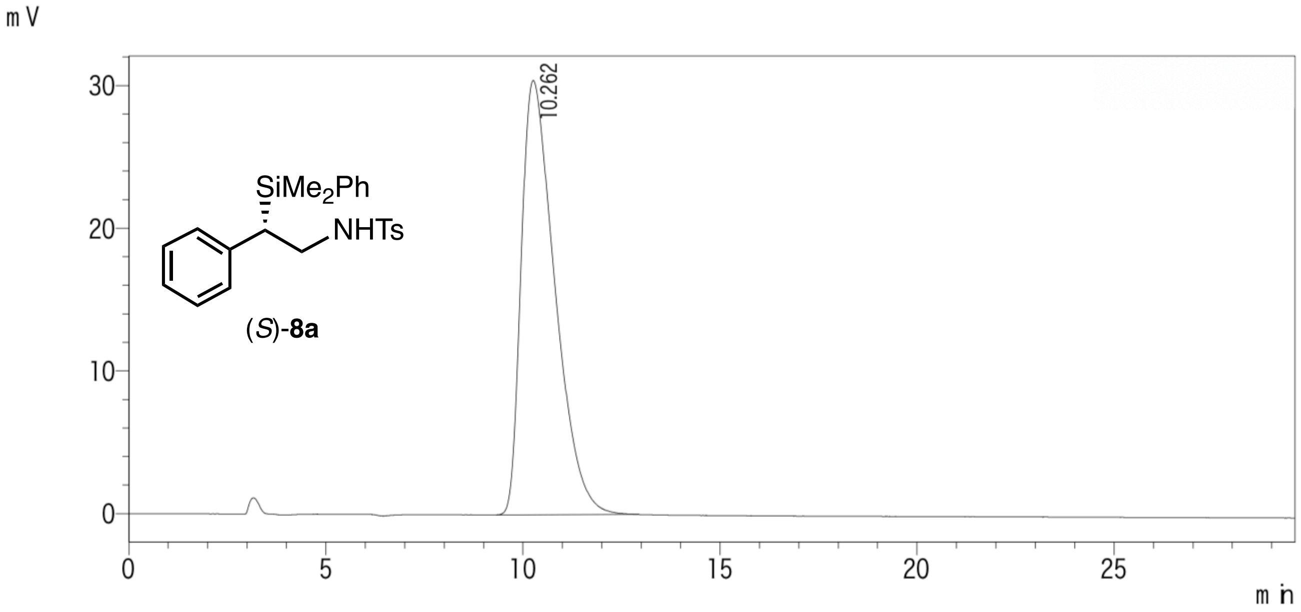

<peak report>

peak#	retantion time	area	height	area%
1	10.262	1806775	30442	100.000
Total		1806775	30442	100.000

(Chiralcel OJ; 1.0 mL/min; *i*-PrOH/*n*-hexane 10:90; λ = 254 nm)

<chromatogram>

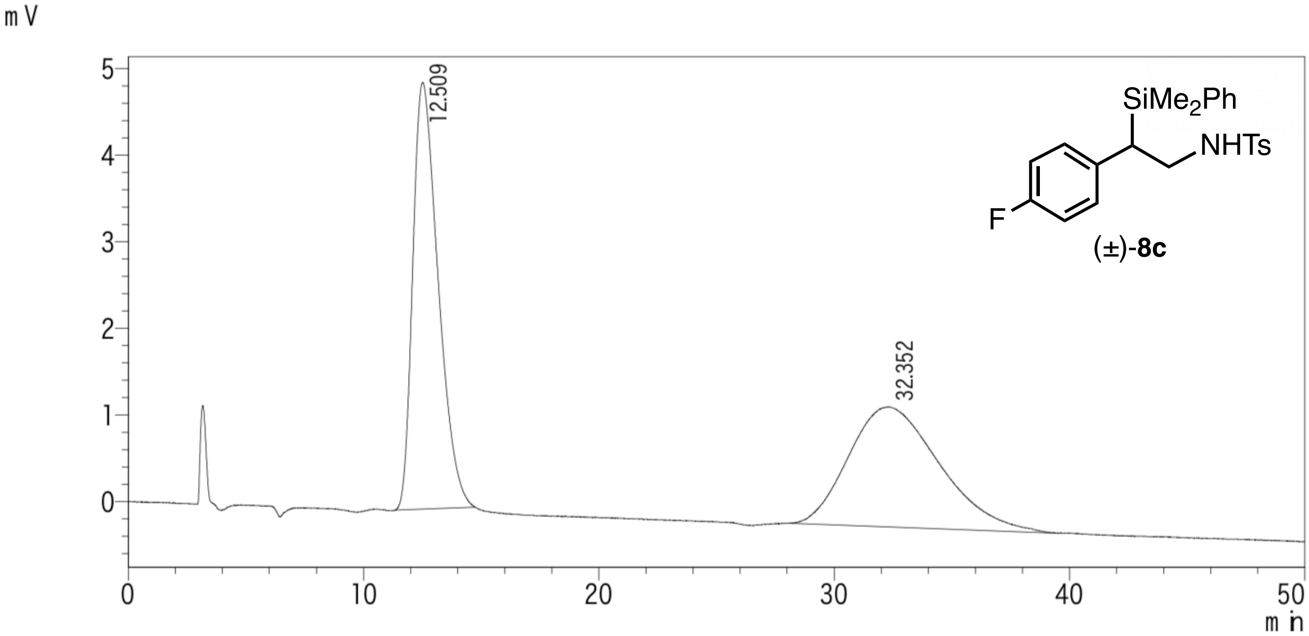

<peak report>

peak#	retantion time	area	height	area%
1	12.509	369093	4927	49.799
2	32.352	372069	1386	50.201
Total		741162	6313	100.000

(ChiralcelOJ; 1.0 mL/min; *i*-PrOH/*n*-hexane 10:90; λ = 254 nm)

<chromatogram>

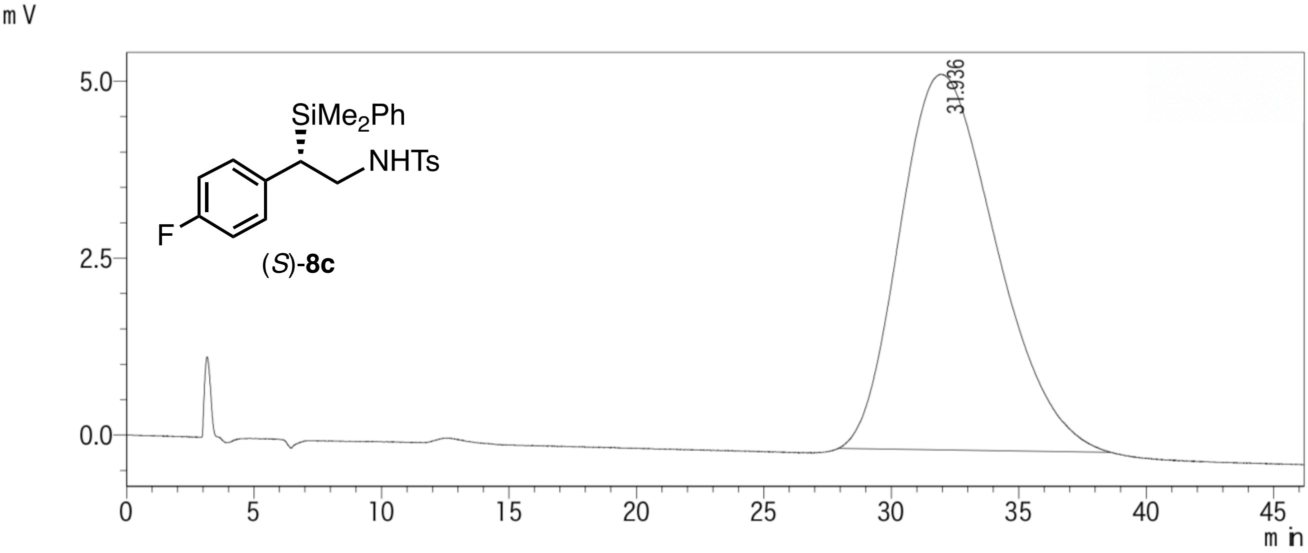

<peak report>

peak#	retantion time	area	height	area%
1	31.936	1398325	5307	100.000
Total		1398325	5307	100.000

(ChiralcelOJ; 1.0 mL/min; *i*-PrOH/*n*-hexane 10:90; λ = 254 nm)

<chromatogram>

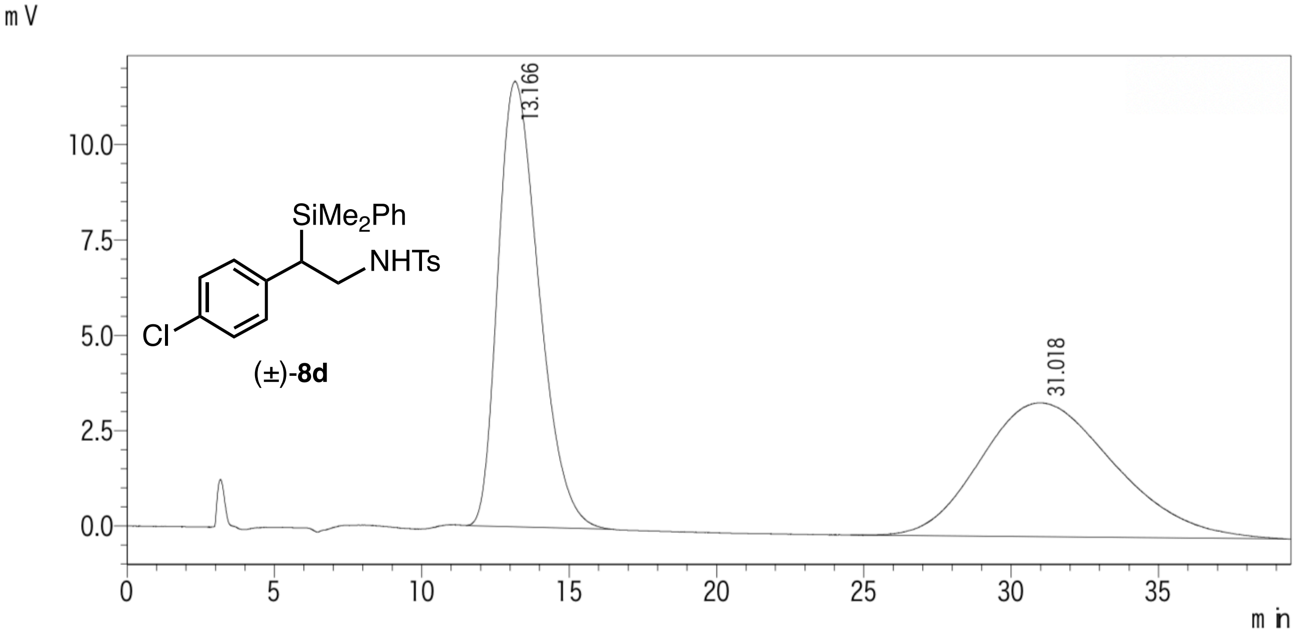

<peak report>

peak#	retantion time	area	height	area%
1	13.166	1128693	11679	50.353
2	31.018	1112860	3512	49.647
Total		2241553	15191	100.000

(ChiralcelOJ; 1.0 mL/min; *i*-PrOH/*n*-hexane 10:90; λ = 254 nm)

<chromatogram>

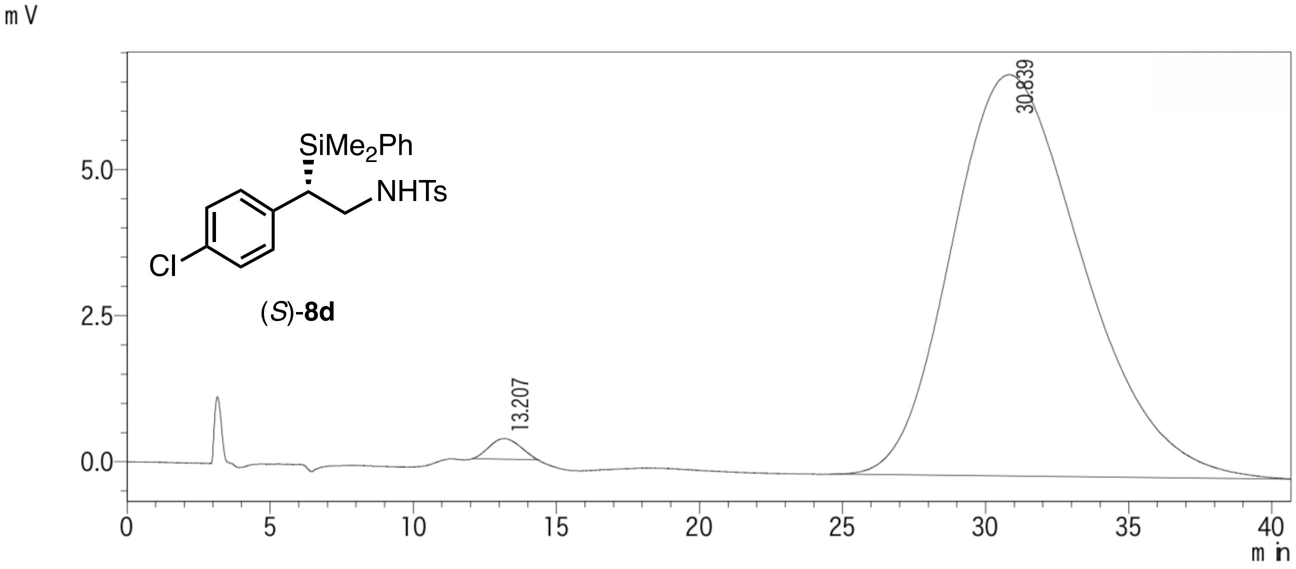

<peak report>

peak#	retantion time	area	height	area%
1	13.207	26504	355	1.195
2	30.839	2190900	6866	98.805
Total		2217404	7221	100.000

References

S1. (a) *The Fifth Series of Experimental Chemistry*: *Vol. 21 Organotransition-metal Compounds and Supramolecular Complexes.* The Chemical Society of Japan, Tokyo, Japan, 2004. (b) Tatsuno, Y.; Yoshida, T.; Otsuka, S. *Inorg. Synth.* **1979**, *19*, 220–223.

S2. Nicholai, S.; Sedigh-Zadeh, R.; Waser, J. *J. Org. Chem.* **2013**, *78*, 3783–3801.

S3. Fantasia, S.; Nolan, P. S. *Chem. Eur. J.* **2008**, *14*, 6987 – 6993.

S4. Kano, D.; Minakata, S.; Komatsu, M.; *J. Chem. Soc., Perkin Trans. 1* **2001**, 3186–3188.

S5. Evans, D. A.; Faul, M. M.; Bilodeau, M. T. *J. Am. Chem. Soc.* **1994**, *116*, 2742–2753.

S6. Dauban, P.; Saniere, L.; Tarrade, A.; Dodd, R. H. *J. Am. Chem. Soc.* **2001**, *123*, 7707–7708.

S7. Gao, G.-Y.; Harden, J. D.; Zhang, X. P. *Org. Lett.* **2005**, *7*, 3191–3193.

S8. Ghorai, M. S.; Nanaji, Y. *J. Org. Chem.* **2013**, *78*, 3867–3878.

S9. Seayad, J.; Seayad, A. M.; Ng, J. K. P.; Chai, C. L. L. *ChemCatChem* **2012**, *4*, 774–777.

S10. Ando, T.; Kano, D.; Minakata, S.; Ryu, I.; Komatsu, M. *Tetrahedron* **1998**, *54*, 13485–13494.

S11. Cui, Y.; He, C. *J. Am. Chem. Soc.* **2003**, *125*, 16202–16203.

S12. Jin, W.; Li, X.; Huang, Y.; Wu, F.; Wan, B. *Chem. Eur. J.* **2010**, *16*, 8259–8261.

S13. Jeffs, L.; Arquier, D.; Kariuki, B.; Bethell, D.; Page, P. C. B.; Hutchings, G. J. *Org. Biomol. Chem.* **2011**, *9*, 1079–1084.

S14. Aggarwal, V. K.; Ferrara, M. *Org. Lett.* **2000**, *2*, 4107–4110.

S15. Rigaku Oxford Diffraction (2015), Software CrysAlisPro 1.171.39.5a Rigaku Corporation, Tokyo, Japan

S16. Sheldrick, G. M. *Acta Cryst.* **2014**, *A70*, C1437.

S17. CrystalStructure 4.2.5: Crystal Structure Analysis Package, Rigaku Corporation (2000-2017). Tokyo 196-8666, Japan.

S18. SHELXL Version 2014/7. Sheldrick, G. M. *Acta Cryst.* **2008**, *A64*, 112–122.
